# Catalysis of multi-component syntheses by triarylmethyl chlorides

**DOI:** 10.1039/d6ra07161a

**Published:** 2026-09-01

**Authors:** Ahmad Reza Moosavi-Zare, Abdolkarim Zare

**Affiliations:** a Chemistry Department, College of Sciences, Shiraz University Shiraz 71946-84795 Iran moosavizare@yahoo.com; b Department of Chemistry, Faculty of Nano and Bio Science and Technology, Persian Gulf University Bushehr Iran

## Abstract

Triarylmethyl chlorides, through *in situ* generation of the corresponding triarylmethyl carbocations, act as mild homogeneous Lewis acid catalysts and effectively promote a wide range of organic transformations. In this review, examples of multicomponent organic syntheses catalyzed by triarylmethyl carbocations, with particular emphasis on their distinctive catalytic mechanisms, are discussed. Metal-free, homogeneity, inherent mild Lewis acidity and neutral media of the reactions are some significant benefits of this carbocationic catalytic system.

## Abstract 2

Triarylmethyl chlorides, through *in situ* generation of the corresponding triarylmethyl carbocations, act as mild homogeneous Lewis acid catalysts and effectively promote a wide range of organic transformations.

## Graphical abstract

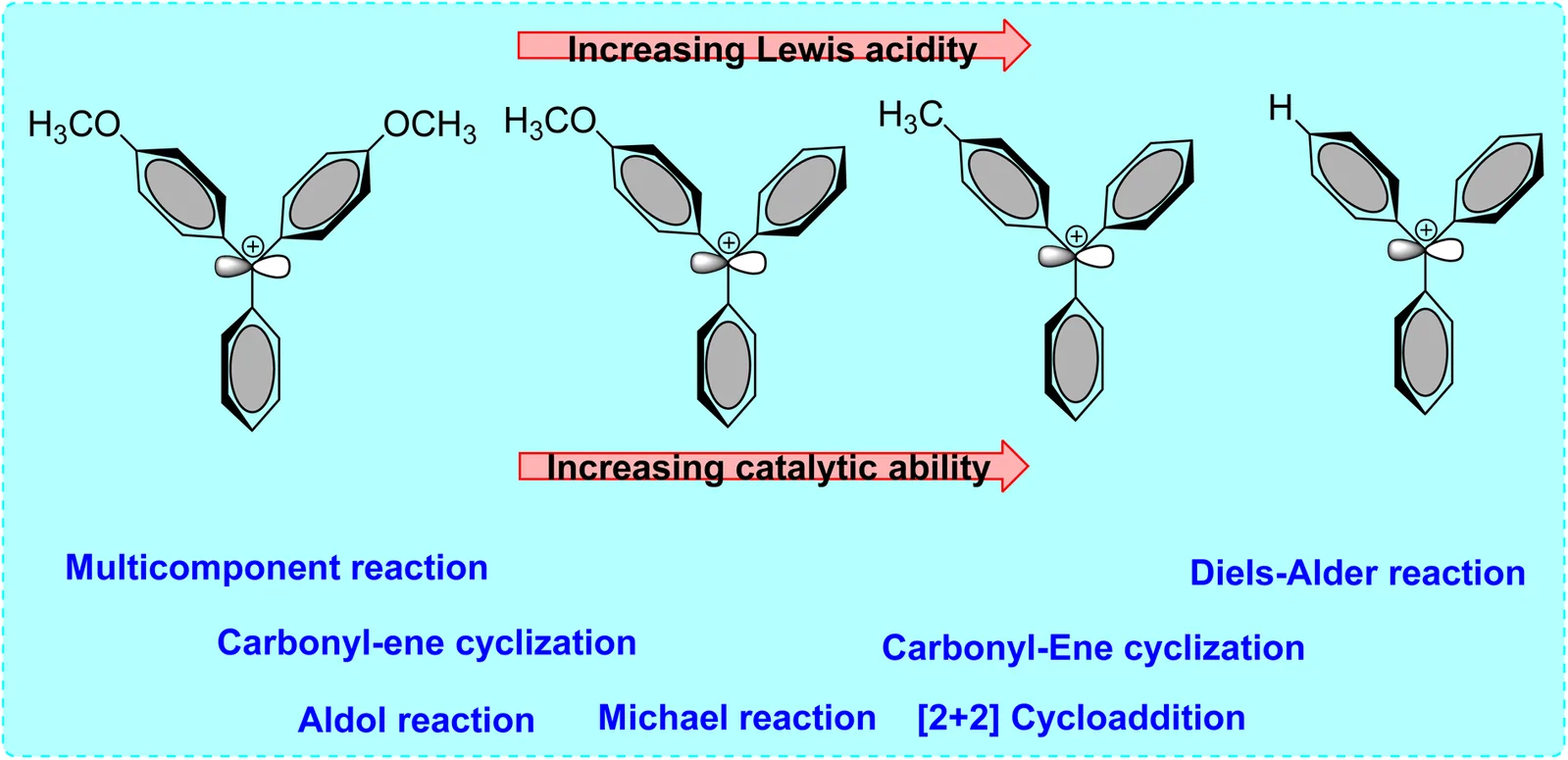

## Introduction

Carbocations are short-lived, highly reactive species that arise *in situ* during some organic reactions and are generally too unstable to be separated under conventional experimental conditions. The partial and inherent stability of these species can be slightly changed in a desirable way by making changes in their structure in such a way that, due to the empty orbital at the carbocation center, they can catalyze reactions in some organic reactions, like Lewis acid reactions. Some of the results obtained from the selective catalysis of these species in organic reactions are not comparable to traditional Lewis acids and are unique. Mild conditions and low reaction temperatures in carbocationic environments are other advantages of using these species for the purpose of catalyzing organic reactions.^1^

Carbocations have attracted considerable attention because of their pivotal role as reactive intermediates in organic reactions and their importance in the synthesis of organic molecules. The development of catalytic processes in organic synthesis is essential for the preparation of pharmaceuticals, polymers and other functional materials. In recent years, these active species have been used as general Lewis acid catalysts in the advancement and acceleration of organic reactions. In recent years, these intermediate compounds have been used in the field of catalysis of reactions due to the presence of an empty orbital in their structure. The acidity of carbocations can be modulated through structural modifications, and their catalytic activity is closely correlated with their stability. The mild acidity of carbocations and their production from neutral organic precursors in the reaction medium are very important in preserving the environment and its safety. The absence of metals in the carbocation structure prevents the inherent toxicity caused by the presence of some metals in traditional Lewis acids from being observed in the use of these intermediates. The co-phase behavior of carbocations with the starting materials, along with the formation of a homogeneous reaction medium and their good solubility in common organic solvents, facilitate both their application as catalysts and their separation from the reaction system.^2^

The application of neutral organic catalysts in place of conventional inorganic Lewis and Brønsted acids offers distinct advantages. In reactions where the starting materials are acid-sensitive and are degraded in the presence of acid or have basic functional groups, the importance of neutral organic catalysts becomes more apparent. Trityl chloride catalyzes organic reactions by creating a trityl carbocation in the reaction medium.^3^

### Catalytic performance of trityl chloride

**Scheme 1:**
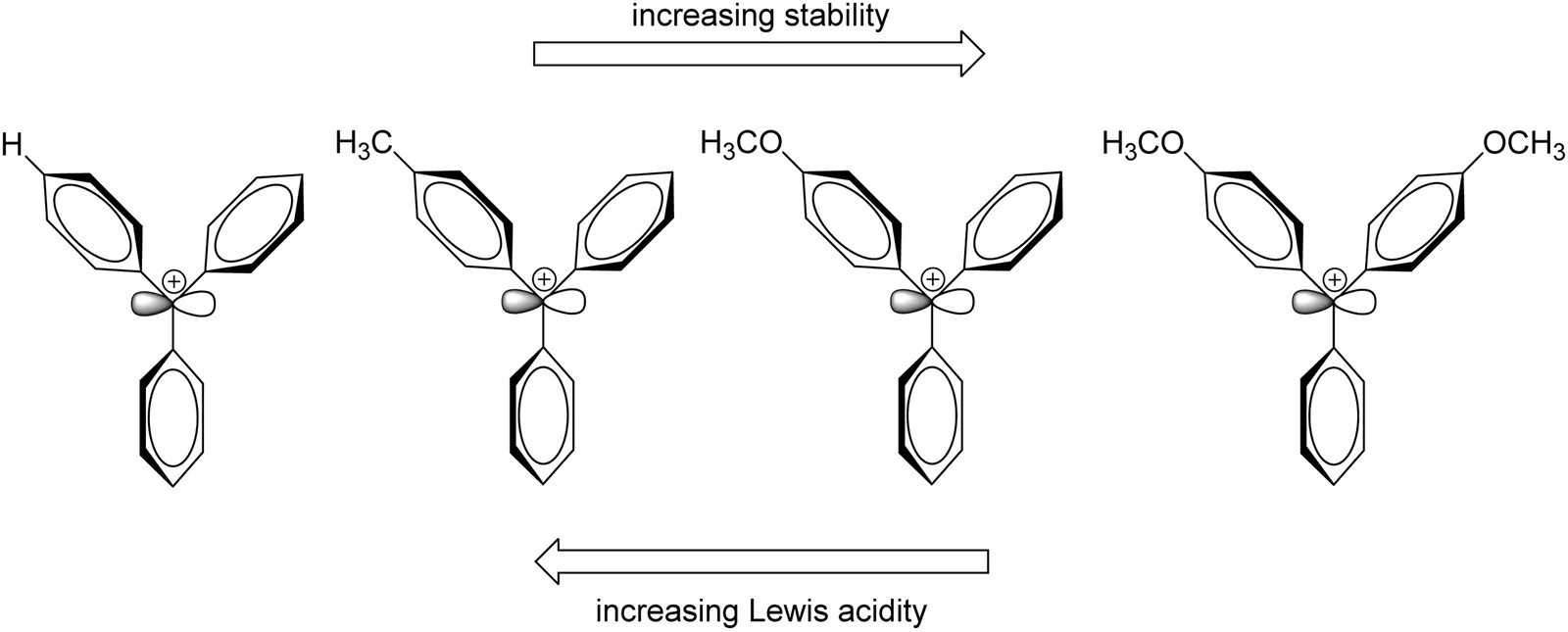
Comparing stability and catalytic ability of trityl derivatives.

The catalytic performance of triphenylmethyl chloride (trityl chloride, TrCl) has been evaluated in comparison with related derivatives, including 4,4′-dimethoxytrityl chloride (DMTrCl), 4-monomethoxytrityl chloride (MMTrCl) and trityl alcohol. In this study, TrCl generally exhibits the highest catalytic performance among the investigated catalysts. Electron-donating substituents on the phenyl rings of TrCl enhance carbocation stabilization, thereby decreasing its acidity strength.^4^ The relative acidity and stability of selected trityl-derived carbocations are illustrated in Scheme 1.

**Scheme 2:**
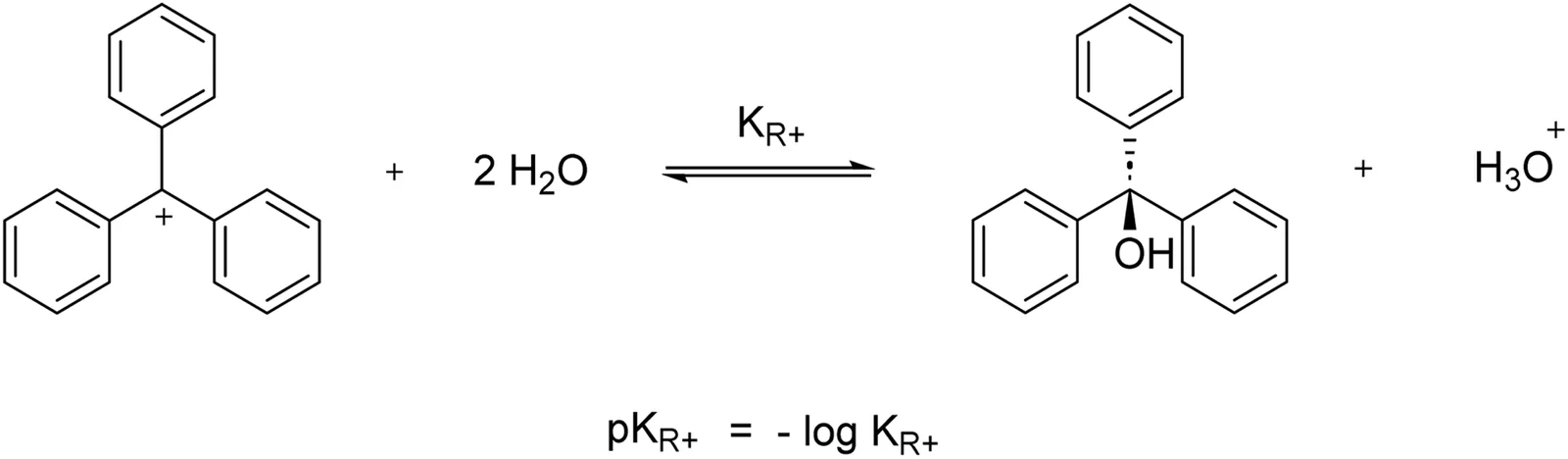
Equilibrium reaction of trityl carbocation (1 eq.) and water (2 eq.).

The electronic influence of substituents on aromatic rings can be readily evaluated by comparing the Lewis acidity of different trityl carbocations, which is quantified using their p*K*_R^+^_ values. The *K*_R^+^_ value corresponds to the equilibrium constant for the reaction between one equivalent of a carbocation and two equivalents of water. More than 60 years ago, p*K*_R^+^_ values were reported as a parameter for a reference system for comparing the stability of different carbocations in solution. This comparison provides a measure of the relative acidity of different carbocations. A larger equilibrium constant for the reaction indicates a higher acidity of the carbocation and correspondingly lower stability (Scheme 2).^4^

**Scheme 3:**
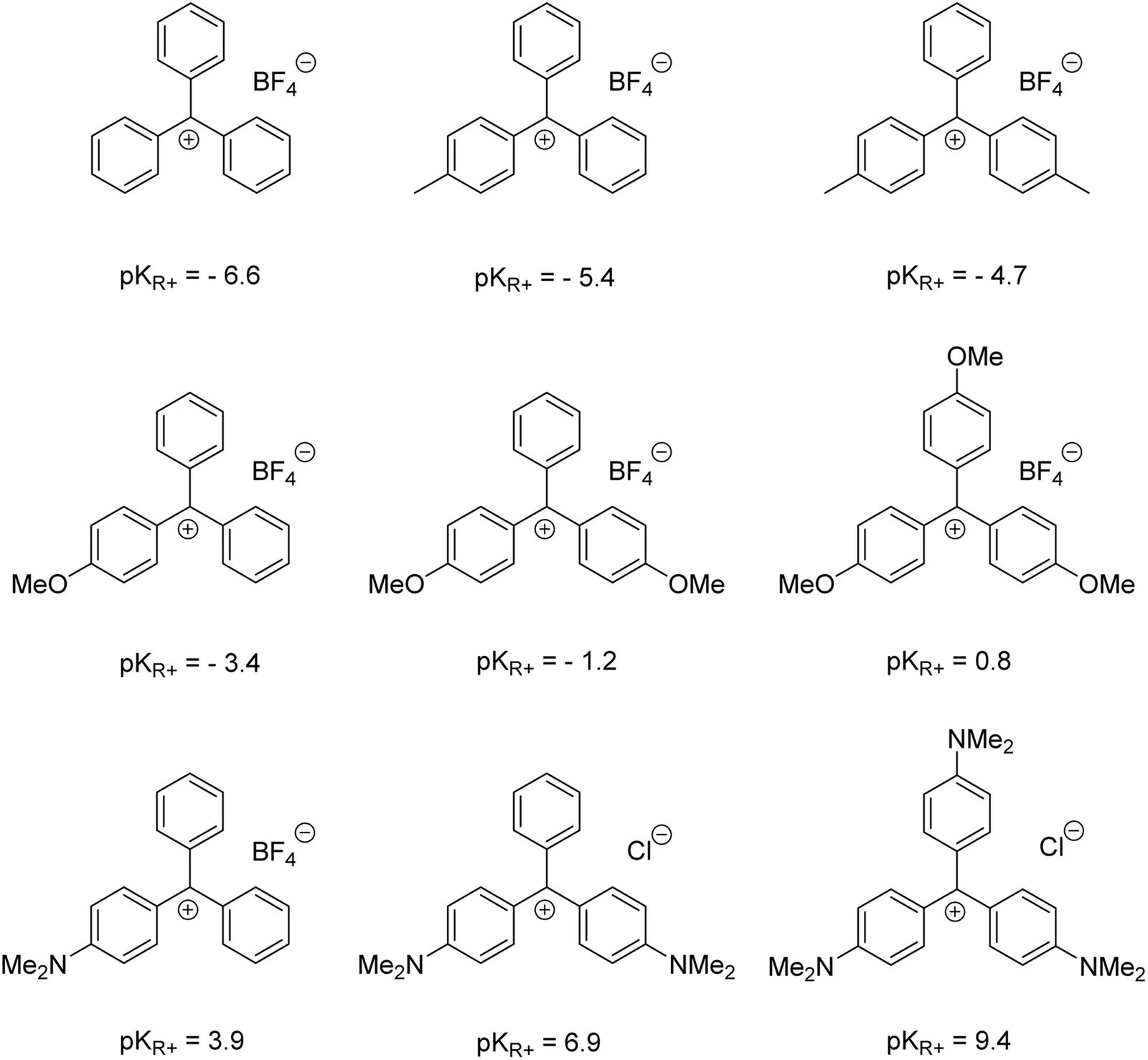
Comparison of Lewis acidity and relative stability of trityl carbocation derivatives.

In Scheme 3, the p*K*_R^+^_ for various trityl carbocations is given and the relative Lewis acidity between them is compared. A smaller p*K*_R^+^_ indicates a higher Lewis acidity and a less stable carbocation species.^4^

### The pseudo three-component fabrication of bis(indolyl)methanes

**Scheme 4:**
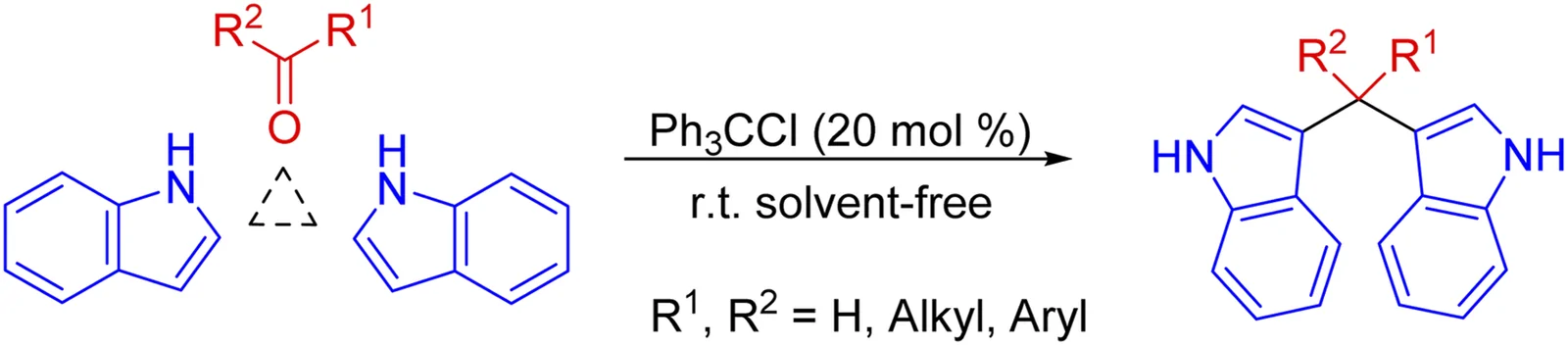
The construction of bis(indolyl)methanes using trityl chloride.

Bis(indolyl)methane derivatives have been fabricated under mild, solvent-free conditions at room temperature through a pseudo three-component process, in which 2 equiv. of indole react with 1 equiv. of an aldehyde in the presence of trityl chloride (TrCl) acting as a neutral catalyst (Scheme 4).^5^

**Scheme 5:**
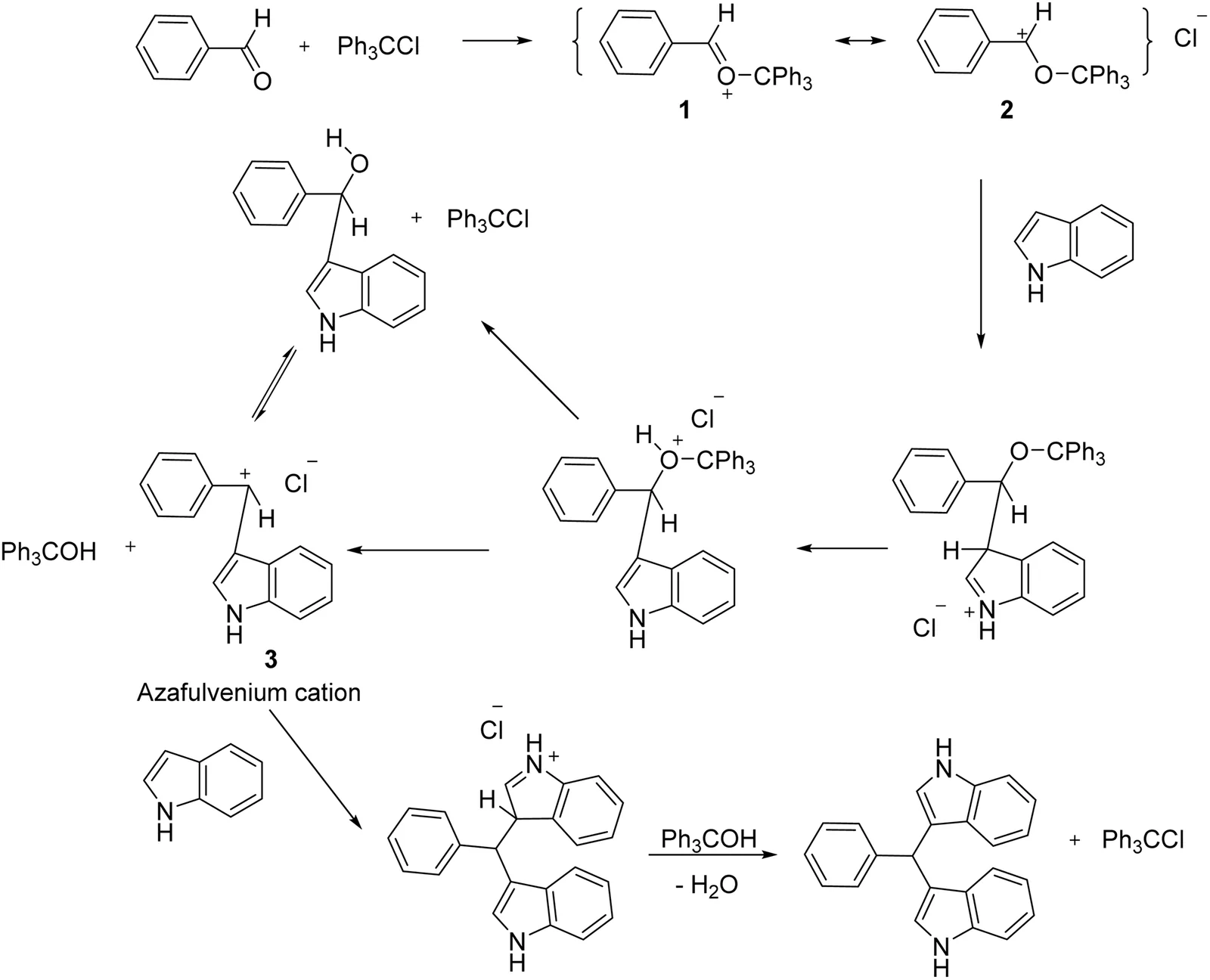
Mechanistic pathway for the fabrication of bis(indolyl)methanes catalyzed by TrCl.

In this reaction, the trityl carbocation, with its partial stability, acts as a Lewis acid and activates the aldehyde carbonyl group by forming complexes 1 and 2. The trityl cation-activated forms of aldehyde (1 and 2) were first proposed by Oikawa *et al.*.^6^ The first indole acts as a nucleophile and undergoes nucleophilic attack to the carbonyl group at the C_3_ position, producing the azafulonium carbocation (3). The second indole molecule then attacks the cation, producing the desired bis(indolyl) methane. Finally, the trityl alcohol is converted to TrCl by removing a water molecule and absorbing a chloride anion from the reaction medium (Scheme 5).^5^

In another study, other trityl derivatives including monomethoxytrityl chloride, dimethoxytrityl chloride and triphenylmethanol were used as catalysts in the reaction of 1*H*-indole (2 mmol), with benzaldehyde (1 mmol) in the absence of solvent as a model reaction which did not improve the yield of related product in comparison with trityl chloride. The placement of methoxy groups on the aromatic ring in the catalyst structure can reduce the positive charge of the triaryl carbenium ion, thereby reducing its Lewis acidity and catalytic ability.^5^

### The pseudo three-component synthesis of bis(pyrazolyl)methanes

**Scheme 6:**
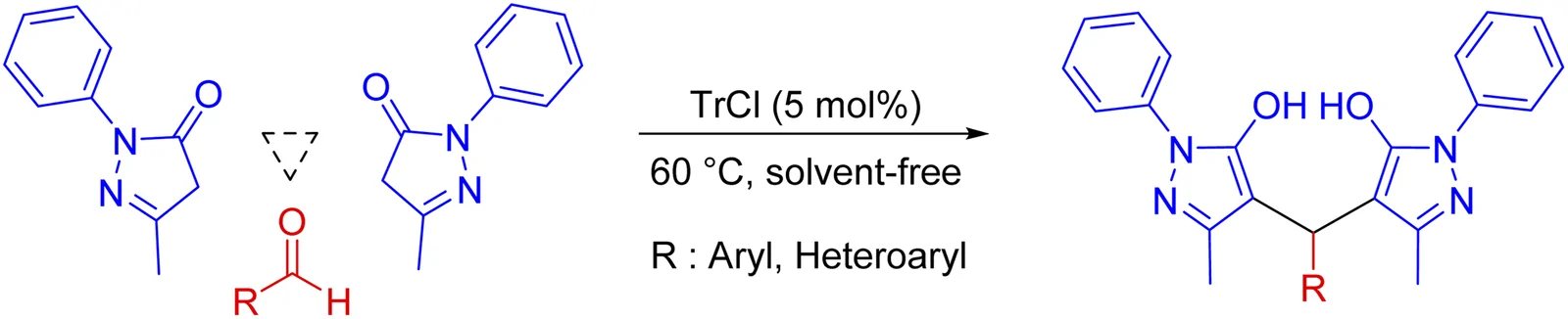
TrCl-mediated fabrication of bis(pyrazolyl)methanes.

In another process, TrCl has been utilized as a catalyst in the pseudo-three component construction of bis(pyrazolyl)methane compounds from the reaction of 3-methyl-1-phenylpyrazolone (2 equiv.) with aldehyde (1 equiv.) under mild and solvent-free conditions at 60 °C (Scheme 6).^7^

**Scheme 7:**
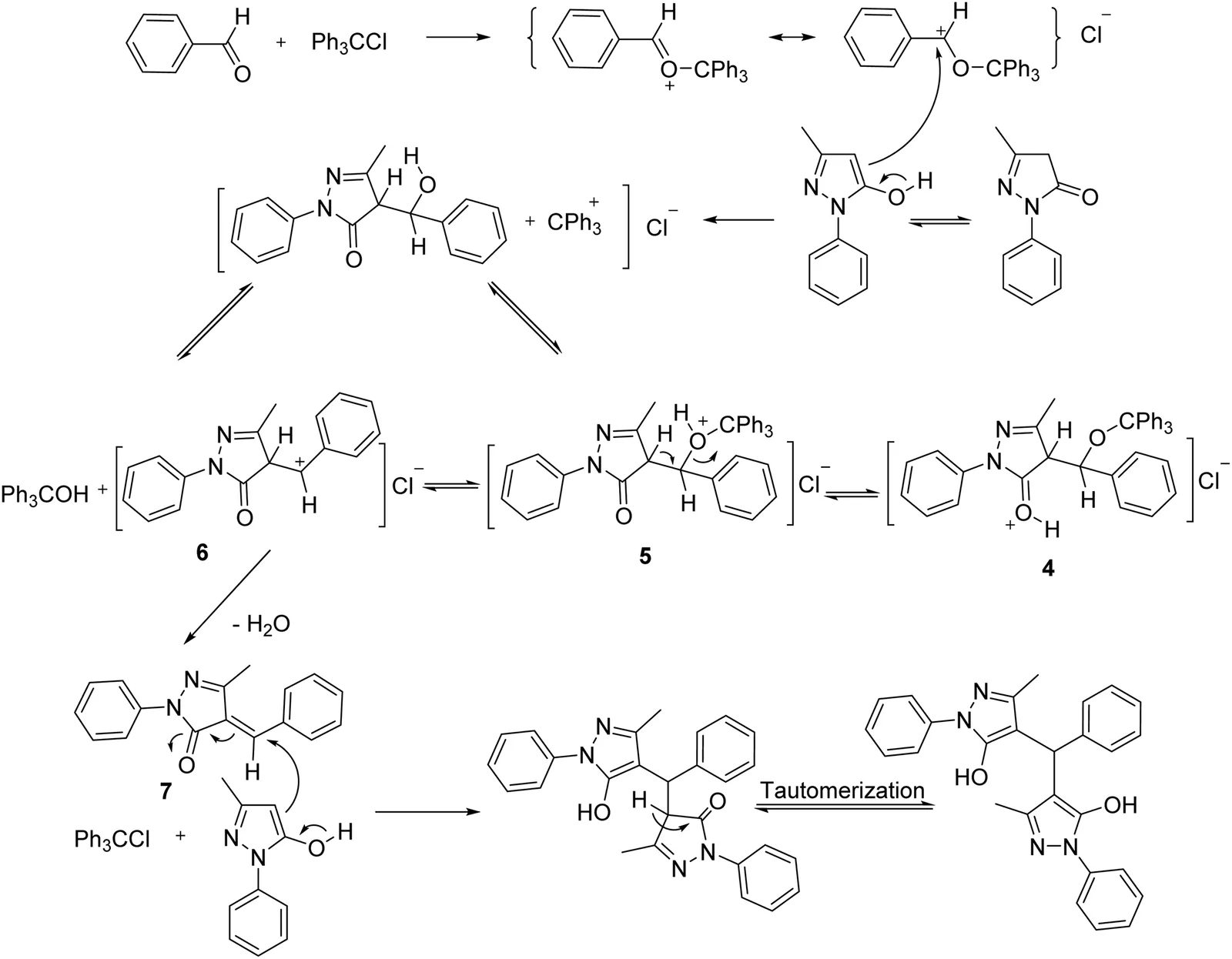
Mechanism of the fabrication of bis(pyrazolyl)methane compounds utilizing TrCl.

In the above transformation, activation of the aldehyde carbonyl group occurs *via* interaction with the trityl carbocation, and one equivalent of 3-methyl-1-phenylpyrazolone reacts with it after undergoing tautomerization to give intermediates (4) to (6). Then, by removing one molecule of water from (6) and regenerating the catalyst, intermediate (7) is produced as a Michael acceptor. In the next step, another equivalent of 3-methyl-1-phenylpyrazolone undergoes a Michael reaction with intermediate (7) and then undergoes tautomerization prepared the related bis (pyrazolyl)methane. By absorbing the chloride anion by trityl alcohol and removing a water molecule, TrCl is regenerated in the reaction medium (Scheme 7).^7^

### The three-component synthesis of 1-amidoalkyl-2-naphthol

**Scheme 8:**
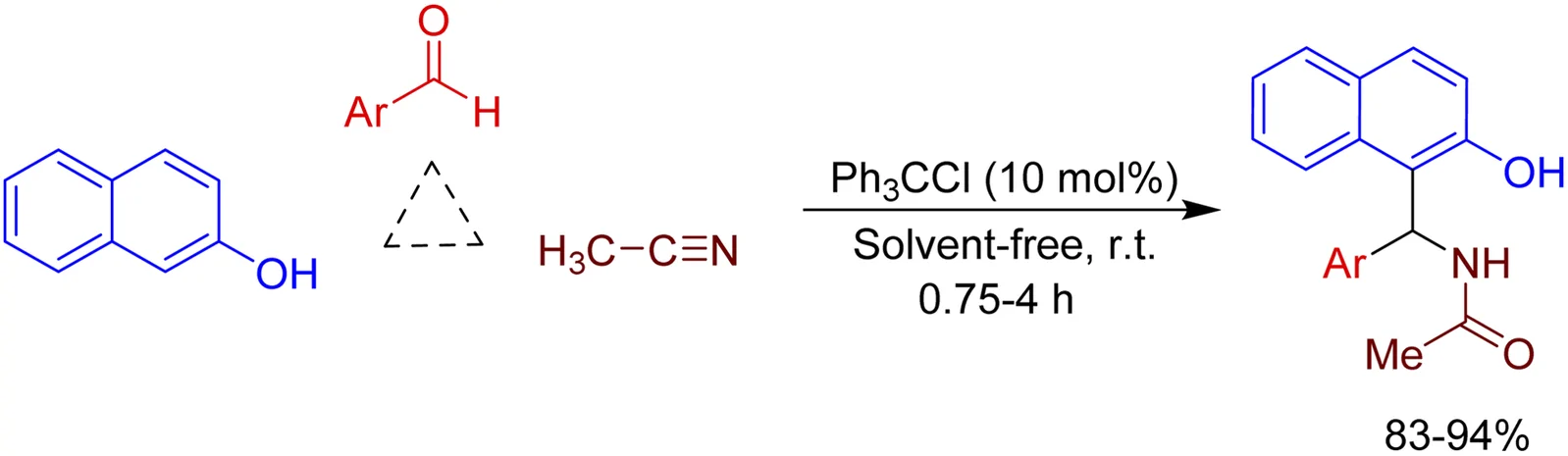
TrCl-catalyzed fabrication of 1-amidoalkyl-2-naphthol derivatives.

In another synthesis, TrCl has been employed as a mild, homogeneous organic catalyst for the room-temperature synthesis of 1-amidoalkyl-2-naphthol derivatives *via* a three-component reaction involving an aldehyde, 2-naphthol and acetonitrile in a neutral environment under solvent-free condition (Scheme 8).^8^

**Scheme 9:**
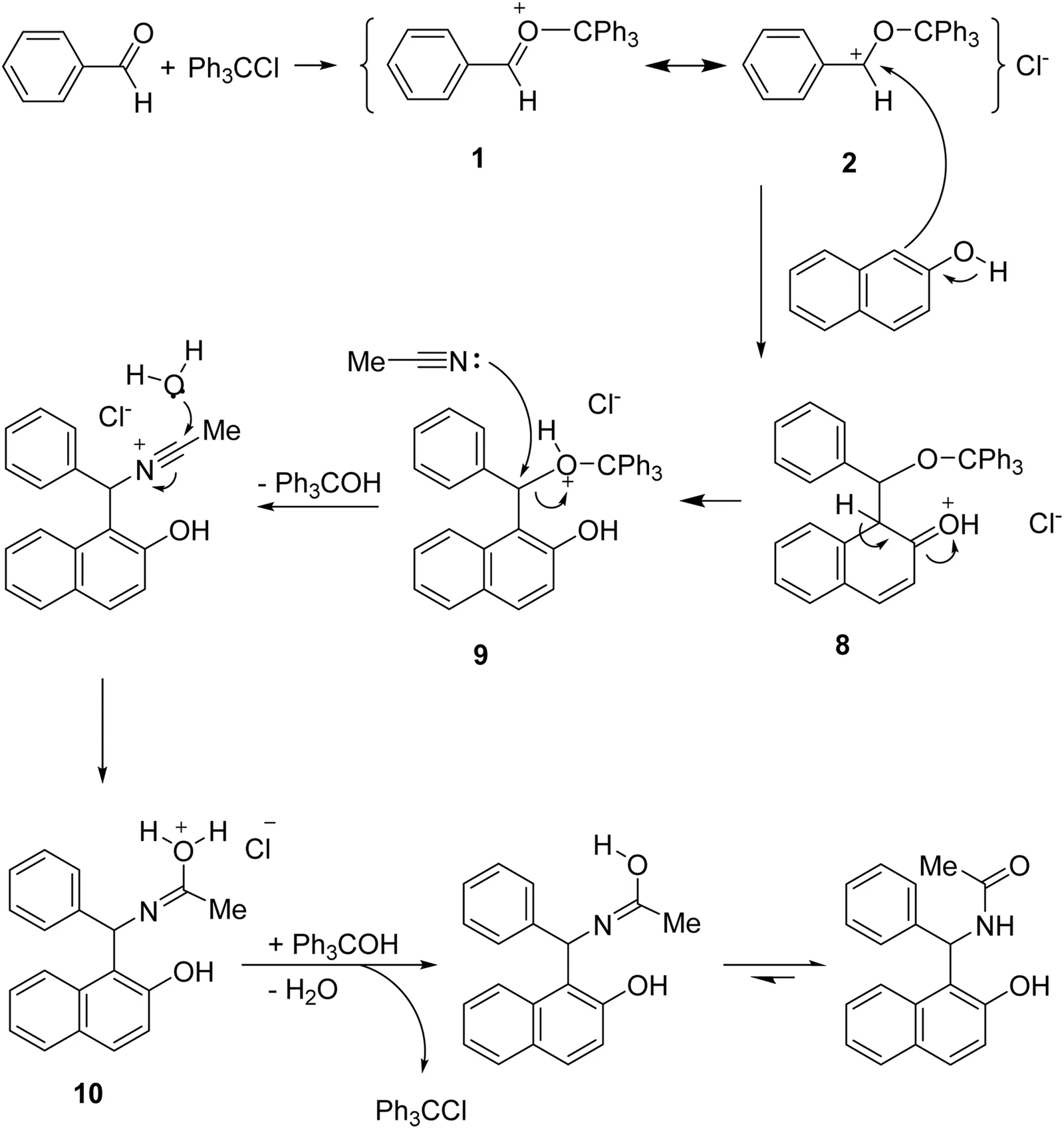
Mechanistic pathway for the formation of 1-amidoalkyl-2-naphthol compounds utilizing TrCl.

In the investigation of the mechanism of this reaction, the aldehyde is first activated by the trityl cation. These complexes (1 and 2) act like activated carbonyl forms and react with 2-naphthol to produce intermediate (8), which is converted to intermediate (9) by hydrogen transfer. Intermediate (9) reacts with acetonitrile and undergoes a Ritter reaction to form compound (10) which is converted to 1-amidoalkyl-2-naphthol as the main product (Scheme 9). It should be noted that the method is not very general because replacing benzonitrile with acetonitrile does not lead to the formation of 1-amidoalkyl-2-naphthols.^8^

To show the treatment of benzaldehyde with Ph_3_CCl and increase the electrophilicity of carbonyl bond, FT-IR, ^1^H NMR, and UV spectra of the resulting mixture were compared with this bond in benzaldehyde. According that, carbonyl bond (C=O) vibration in benzaldehyde decreased from 1705 cm^−1^ to 1697 cm^−1^ in the mixture of benzaldehyde and Ph_3_CCl in FT-IR spectrum. In ^1^H NMR, hydrogen in formyl group was deshielded and its chemical shift increased from 9.78 to 10.04 in the mixture of benzaldehyde and TrCl. In UV spectrum in n-hexane as a solvent, *λ*_max_ of absorption of the complex of benzaldehyde and trityl carbocation appeared at 245 nm which is different from benzaldehyde and Ph_3_CCl that observed at 240 and 222 nm, respectively.^8^

### The multi-component construction of 1-carbamatoalkyl-2-naphthols and 1-thioamidoalkyl-2-naphthols

**Scheme 10:**
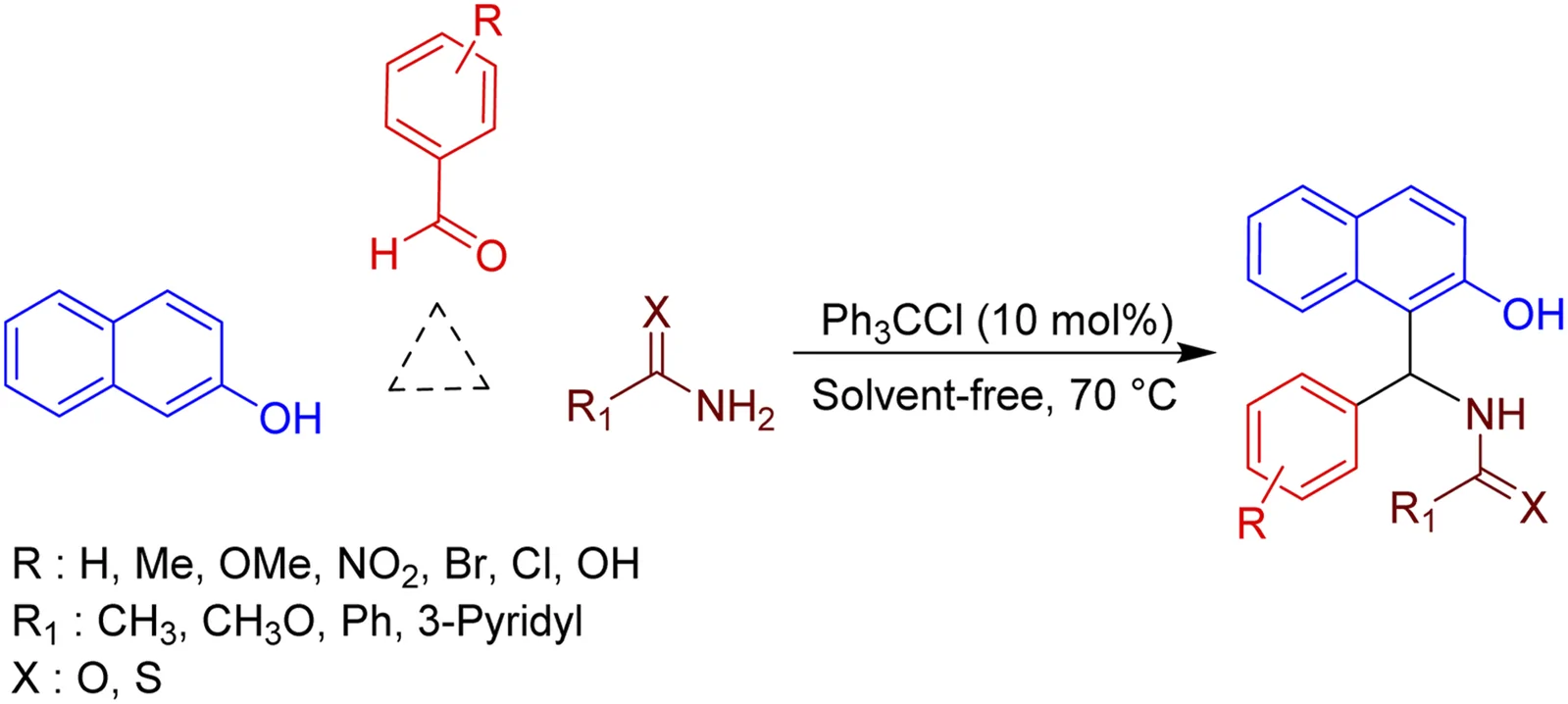
Synthesis of 1-amidoalkyl-2-naphthol, 1-carbamatoalkyl-2-naphthol, and 1-thioamidoalkyl-2-naphthol compounds using TrCl.

TrCl-catalyzed reactions between 2-naphthol and various aldehydes, performed under solvent-free conditions at 70 °C, have been extended to include acetamide, benzamide, thioacetamide, nicotinamide, and carbamates as alternatives to acetonitrile, affording 1-amidoalkyl-2-naphthols, 1-carbamatoalkyl-2-naphthols, and 1-thioamidoalkyl-2-naphthols in excellent yields and short reaction times (Scheme 10).^9^

**Scheme 11:**
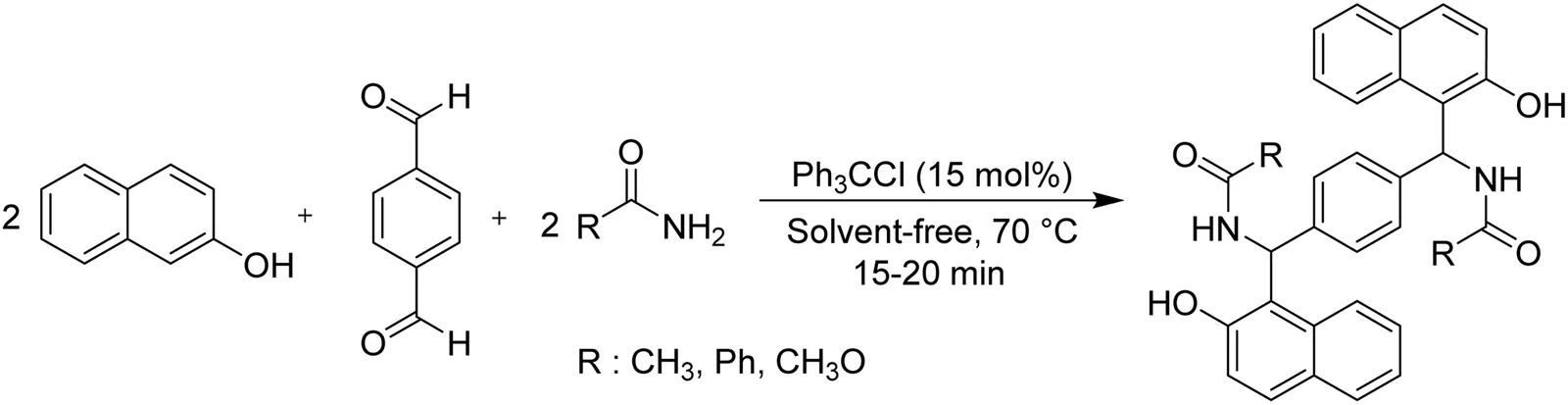
Synthesis of bis(1-amidoalkyl-2-naphthol) and bis(1-carbamatoalkyl-2-naphthol) in the presence of TrCl.

**Scheme 12:**
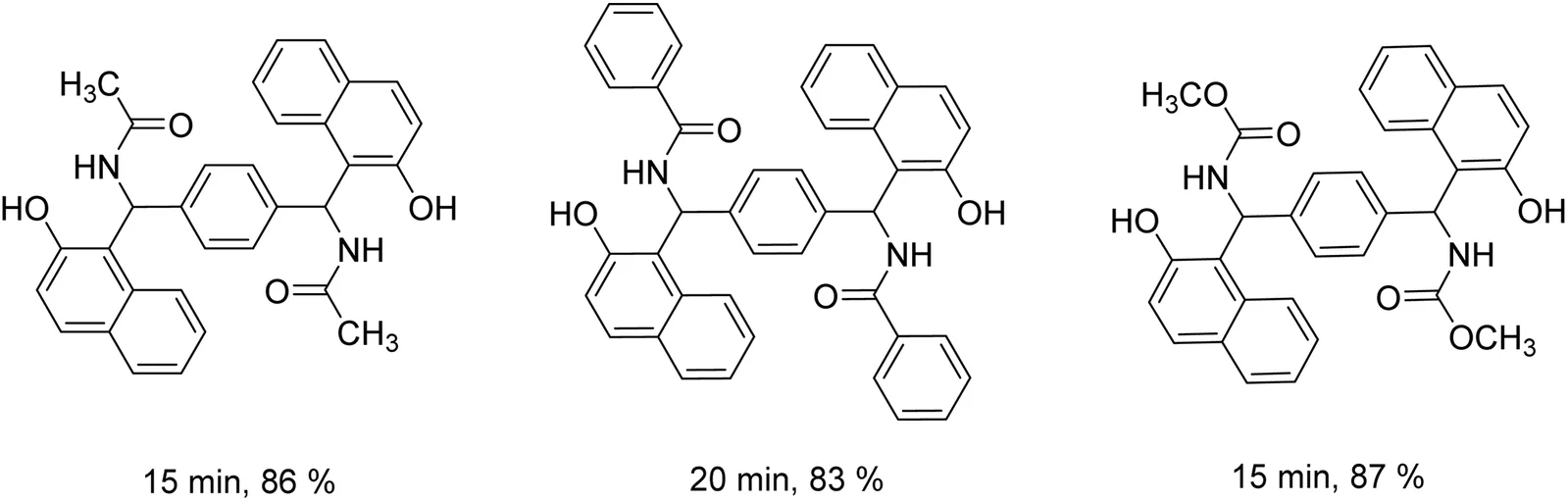
Products from the condensation of terephthalaldehyde, 2-naphthol and various amides in the presence of TrCl.

In addition to these compounds, bis(1-amidoalkyl-2-naphthol)s and bis(1-carbamatoalkyl-2-naphthol)s were also successfully prepared by the aforementioned method. For this purpose, 2 equivalents of 2-naphthol were reacted with 1 equivalent of terephthalaldehyde and 2 equivalents of amide in the presence of TrCl at 70 °C and under solvent-free condition (Schemes 11 and 12).^9^

### The three-component synthesis of amido alkylphenols

**Scheme 13:**
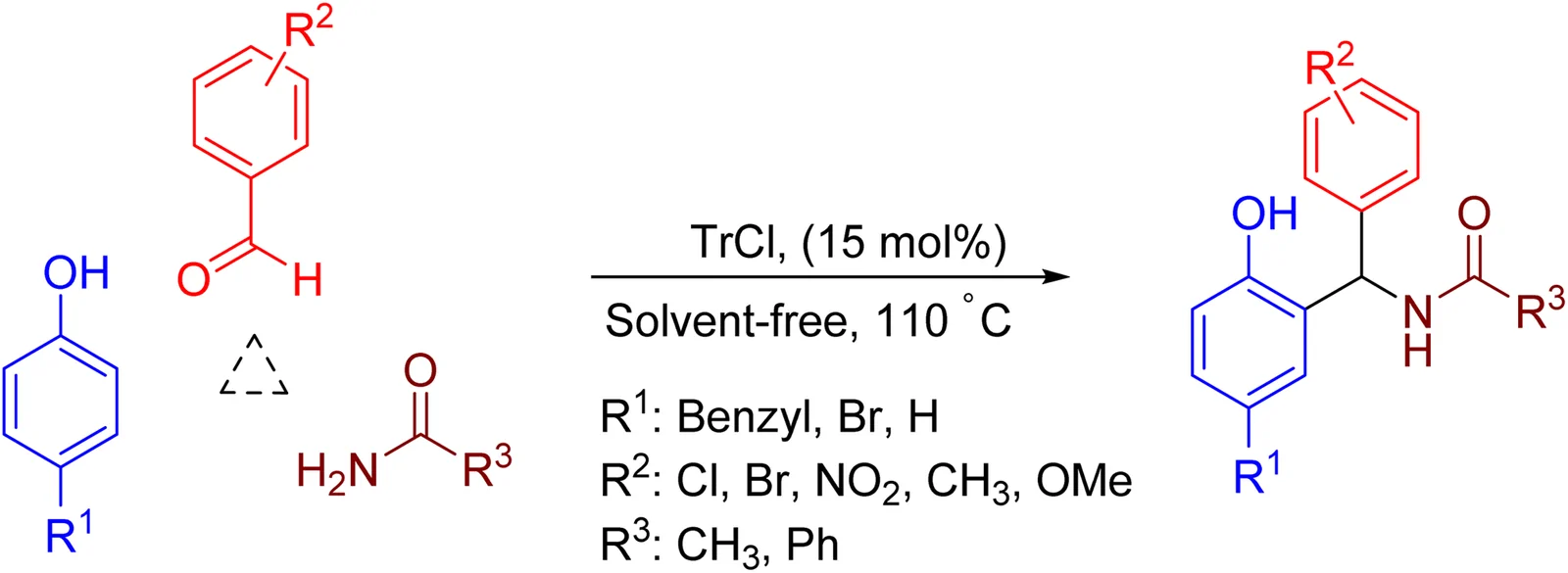
Production of amido alkylphenol derivatives in the presence of TrCl.

Also, the ternary condensation of phenol derivatives such as 4-bromophenol and 4-benzylphenol with aromatic aldehydes and amines such as acetamide and benzamide in the presence of catalytic amounts of TrCl under solvent-free condition at 110 °C was studied to produce amido alkylphenol derivatives (Scheme 13).^10^

**Scheme 14:**
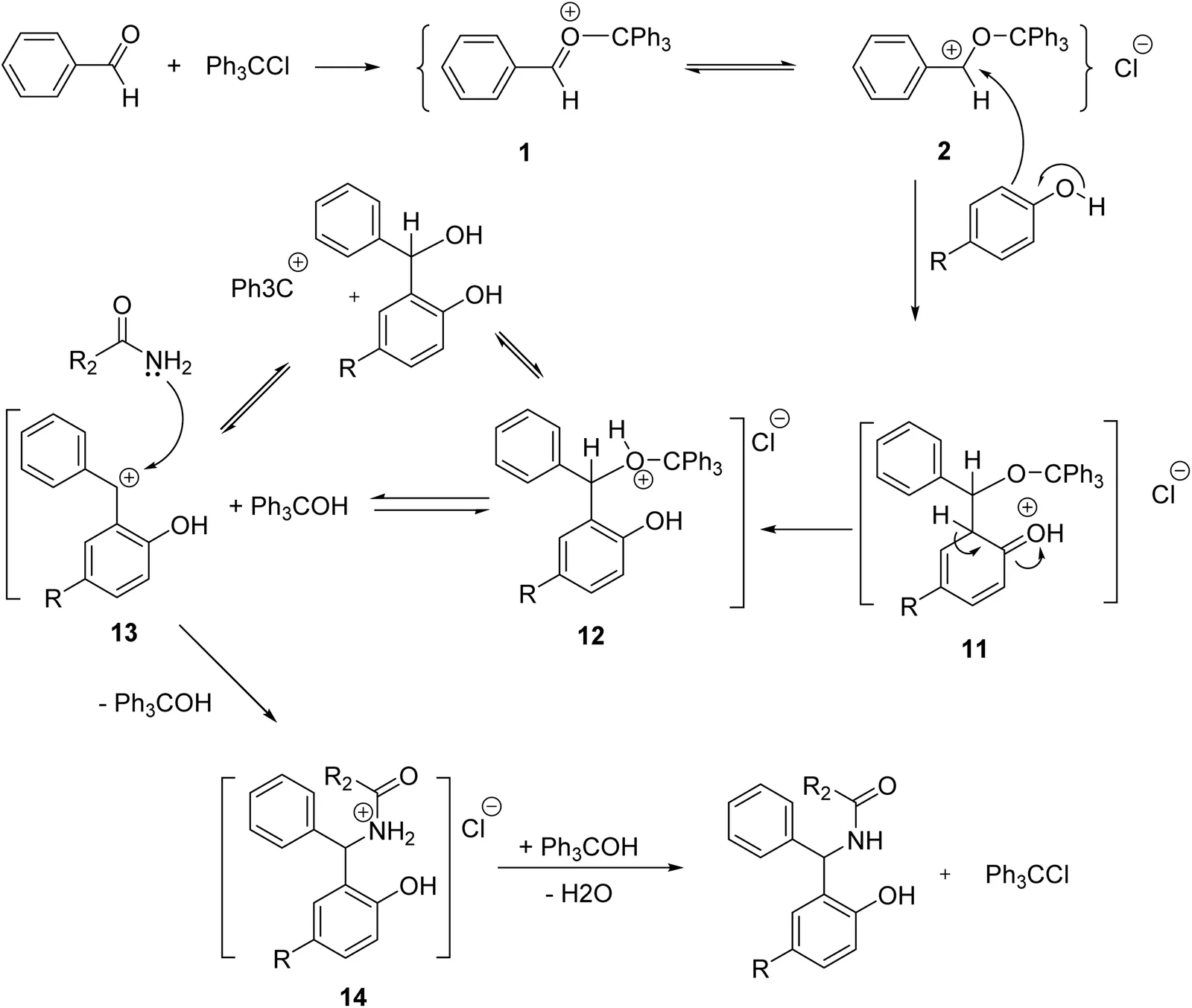
Mechanism of production of amido alkylphenol using trityl chloride.

In this reaction, the aryl aldehyde is first activated by the trityl carbocation to form two active aldehyde forms (1) and (2), which are converted into each other in a reversible reaction. Then, (1) and (2) react as an active intermediate with the phenolic compound to form intermediate (11), which is converted to intermediate (12) by proton transfer. In the next step (12), can be converted to intermediate (13) by removing a triaryl methanol molecule. After that, intermediate (12) or (13) reacts with an amide molecule to form intermediate (14) and a triaryl methanol molecule. Finally, intermediate (14) in the presence of triaryl methanol produces amidoalkylphenol by removing one molecule of water and regenerates TrCl (Scheme 14).^10^

### The three-component synthesis of tetrahydrobenzoxanthenes

**Scheme 15:**
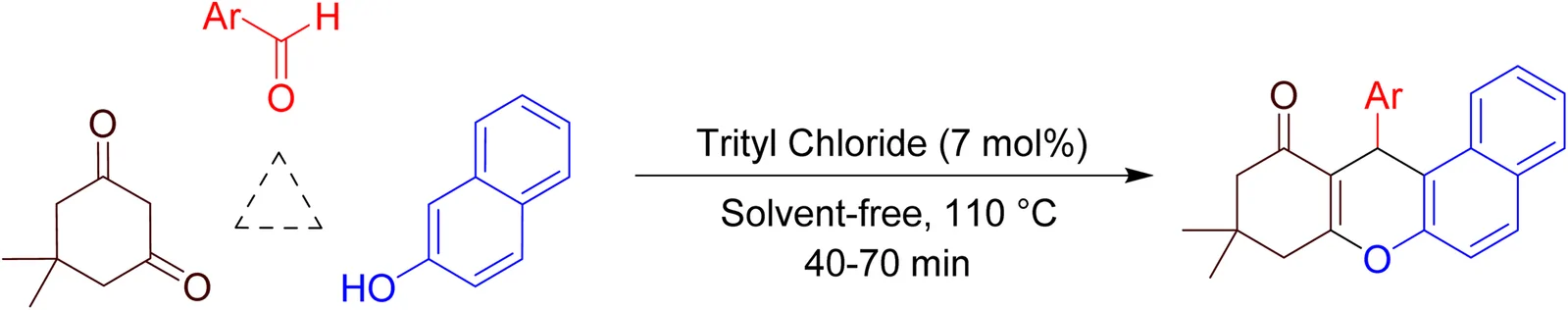
Production of tetrahydrobenzoxanthene compounds in the presence of TrCl.

The use of TrCl as an organic catalyst has been reported in the synthesis of tetrahydrobenzoxanthenes. In the aforementioned process, due to the *in situ* generation of carbocations in a neutral media, the three-component condensation of 2-naphthol, dimedone, and aromatic aldehydes was carried out using TrCl in solvent-free condition and at 70 °C, to give tetrahydrobenzoxanthene compounds (Scheme 15).^11^

**Scheme 16:**
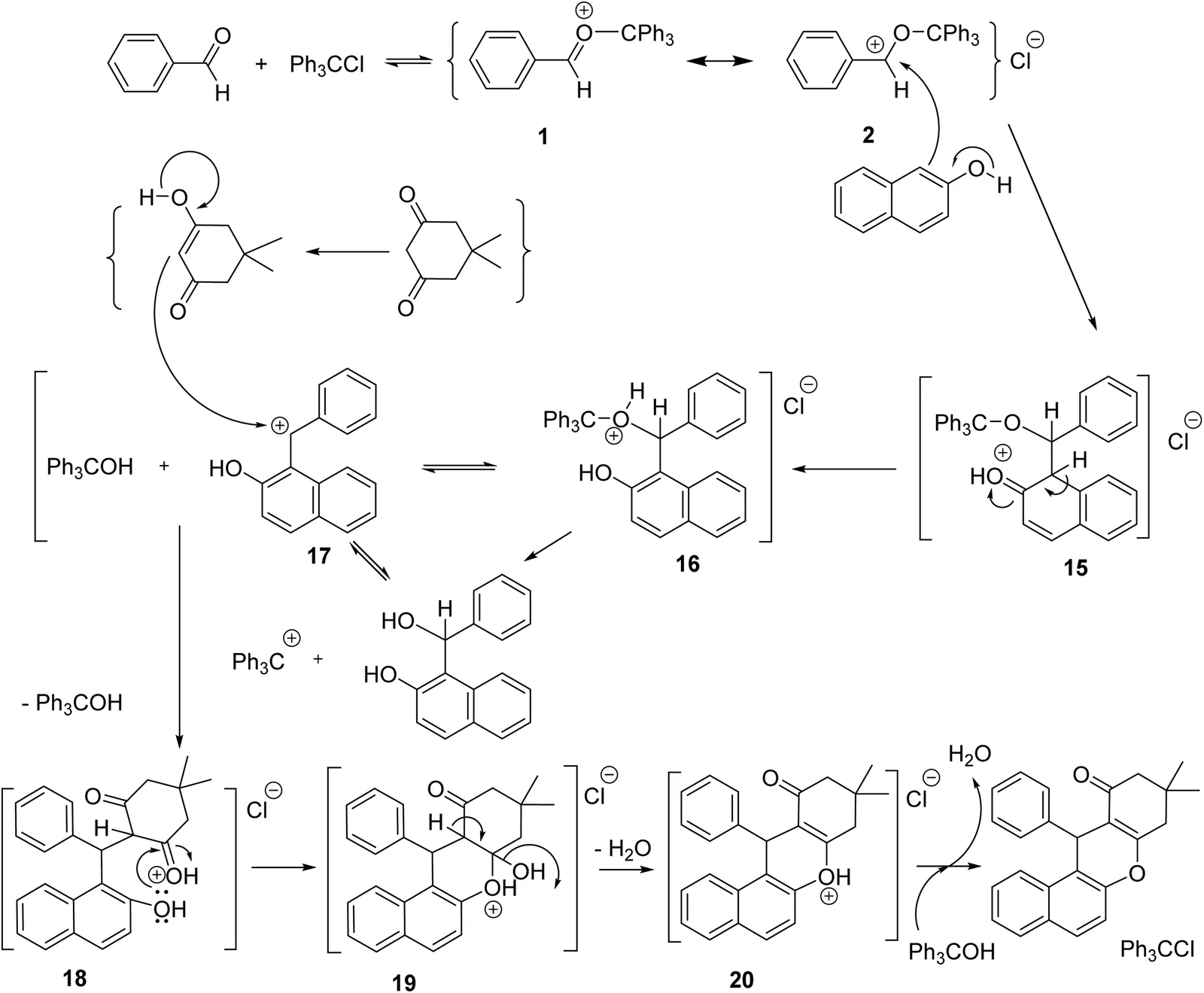
Mechanism of production of tetrahydrobenzoxanthene compounds in the presence of TrCl.

In the study of the reaction mechanism for the synthesis of tetrahydrobenzoxanthene compounds in the presence of TrCl as a catalyst, activated aldehyde forms (1) and (2) are produced by the interaction between aldehyde and TrCl. These complexes react as activated carbonyl groups with 2-naphthol and produce intermediate (15), which is converted to compound (16) by hydrogen transfer. Intermediate (16) is converted to (17) by the removal of one molecule of trityl alcohol. Then (17), reacts with dimedone to produce intermediate (18) and then, by intra molecular ring closing due to the reaction between the hydroxy group and the activated carbonyl group, intermediate (19) is produced. After that, by removing one molecule of water from intermediate (19), (20) is produced. Intermediate (20) in the presence of trityl alcohol produces the desired product by removing one molecule of water and regenerates trityl chloride. The produced TrCl in this way catalyzes the reaction until completion of the reaction (Scheme 16).^11^

TrCl has been compared with its derivatives, such as dimethoxytrityl chloride, monomethoxytrityl chloride, and trityl alcohol, in catalyzing the three-component condensation of dimedone, 2-naphthol, and benzaldehyde to afford tetrahydrobenzoxanthenes. In this investigation, trityl chloride showed the best catalytic performance to carry out the reaction with the short reaction time and the high yield of the desired product.^11^

### The pseudo three-component synthesis of dibenzoxanthene

**Scheme 17:**
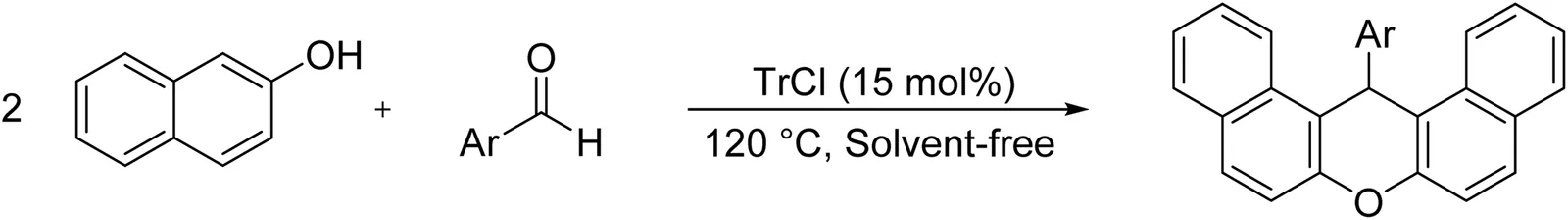
TrCl-mediated fabrication of dibenzoxanthenes.

Dibenzoxanthene compounds have been prepared through a solvent-free, pseudo multicomponent cyclization of two equivalents of 2-naphthol and one equivalent of an aldehyde catalyzed by TrCl at 120 °C (Scheme 17).^12^

**Scheme 18:**
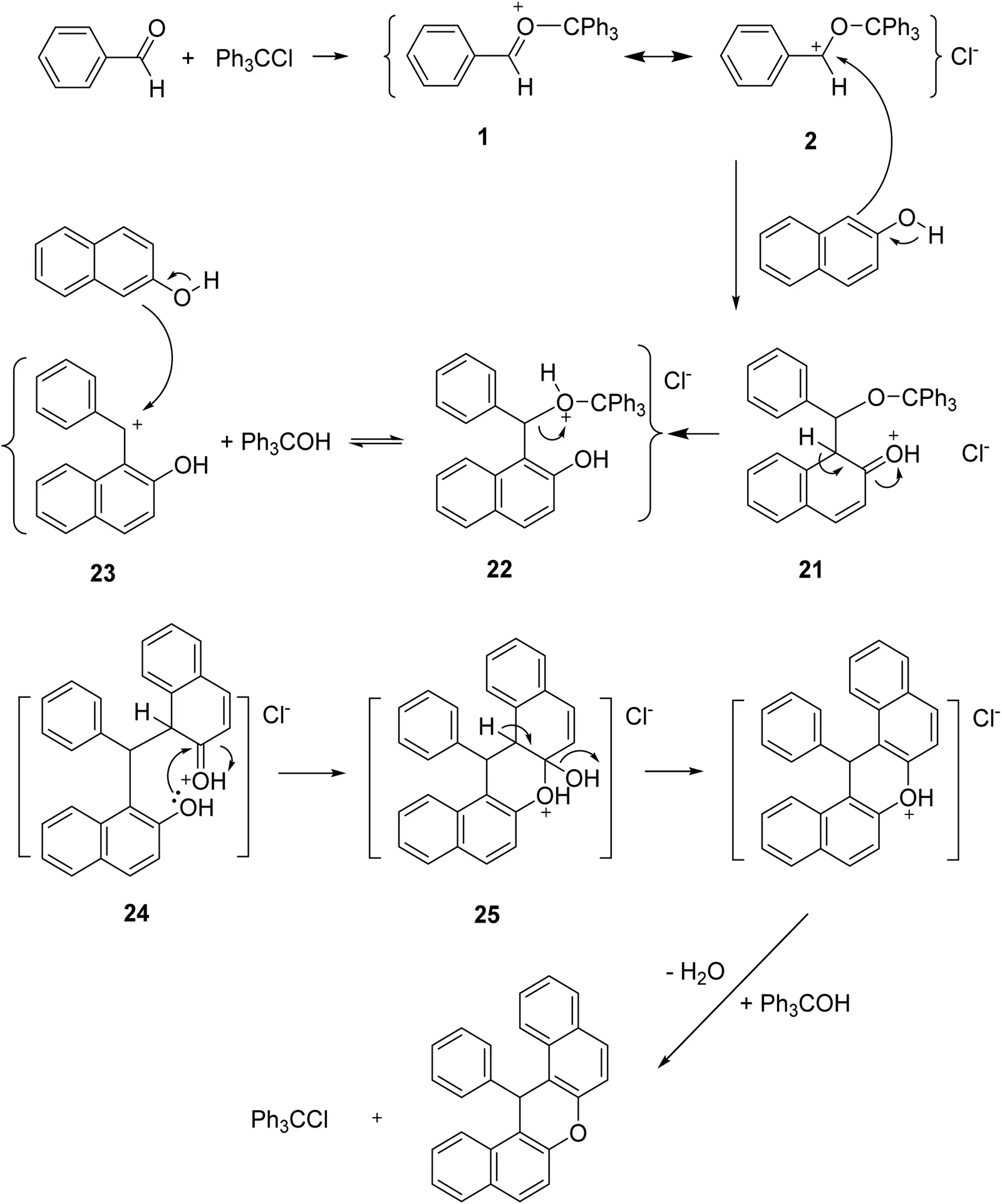
Proposed mechanism for the TrCl-catalyzed construction of dibenzoxanthenes.

In this reaction, the addition of the first molecule of 2-naphthol to the aldehyde activated by the trityl carbocation forms intermediates (1) and (2) to give intermediate (21) which converted to (22) after tautomerization and also (23) after removing of one molecules triphenylmethanol. In the next step, the second molecule of 2-naphthol reacts with intermediate (23) to prepare (24) and, through intramolecular nucleophilic attack and ring closing of (24) with the elimination of one molecule of water in (25), the dibenzoxanthene product is produced, and TrCl is regenerated (Scheme 18).^12^

### Pseudo four-component approach to the production of 2,4,6-triarylpyridines

**Scheme 19:**
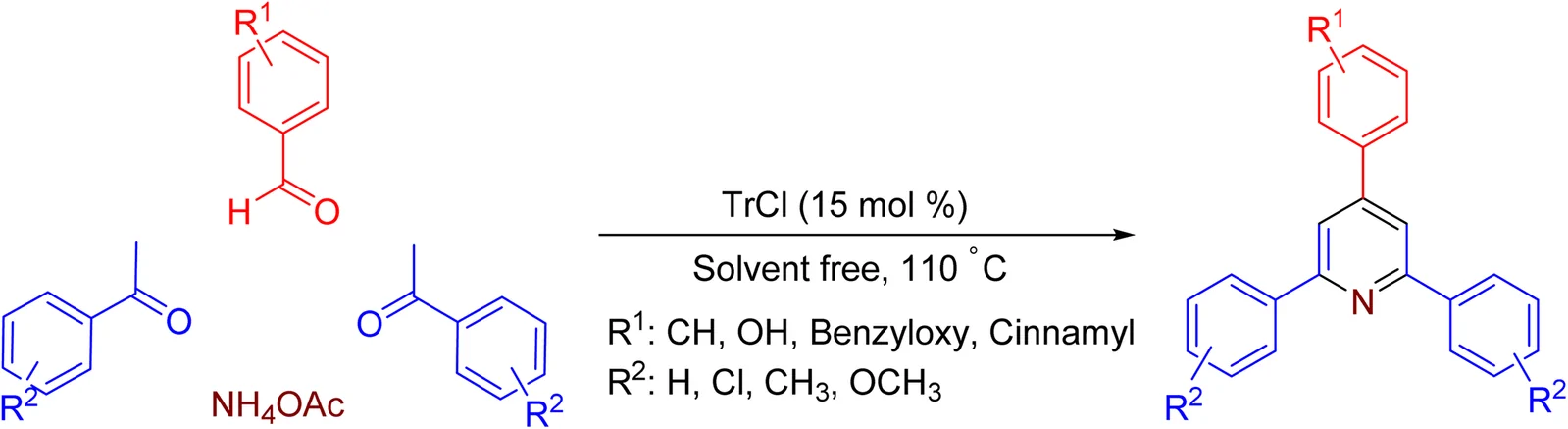
Construction of 2,4,6-triarylpyridines utilizing TrCl.

The formation of 2,4,6-triarylpyridines represents another transformation catalyzed by TrCl. In this pseudo-four-component process, aromatic aldehyde (1 equiv.) undergoes condensation with acetophenone (2 equiv.) and ammonium acetate (1 equiv.) in the presence of catalytic amounts of TrCl. The reaction proceeds at 110 °C under solvent-free condition (Scheme 19).^13^

**Scheme 20:**
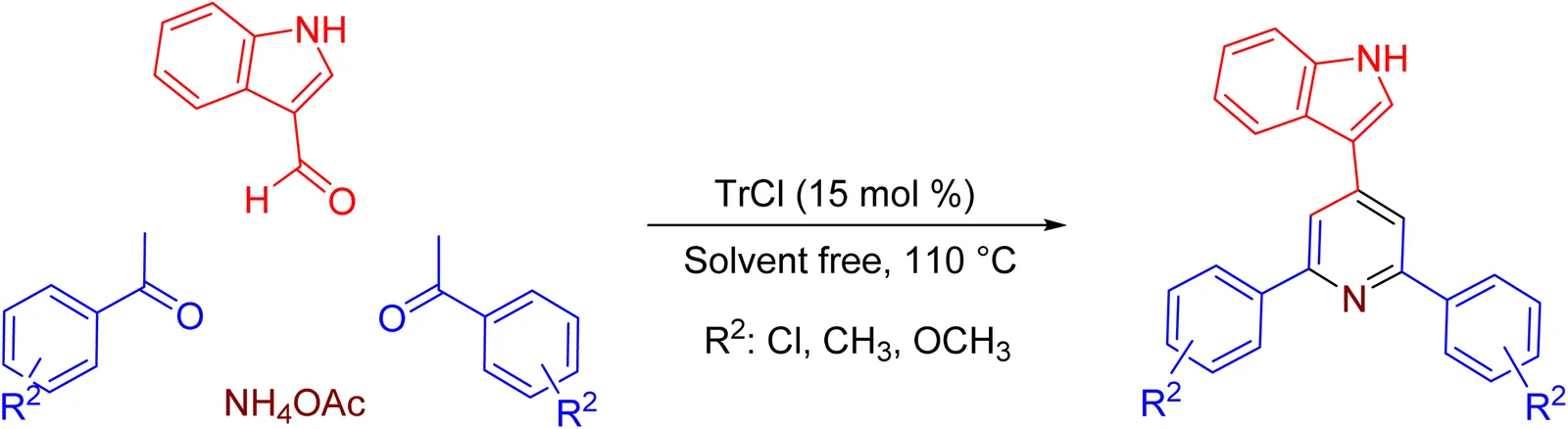
The condensation of indole-3-carbaldehyde, acetophenone and ammonium acetate utilizing TrCl.

A pseudo-four-component condensation reaction involving indole-3-carbaldehyde, acetophenone, and ammonium acetate has also been conducted utilizing TrCl (Scheme 20).^13^

**Scheme 21:**
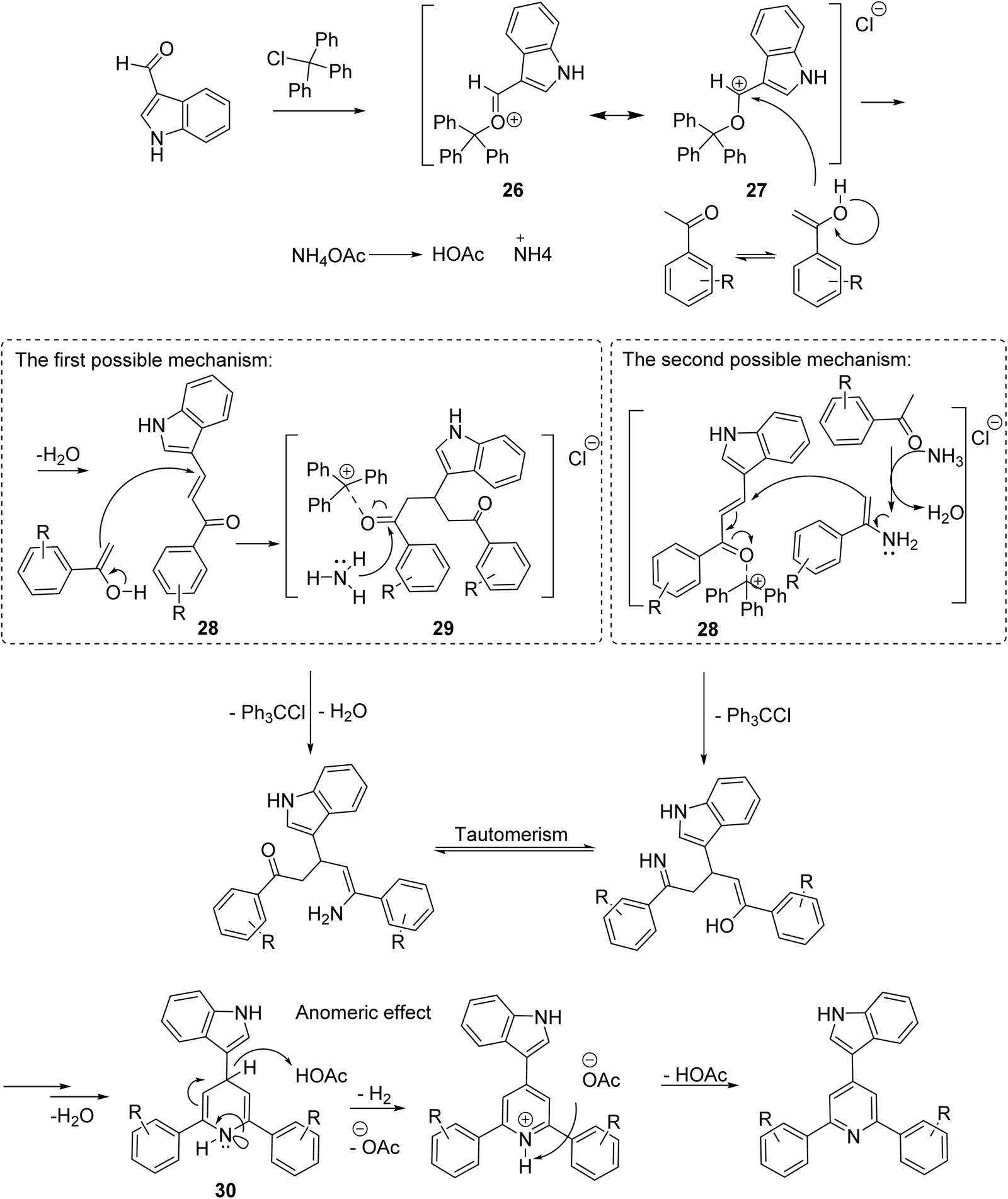
Mechanism of fabrication of 2,4,6-triarylpyridines utilizing TrCl.

In the mechanism of this reaction, at first the aromatic aldehyde is activated by the trityl carbocation to produce intermediates (26) and (27) which are interconverted in a reversible reaction. The activated aldehyde undergoes reaction with acetophenone in its enol form, leading to the formation of chalcone (28). A second molecule of acetophenone, in its enol form, subsequently attacks to the chalcone as a Michael acceptor to provide 1,5-dicarbonyl (29) after tautomerization. Ammonia, which is produced in the reaction medium from ammonium acetate, reacts with 1,5-dicarbonyl to form 1,4-dihydropyridine (30) which is readily hydrogenated to produce 2,4-diarylpyridine indoles. In another proposed mechanism, an enamine molecule may be produced from the reaction of acetophenone with ammonia, and then in the next step after the reaction of enamine with chalcone, the desired product is produced.^13^ According to previous reports, the oxidation of 1,4-dihydropyridine compounds and similar nitrogen-containing heterocycles can be performed by anomeric-based oxidation process.^14–16^ In this process, acetic acid produced from ammonium acetate salt in the reaction medium interacts with the hydride anion separated by the anomeric effect in intermediate (30) to produce one molecule of hydrogen gas and acetate anion. Finally, the acetate anion removes a proton from the prepared pyridinium ion, reproducing acetic acid and the final product (Scheme 21).

### The four-component synthesis of pyranopyrazole

**Scheme 22:**
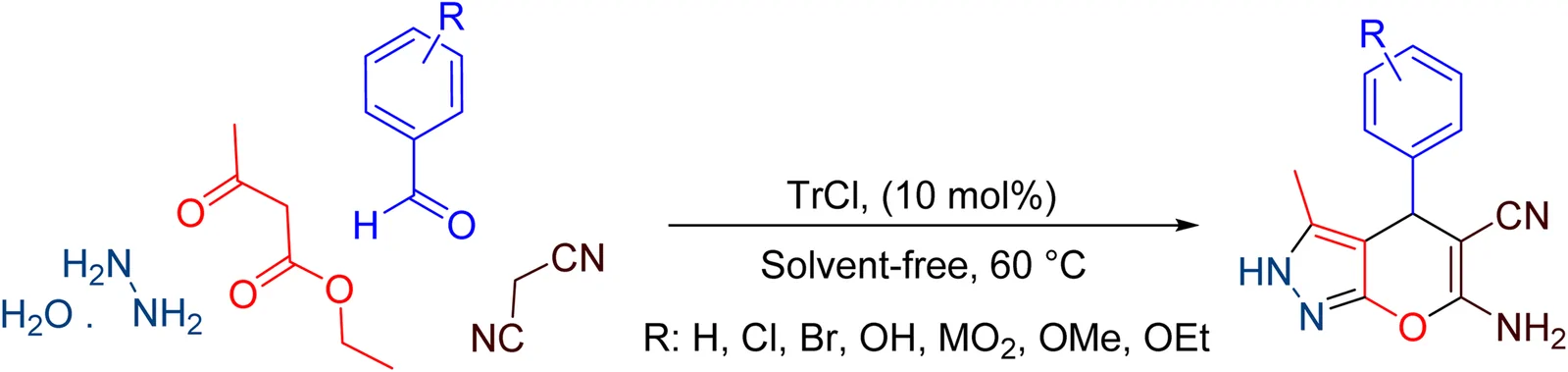
Synthesis of pyranopyrazole compounds utilizing TrCl.

Another transformation catalyzed by TrCl involves the production of pyranopyrazole derivatives. These compounds have been obtained through a solvent-free four-component condensation of an aldehyde, malononitrile, ethyl acetoacetate and hydrazine hydrate at 60 °C utilizing TrCl (Scheme 22).^17^

**Scheme 23:**
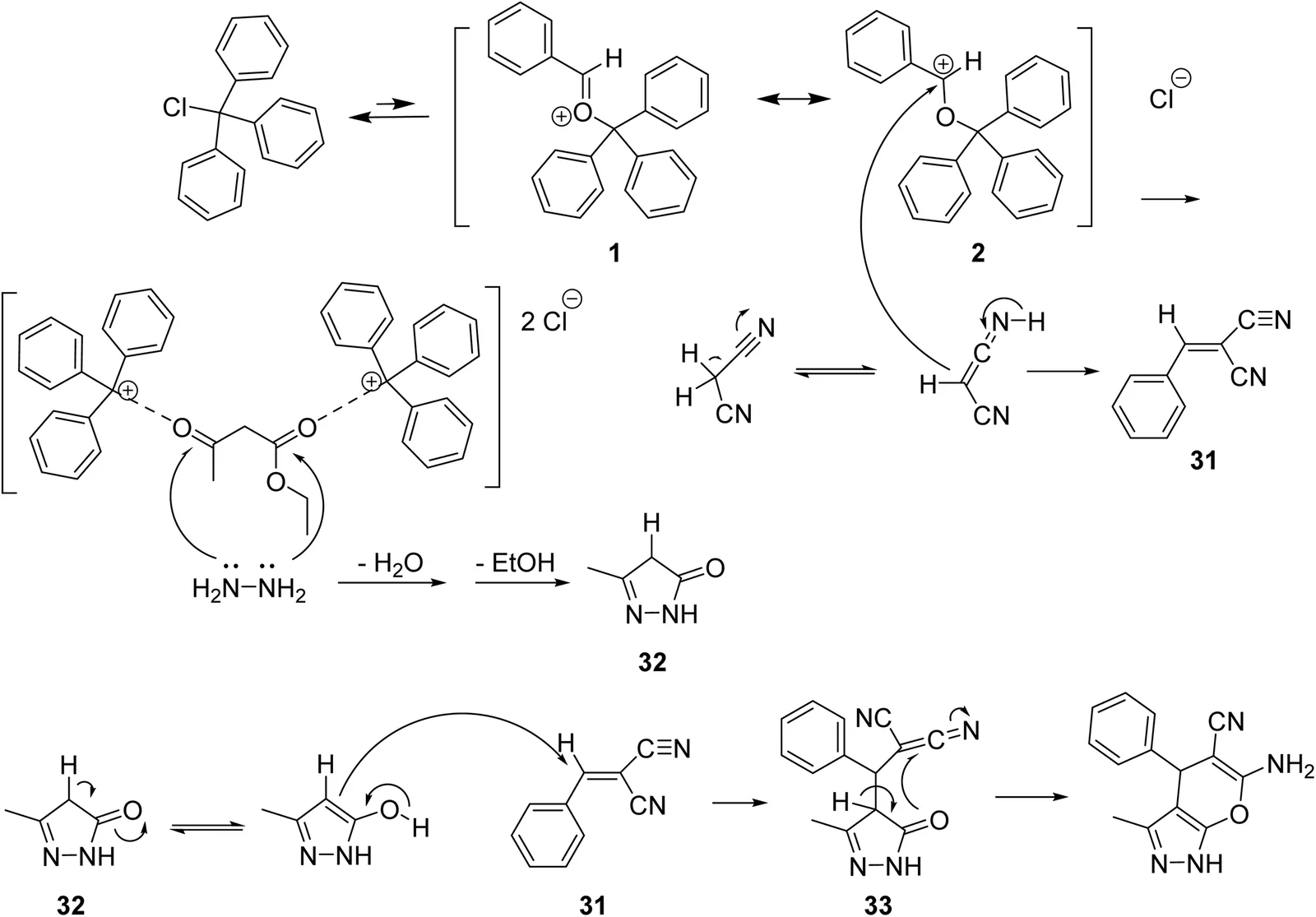
Mechanism of synthesis of pyranopyrazole compounds using TrCl.

In the proposed mechanism of this reaction, first the aryl aldehyde is activated by the trityl carbocation, producing the active intermediates (1) and (2). The reaction of malononitrile with the active intermediates (1) and (2) produces the cyanoolefin derivative (31). In the next step, ethyl acetoacetate is activated by the trityl carbocation, and hydrazine attacks to carbonyl group of the activated ethyl acetoacetate. Then, an intramolecular nucleophilic attack *via* the other NH_2_ group of hydrazine on the other carbonyl group of ethyl acetoacetate produces the 3-methylpyrazolone compound (32). Lastly, Michael reaction of 3-methylpyrazolone (32) with the cyanoolefin compound (31) affords intermediate (33), which is converted to the desired pyranopyrazole product by intramolecular cyclization (Scheme 23).^17^

### The four-component fabrication of tetrasubstituted imidazoles

**Scheme 24:**
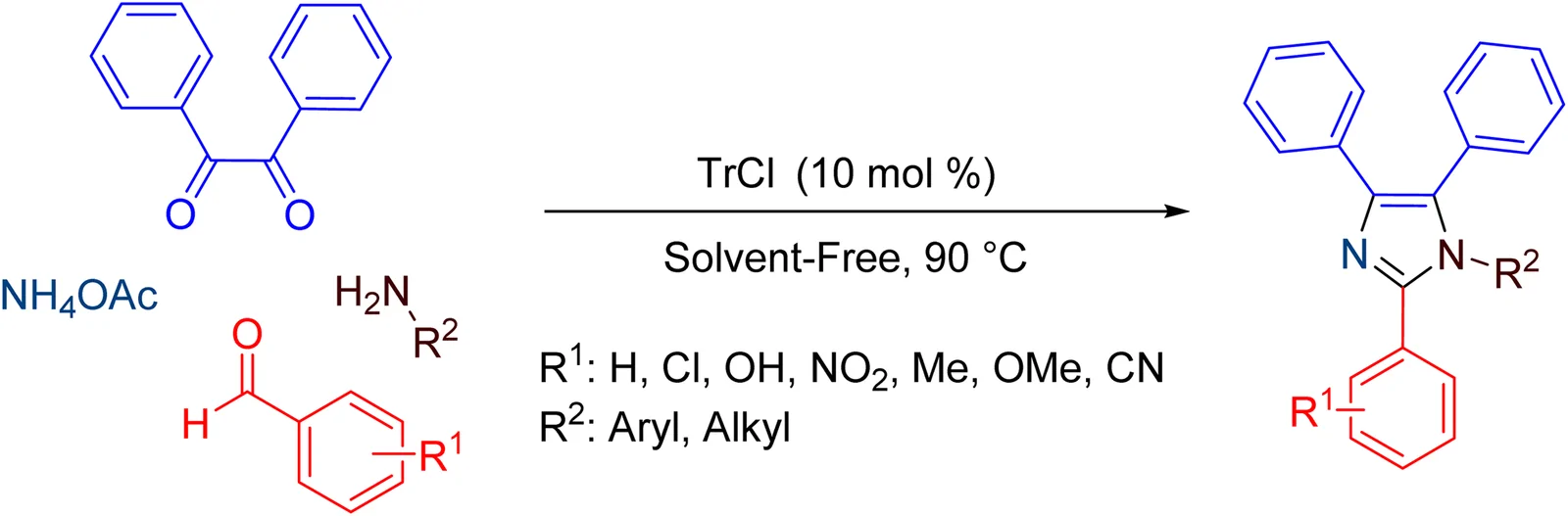
Construction of tetrasubstituted imidazoles using TrCl.

The four-component condensation reaction of benzil, aldehyde, primary amine and ammonium acetate using catalytic amounts of TrCl at 90 °C under solvent-free conditions has been carried out to produce tetrasubstituted imidazoles. In this work, the reaction proceeded efficiently, giving the desired products in high yields within short reaction times (Scheme 24).^18^

**Fig. 1:**
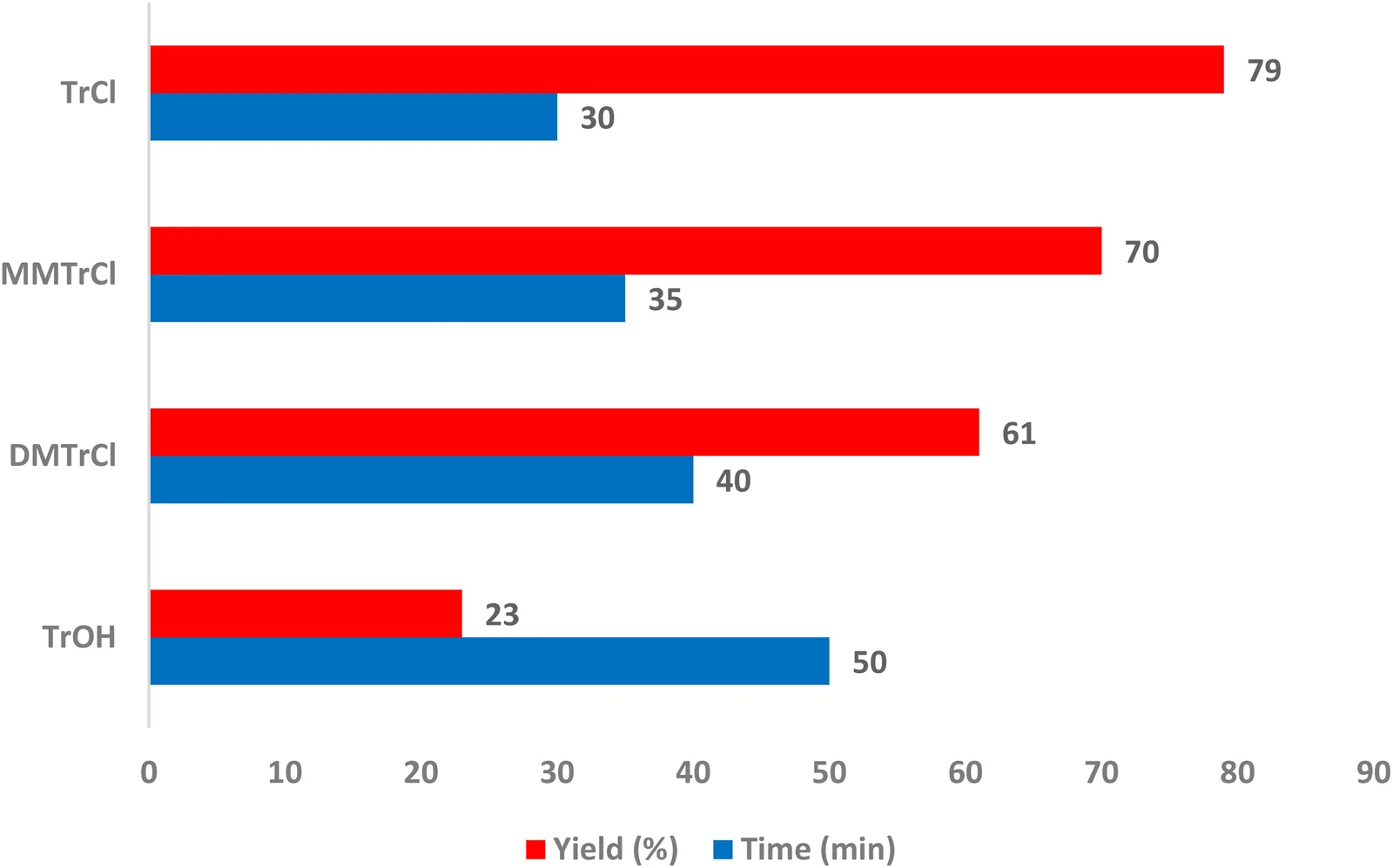
Comparative study of the catalytic performance of trityl derivatives in the production of tetrasubstituted imidazoles.

The condensation of benzyl with benzaldehyde, aniline and ammonium acetate in the presence of catalytic amounts of other trityl derivatives including trityl alcohol, MMTrCl and DMTCl was investigated in comparison with TrCl. In this investigation, TrCl exhibited the highest catalytic efficiency along with the shortest reaction time.^18^ The times and efficiencies of the mentioned catalysts are shown in Fig. 1.

The turnover number (TON) and turnover frequency (TOF) values calculated for trityl derivatives in comparison with each other (Table 1). According to the values ​​obtained from this Table 1, TrCl has a higher TON and TOF than other catalysts and it has better efficiency for catalytic application on the fabrication of tetrasubstituted imidazoles.^18^

**Table 1:** Turn over number and turn over frequency values of different catalysts in the synthesis of tetrasubstituted imidazoles

Entry	Catalyst (10 mol%)	TOF (min^−1^)	TON
1	TrOH	0.046	2.3
2	DMTrCl	0.152	6.1
3	MMTrCl	0.200	7
4	TrCl	0.263	7.9

**Scheme 25:**
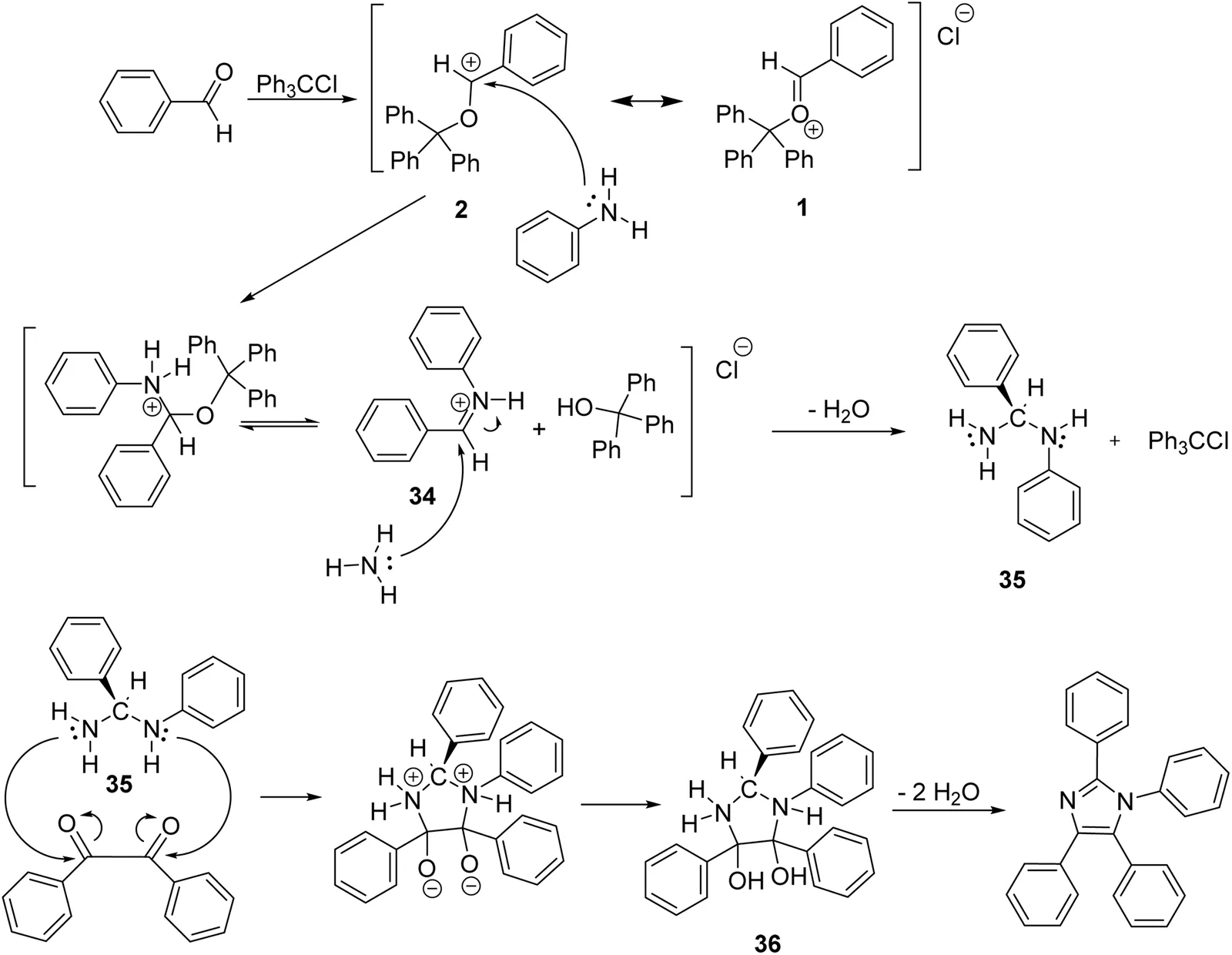
Mechanism of synthesis of tetrasubstituted imidazoles using TrCl.

In the fabrication of tetrasubstituted imidazoles, the aldehyde is activated by the trityl carbocation produced from the dissociation of TrCl to form two activated aldehyde forms (1) and (2). The activated aldehyde reacts with the amine to form the iminium intermediate (34). Then, intermediate (34) reacts with ammonia from the dissociation of ammonium acetate to form intermediate (35), by eliminating one molecule of water and regenerating trityl chloride. In the next stage, intermediate (36) is produced by nucleophilic attack of amino group in (35) on the carbonyl groups in benzil structure. Finally, the desired tetrasubstituted imidazole is produced by eliminating two molecules of water from intermediate (36) (Scheme 25).^18^

### The three-component synthesis of pyrimido[4,5-*b*]quinolines

**Scheme 26:**
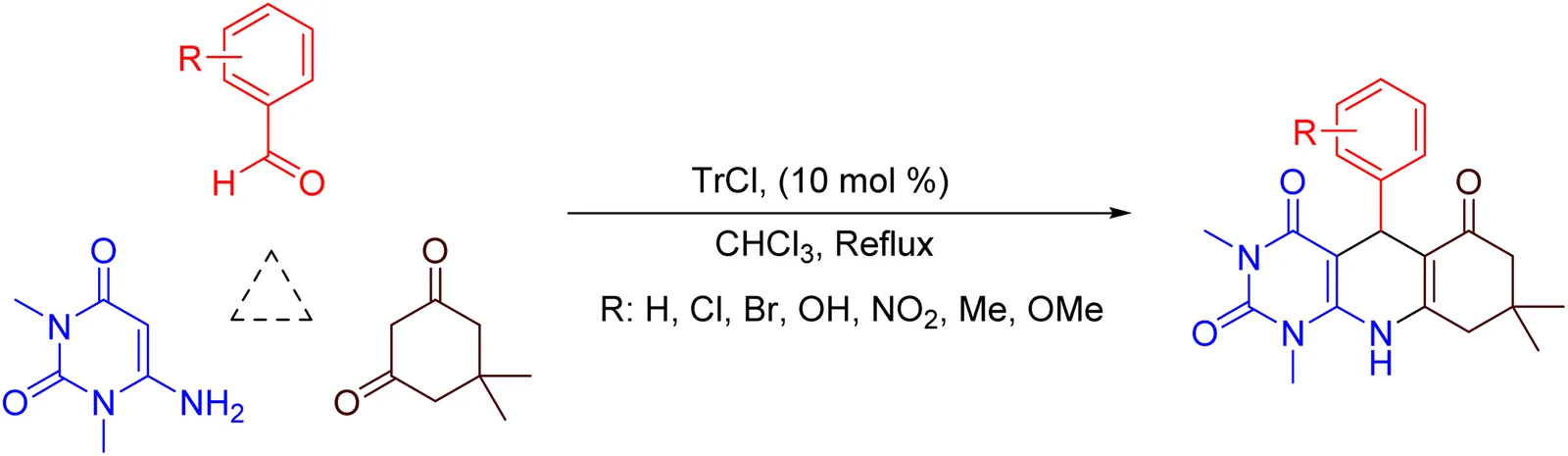
TrCl-mediated synthesis of pyrimido[4,5-*b*]quinolines.

The synthesis of pyrimido[4,5-*b*]quinoline derivatives has been investigated *via* a three-component condensation of aromatic aldehydes, 6-amino-1,3-dimethyluracil and dimedone, using catalytic amounts of TrCl in chloroform under reflux conditions (Scheme 26).^19^

**Scheme 27:**
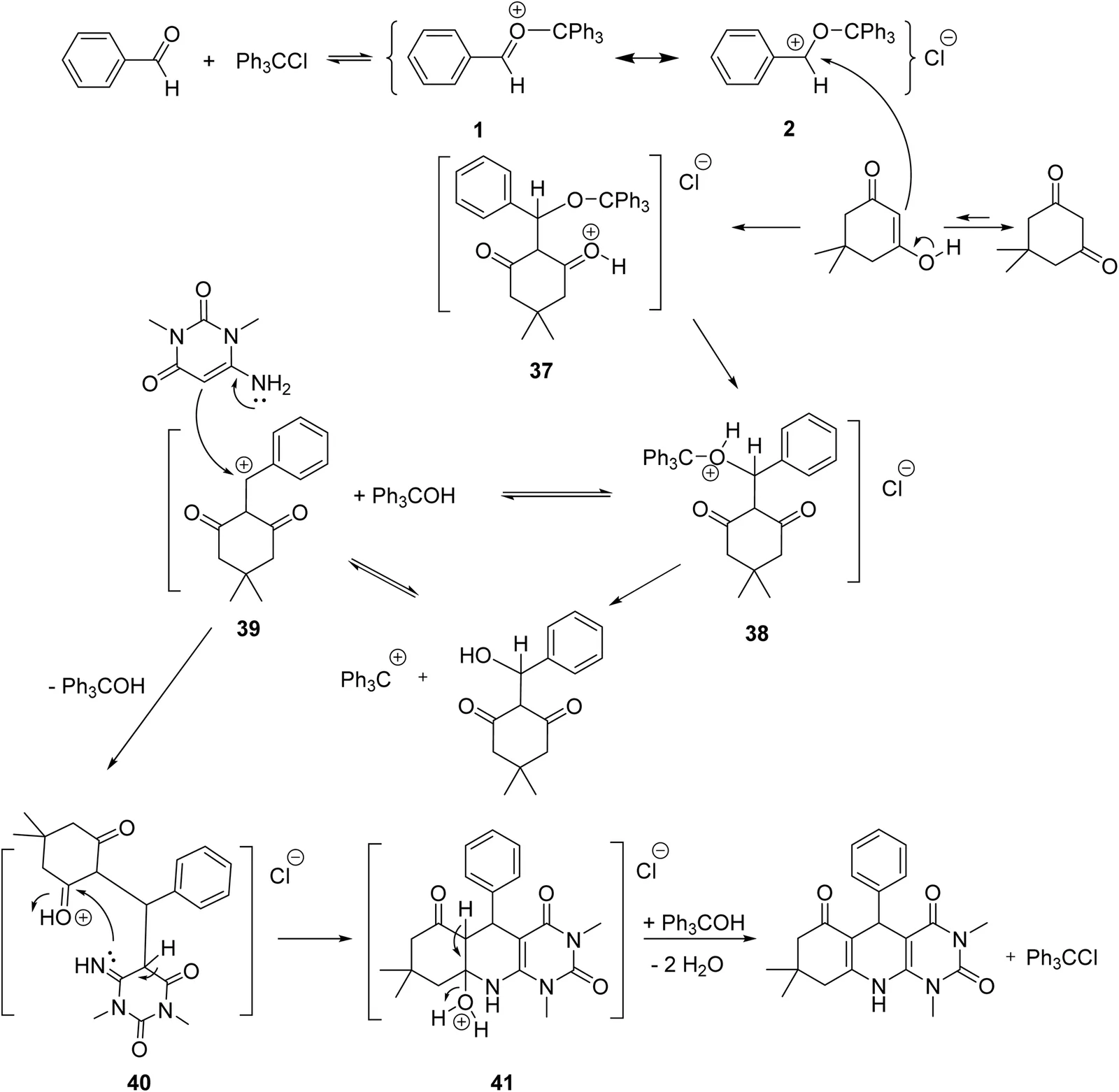
Mechanism pathway for the TrCl-catalyzed fabrication of pyrimido[4,5-*b*]quinolines.

In this reaction, dimedone first tautomerizes to the enol form and reacts with the activated aldehyde forms (1) and (2) by the trityl carbocation to produce intermediate (37), which can be converted to intermediates (38) and (39). In the next step, 6-amino-1,3-dimethyluracil attacks intermediate (39) to produce (40), which undergoes an intramolecular nucleophilic attack after the elimination of one molecule of water the expected product is produced. TrCl is regenerated by the reaction of trityl alcohol with intermediate (41) and the elimination of one molecule of water (Scheme 27).^19^

### The pseudo three-component construction of bis(arylidene)cycloalkanones

**Scheme 28:**
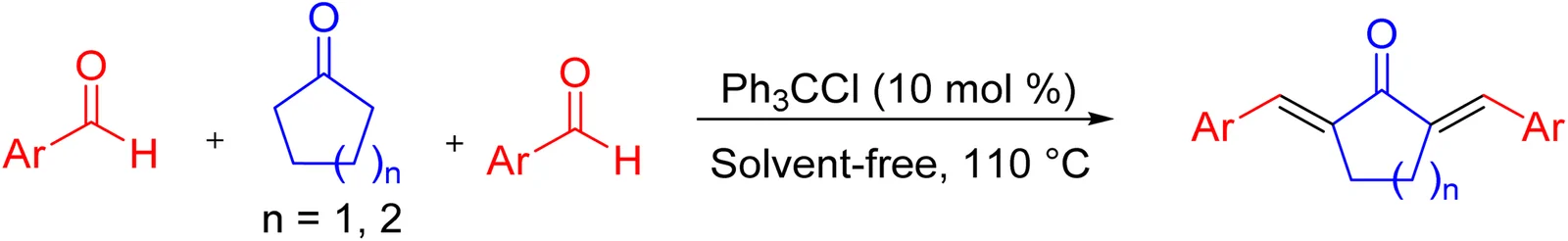
Aldol cross-condensation process utilizing TrCl.

TrCl as a catalyst, by producing trityl carbocation as a mild Lewis acid in the reaction medium, catalyzes the aldol cross-condensation process between one mole of cycloalkanone including of cyclopentanone, cyclohexanone and cycloheptanone with two moles of arylaldehyde at 110 °C under solvent-free condition with suitable yields and relatively good time (Scheme 28).^20^

**Scheme 29:**
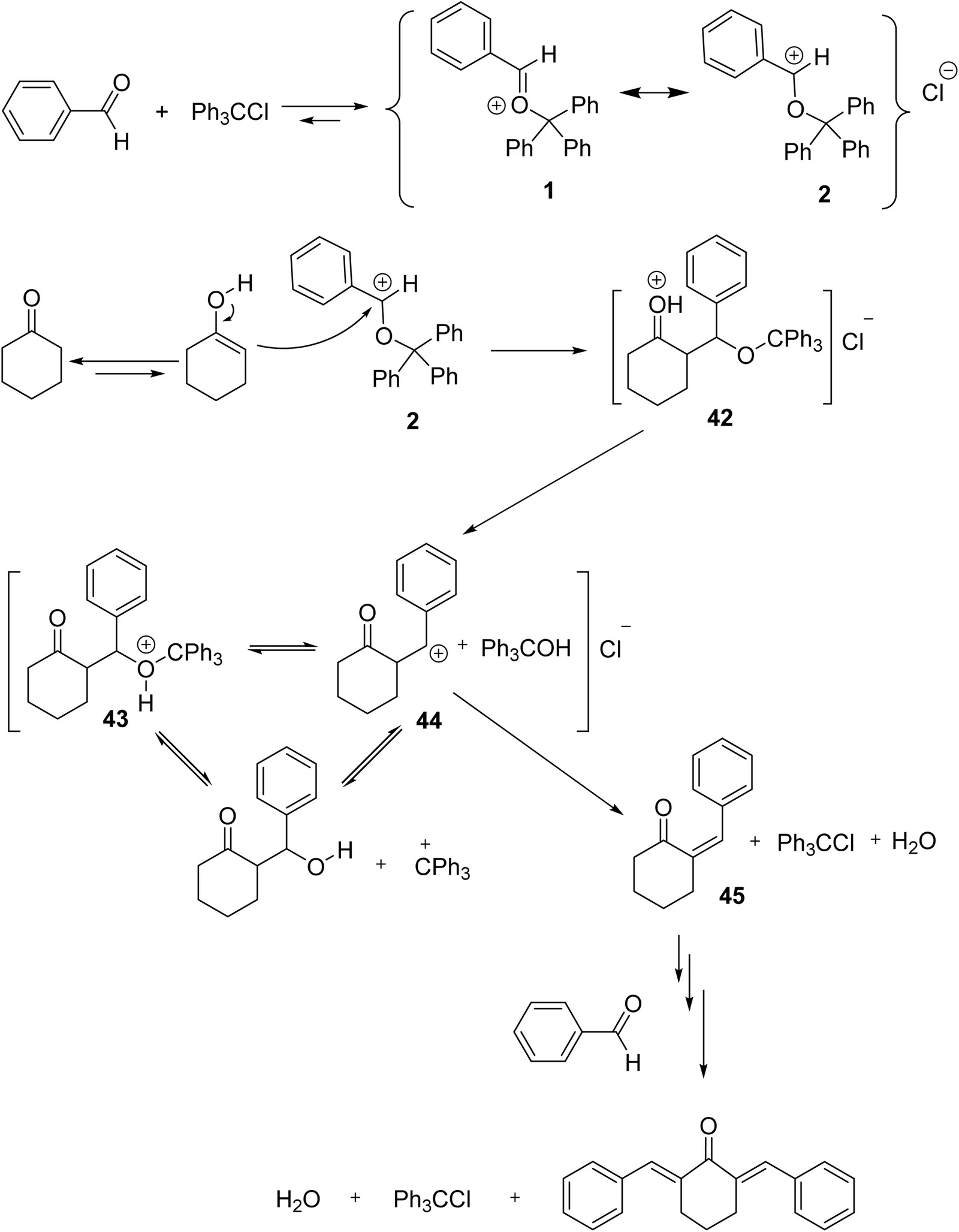
Mechanism of the aldol cross-condensation process using TrCl.

In this reaction, the active forms (1) and (2) are first produced in the reaction medium by the interaction of aldehyde with trityl chloride. Then, cycloalkanone reacts with the activated forms of aldehyde and produces intermediate (42). From the changes in the structure of intermediate (42), intermediates (43) and (44) are produced along with trityl alcohol. From the reaction of intermediate (44) with trityl alcohol, compound (45) and TrCl are produced by removing one molecule of water. From the reaction of compound (45) and another molecule of aldehyde in the presence of trityl chloride, a bis(arylidene)cycloalkanone product is produced by a mechanism similar to the next step, and by removing of one molecule of water, TrCl is regenerated in the reaction medium (Scheme 29).^20^

### The pseudo three-component synthesis of alkylidene bisamides

**Scheme 30:**
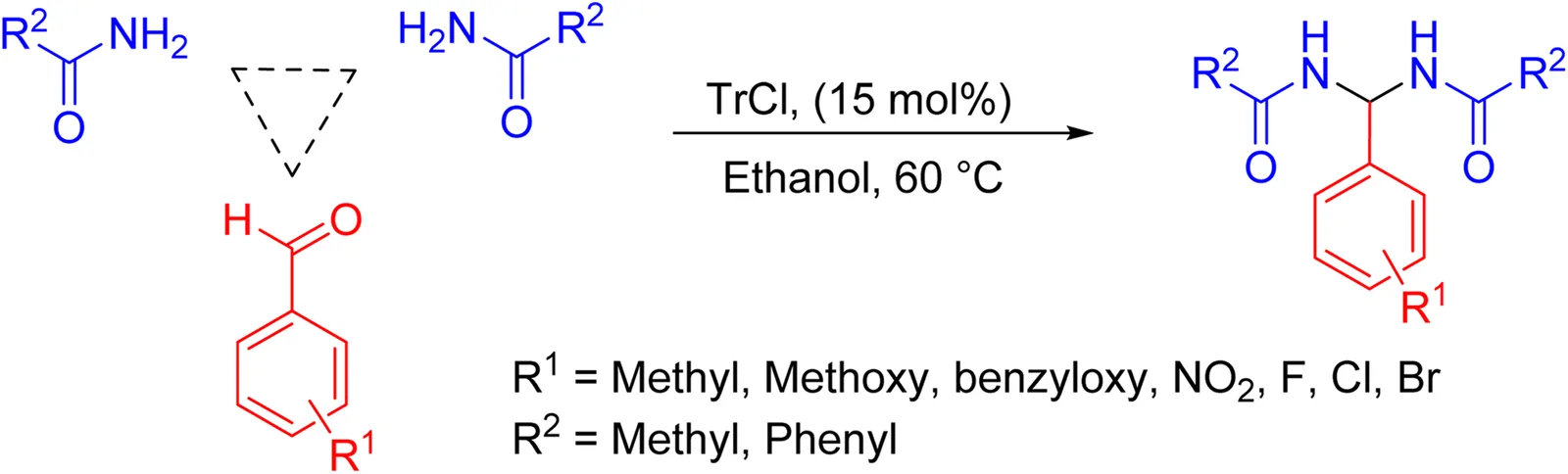
Fabrication of alkylidene bisamides using TrCl.

A mild and efficient route to alkylidene bisamide derivatives has been developed employing trityl chloride as a homogeneous catalyst. In this protocol, one equivalent of aldehydes reacts with two equivalents of amides in ethanol at 60 °C under catalytic activation by TrCl. The catalytic role is attributed to the *in situ* formation of a trityl carbocation from trityl chloride, which enhances the electrophilicity of the aldehyde in the reaction medium. This activation facilitates nucleophilic addition of amides such as acetamide and benzamide, ultimately yielding the corresponding alkylidene bisamide products (Scheme 30).^21^

**Scheme 31:**
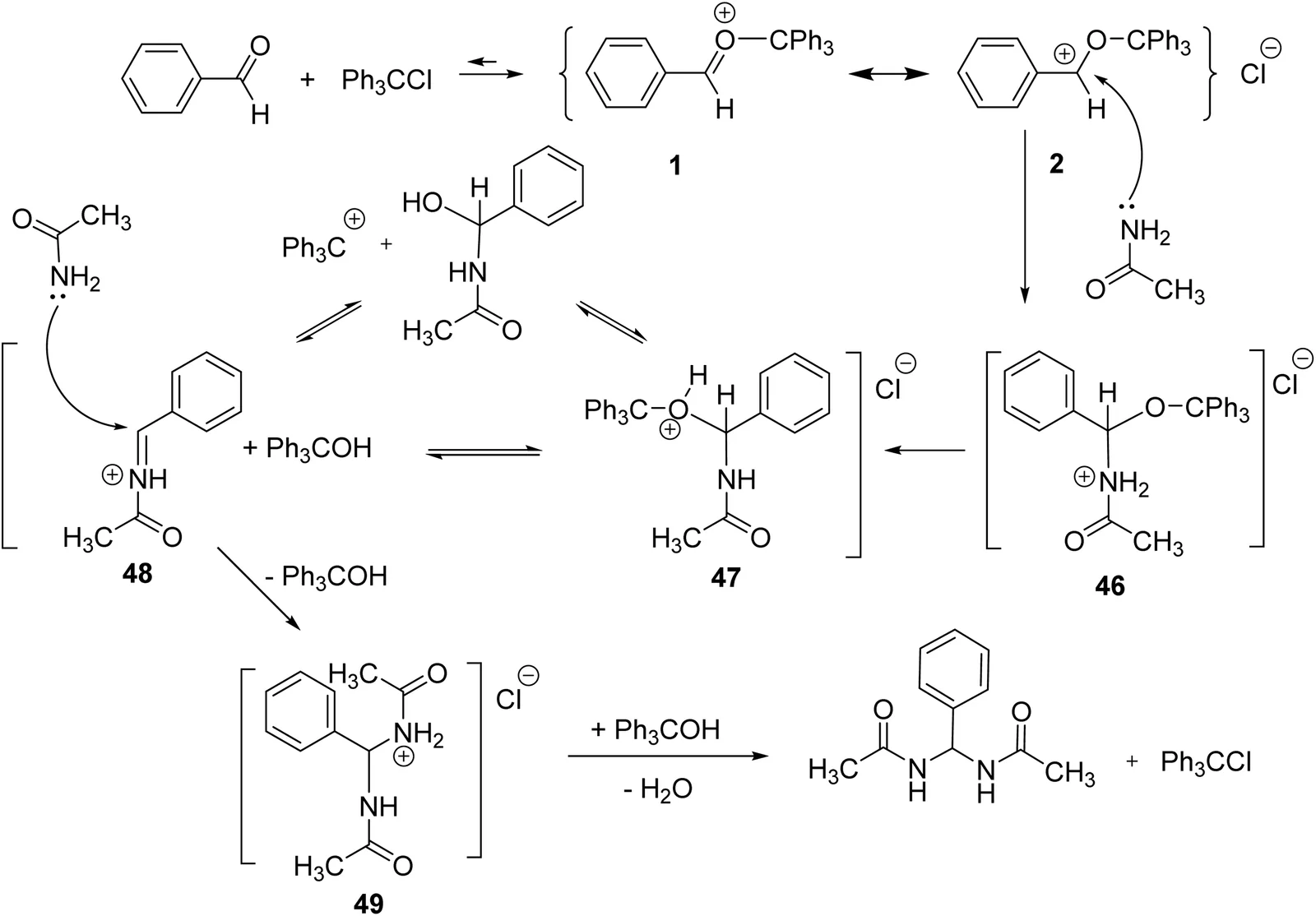
Mechanism of production of alkylidene bisamides using TrCl.

As in the previous reactions, the amide acts as a nucleophile and attacks the activated aldehyde carbonyl group by trityl carbocation (1 and 2) to produce intermediate (46), which is converted to (47) by proton transfer. Intermediate (47) is converted to (48) by loss of one molecule of trityl alcohol. Then, another amide molecule acts as a nucleophile and attacks to intermediate (48) to produce (49). Intermediate (49) in the presence of trityl alcohol produces the desired product by elimination of one molecule of water and TrCl is regenerated in this step (Scheme 31).^21^

### The pseudo three-component synthesis of biscoumarins and dibenzo xanthene tetraones

**Scheme 32:**
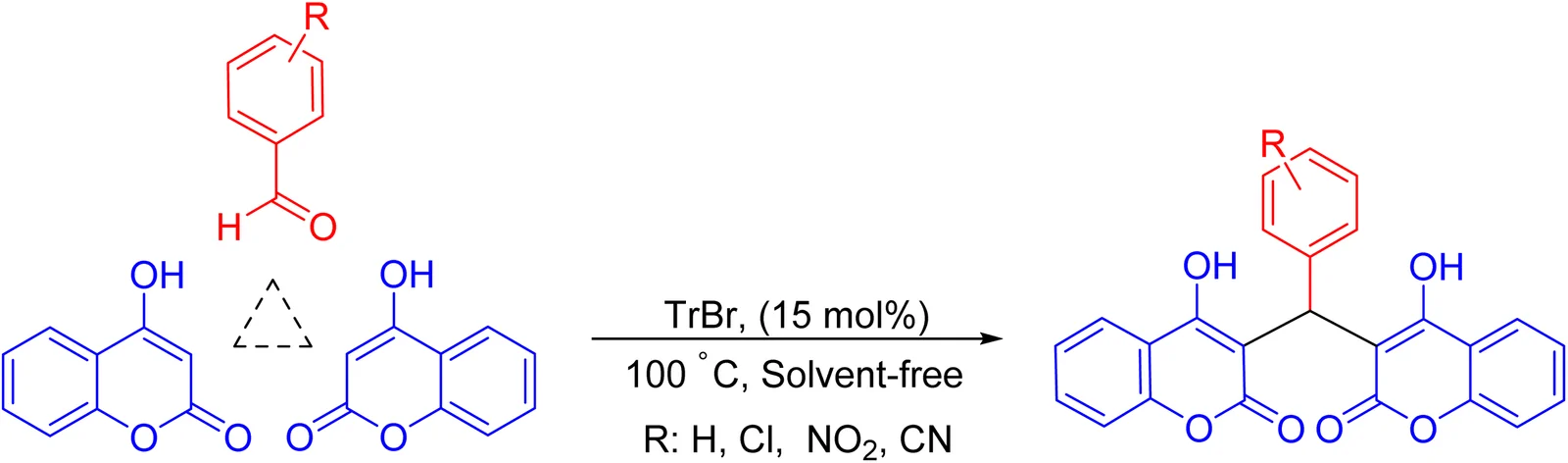
Construction of biscoumarins utilizing trityl bromide.

In addition to trityl chloride, trityl bromide has also been used as a source of carbocations for catalyzing organic reactions. Biscoumarins have been prepared by the reaction of 2 equiv. of 4-hydroxycoumarin with 1 equiv. of aromatic aldehyde in the presence of catalytic amounts of trityl bromide at 100 °C in the absence of solvent (Scheme 32).^22^ Some heterocyclic aldehydes including terephthalaldehyde, cinnamaldehyde, pyridine-4-carbaldehyde and indole-3-carbaldehyde was successfully used in this reaction to give the corresponded products.

**Scheme 33:**
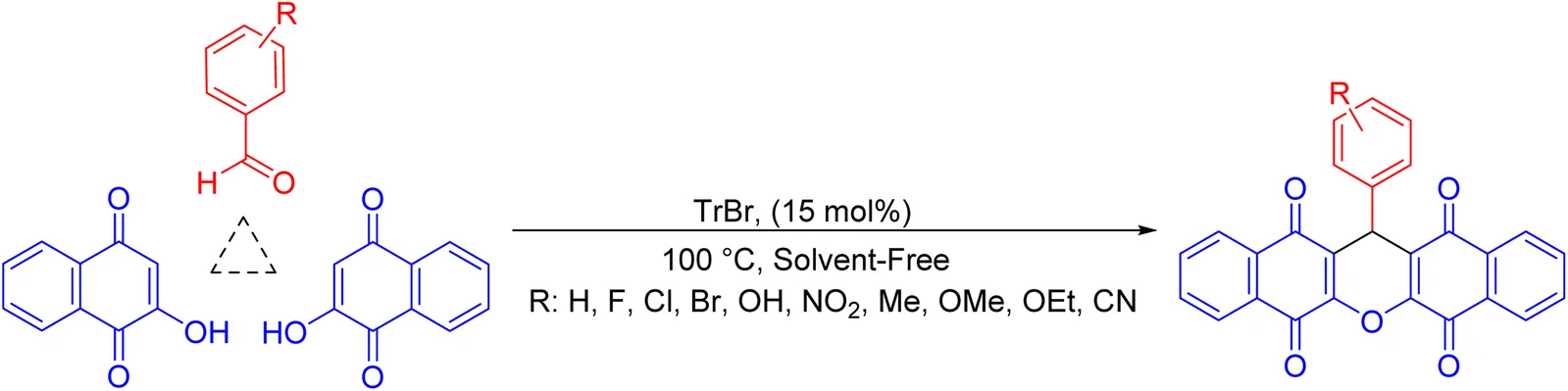
Production of dibenzo xanthene tetraone derivatives using trityl bromide.

Also, condensation of two moles of 2-hydroxynaphthalene-1,4-diene with one mole of aromatic aldehyde using catalytic amounts of trityl bromide has been reported to produce dibenzoxanthene tetraone derivatives at 100 °C in the absence of solvent (Scheme 33).^22^

**Scheme 34:**
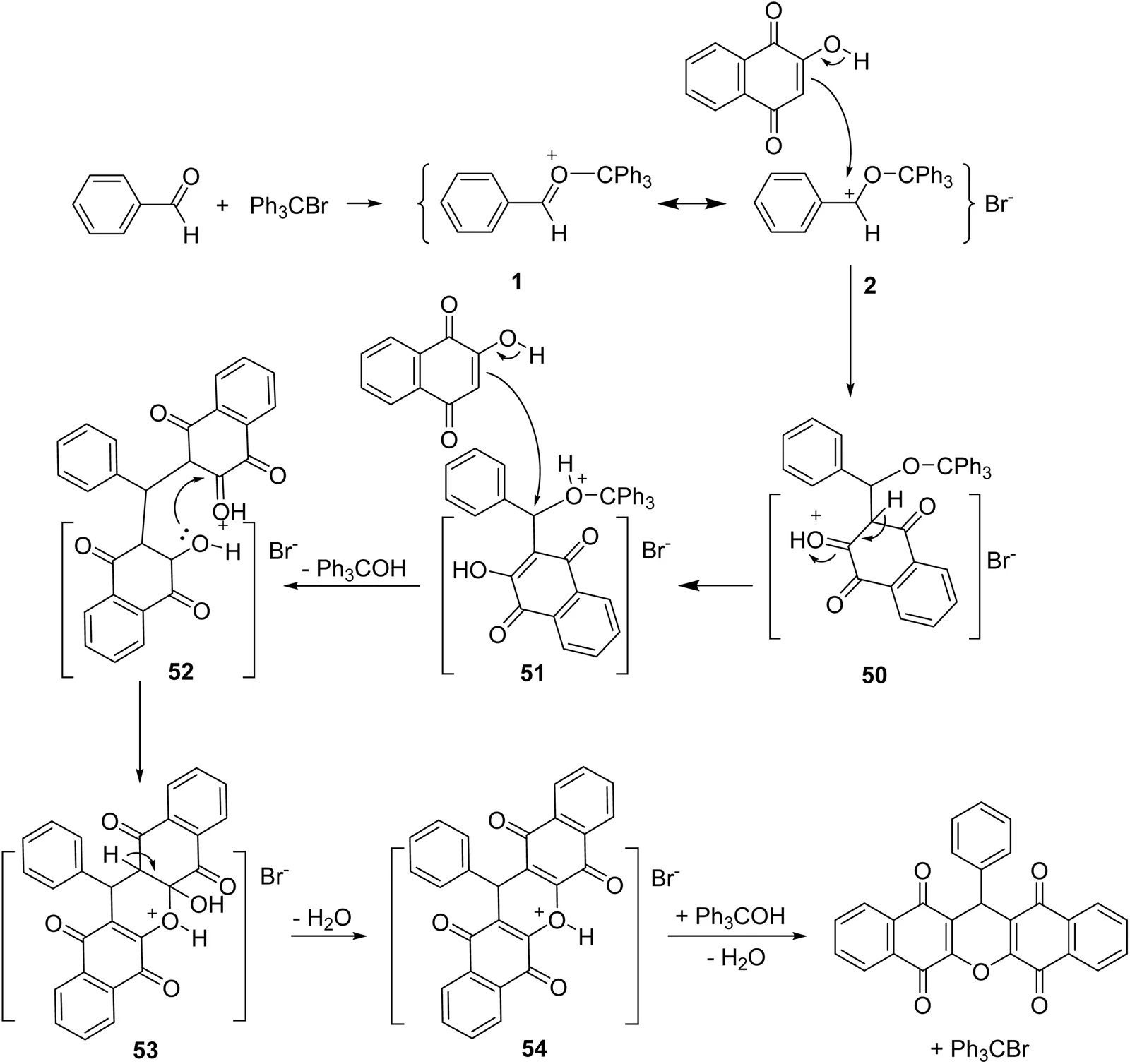
Mechanism of fabricating dibenzoxanthene tetraone derivatives catalyzed by trityl bromide.

In this reaction, 2-hydroxynaphthalene-1,4-diene acts as a nucleophile to attack activated aldehydes by trityl carbocation (1 and 2) to produce intermediate (50). Next, proton transfer produces intermediate (51) and another molecule of 2-hydroxynaphthalene-1,4-diene attacks to it and removes one molecule of trityl alcohol to produce intermediate (52). Intermediate (52) undergoes intramolecular nucleophilic attack and cycloaddition produced intermediate (53), which is then removed one molecule of water to produce intermediate (54). Finally, trityl bromide is produced again by the interaction of intermediate (54) with trityl alcohol and removes one molecule of water (Scheme 34).^22^

### The three-component synthesis of pyrano[2,3-*d*]pyrimidine diones

**Scheme 35:**
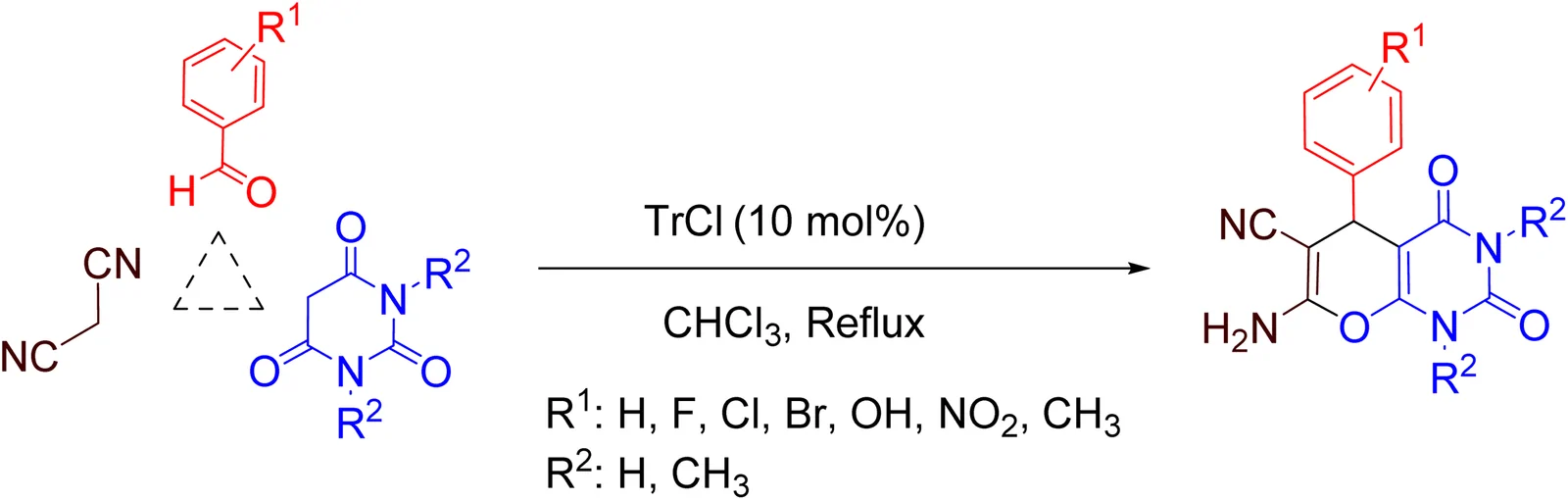
The construction of pyrano[2,3-*d*]pyrimidine diones utilizing TrCl.

Another TrCl-catalyzed three-component reaction is the condensation of aromatic aldehydes with malononitrile and barbituric acid to produce pyrano[2,3-*d*] pyrimidine diones compounds. This reaction was carried out in chloroform as a solvent under reflux condition (Scheme 35).^23^

**Fig. 2:**
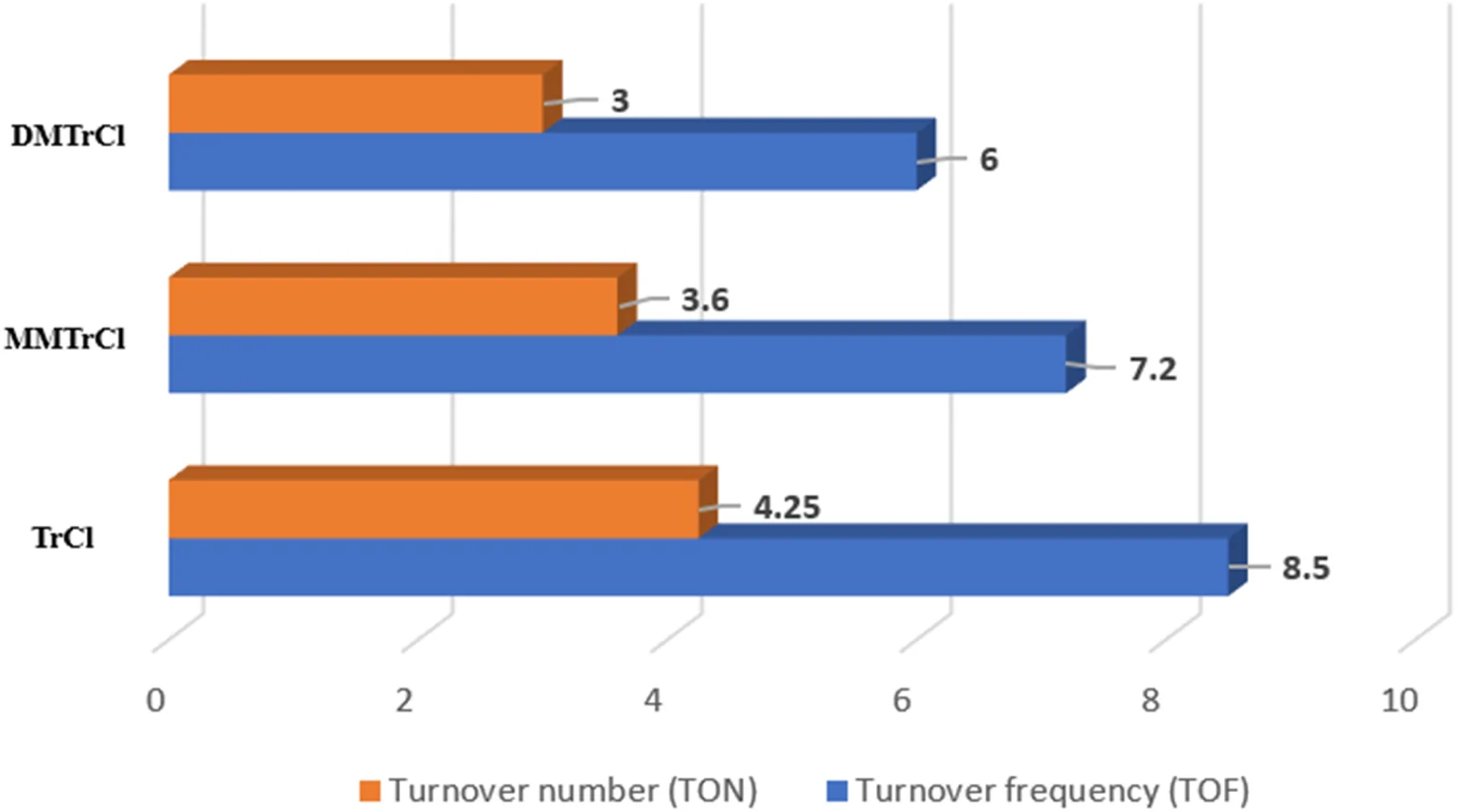
Catalytic utility of some triarylmethyl chlorides on the reaction of 2,4-dichlorobenzaldehyde with barbituric acid and malononitrile in chloroform under reflux condition.

The catalytic ability of MMTrCl and DMTrCl was compared with TrCl in this reaction. TON and TOF values of TrCl were compared with those of MMTrCl and DMTrCl on the three-component reaction of 2,4-dichlorobenzaldehyde, barbituric acid and malononitrile for the synthesis of the related pyrano[2,3-*d*]pyrimidine dione (Fig. 2). According to Fig. 2, TrCl is more effective than the other trityl derivatives in terms of TOF and TON.^23^

**Scheme 36:**
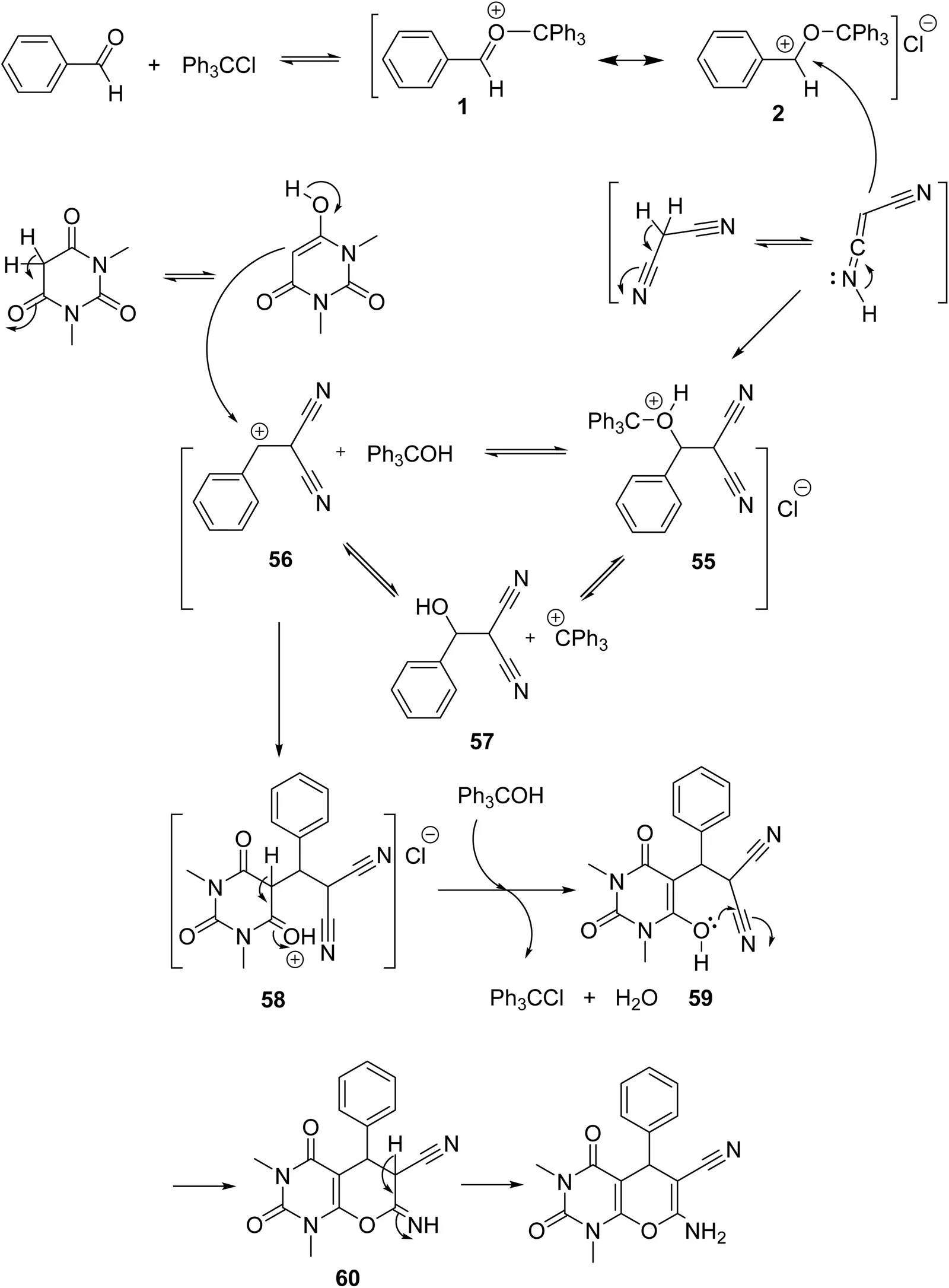
The mechanism for the construction of pyrano[2,3-*d*]pyrimidine diones using TrCl.

In this reaction, the activated forms of aldehyde (1 and 2) were obtained by the reaction of aldehyde with trityl cation, which *in situ* generated in the reaction media. Malononitrile reacts with the activated aldehyde to obtain (55), which is in equilibrium with (56) and (57). Barbituric acid in enol form reacts with intermediate (56) to afford cyanoolefin (58). Deprotonation of (58) leads to (59) and also TrCl is regenerated. Intramolecular nucleophilic attack in intermediate (59) leads to (60), which is converted to the final product after tautomerization (Scheme 36).^23^

### The condensation reaction of sulfonamides with aldehydes and ketones

**Scheme 37:**
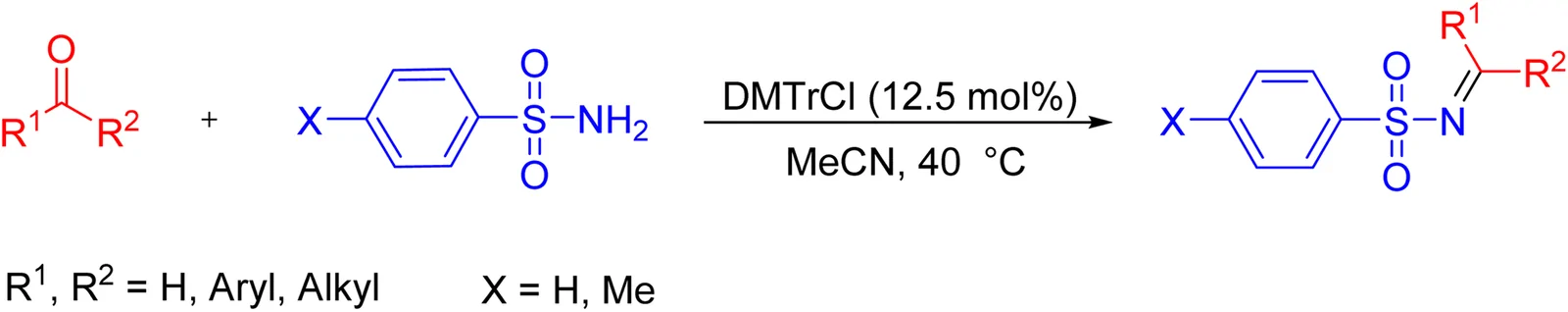
Condensation of sulfonamides with aldehydes and ketones utilizing DMTrCl.

The condensation of sulfonamides with aldehydes and ketones in the presence of DMTrCl as a catalyst in acetonitrile solvent at 40 °C has been studied.^24^ Various aromatic aldehydes with electron-donating and electron-withdrawing substituents and halogens have been used in this reaction. Heterocyclic aldehydes, aliphatic aldehydes, and terephthalaldehyde have also been successfully used in the reaction. The yields of products for aliphatic and aromatic ketones have been reported to be lower than for the aldehydes used in the reaction (Scheme 37).^24^

**Scheme 38:**
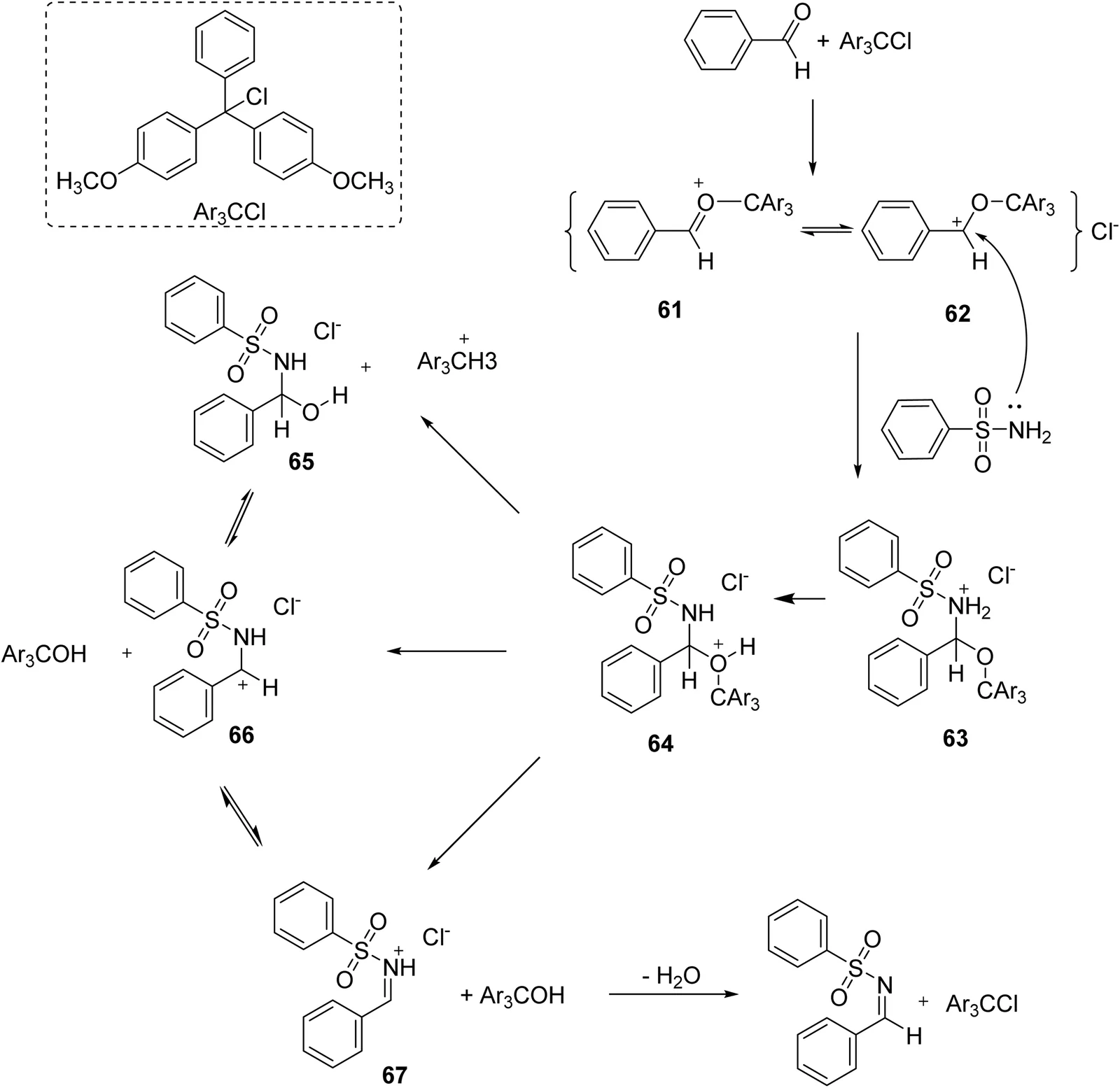
Mechanism of condensation of sulfonamides with aldehydes and ketones in the presence of DMTrCl.

In this reaction, after the interaction between the carbonyl group and dimethoxytrityl chloride, intermediates (61) and (62) are produced as activated forms of aldehyde in the reaction medium. Sulfonamide reacts with the aforementioned intermediates to produce intermediate (63), which, upon proton transfer, produces (64). (64) can prepare intermediates (65) to (67). Intermediate (64) is converted to intermediates (67) by losing one molecule of dimethoxytrityl alcohol. Finally, by reacting (62) with dimethoxytrityl alcohol and removing one molecule of water, the desired product is produced along with dimethoxytrityl chloride and the catalyst is regenerated (Scheme 38).^24^

### The four-component synthesis of highly functionalized dihydro-2-oxopyrroles

The four-component synthesis of highly functionalized dihydro-2-oxopyrroles and bis-dihydro-2-oxopyrrole derivatives was reported by the reaction of dialkyl acetylenedicarboxylates, various amines, and formaldehyde in the presence of catalytic amounts of TrCl in ethanol as a solvent under mild condition at room temperature (Table 2).^25^

**Table 2:** The preparation of highly functionalized dihydro-2-oxopyrroles

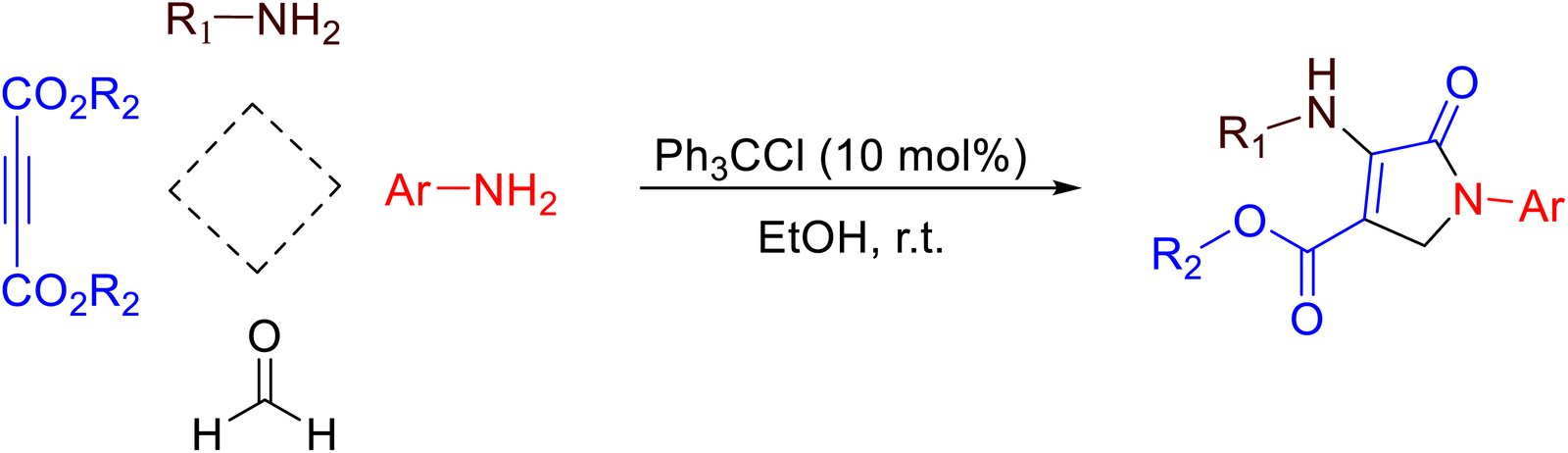
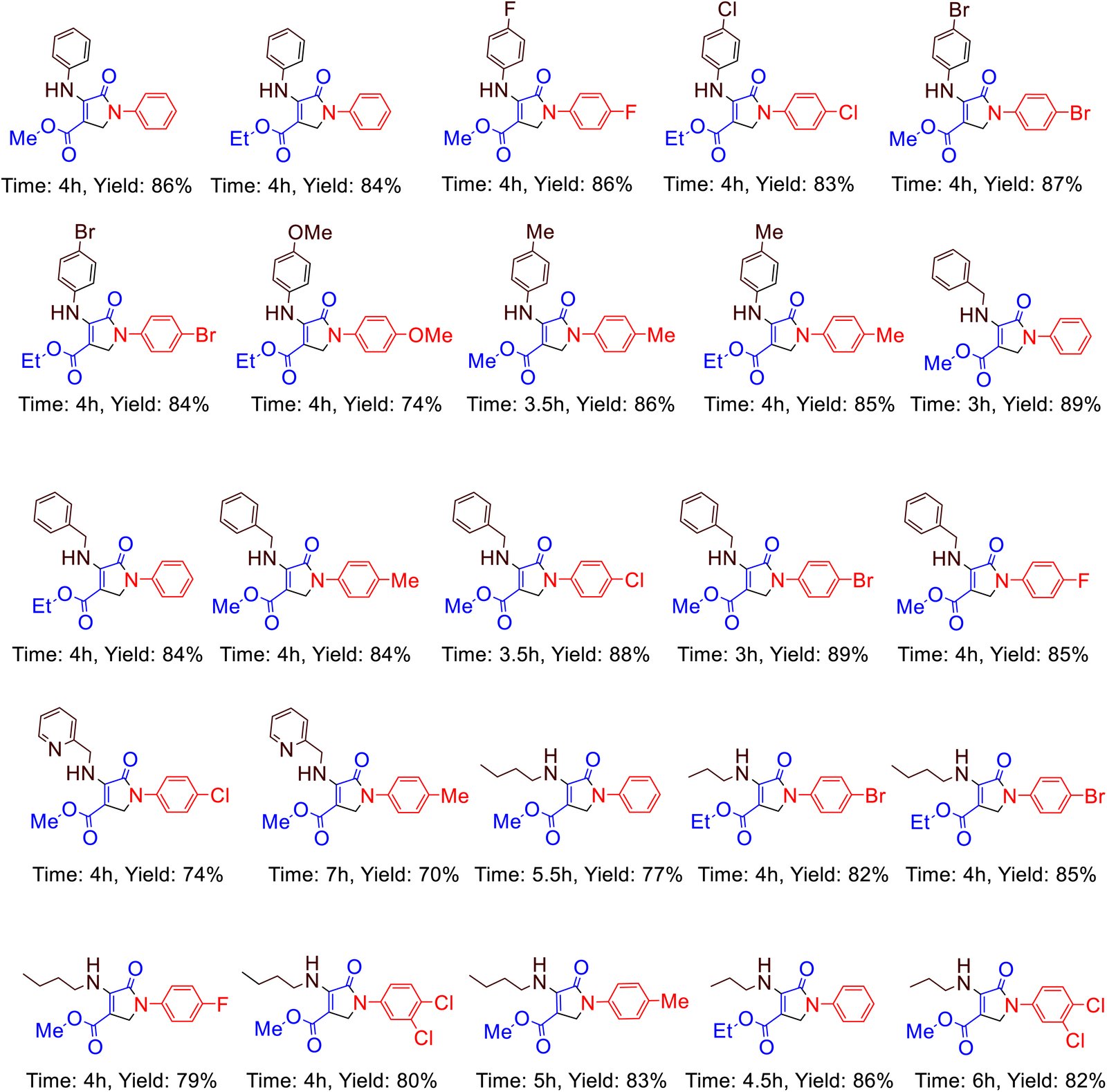

In this method, products were produced in good to high yields and short reaction times under mild and green reaction condition, and purified without the need for column chromatography. At the beginning of the reaction, the substrates are completely soluble in ethanol and form a homogeneous mixture. However, at the end of the reaction, the product becomes suspended and finally precipitates. The resulting precipitate is easily purified by simple filtration and finally by nebulization.

Under the optimized conditions, a pseudo seven-component transformation involving dialkyl acetylenedicarboxylate, ethane-1,2-diamine, aromatic amine, and formaldehyde was successfully performed, affording bis-dihydro-2-oxopyrrole derivatives (Table 3).^25^

**Table 3:** The fabrication of polyfunctionalized bis-dihydro-2-oxopyrroles

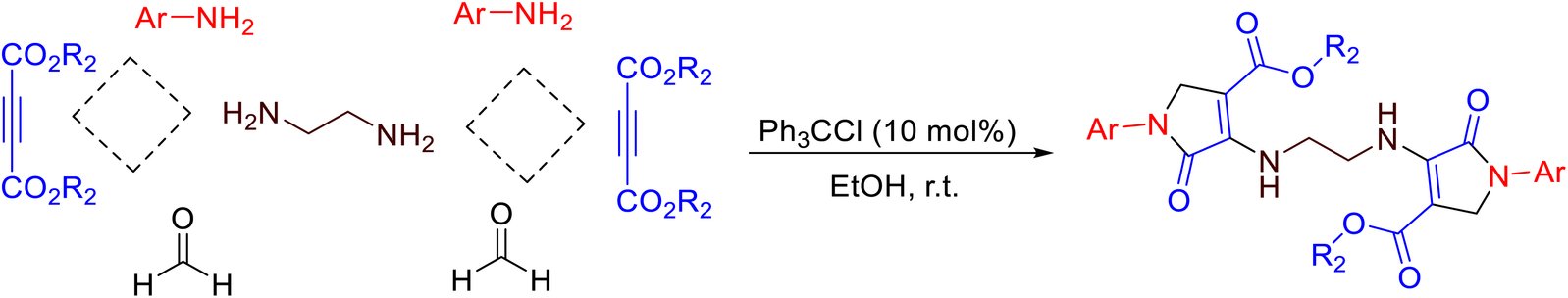
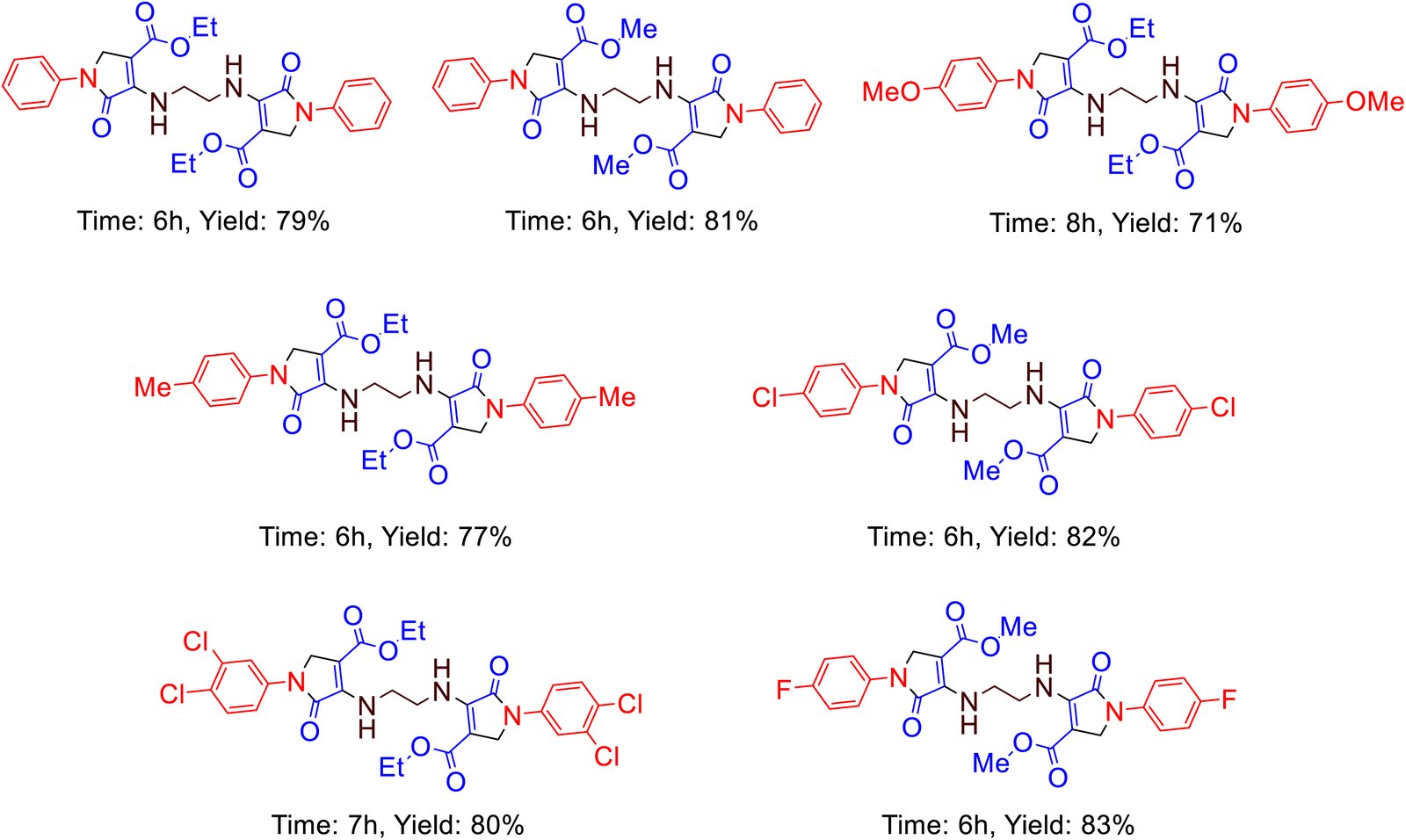

**Scheme 39:**
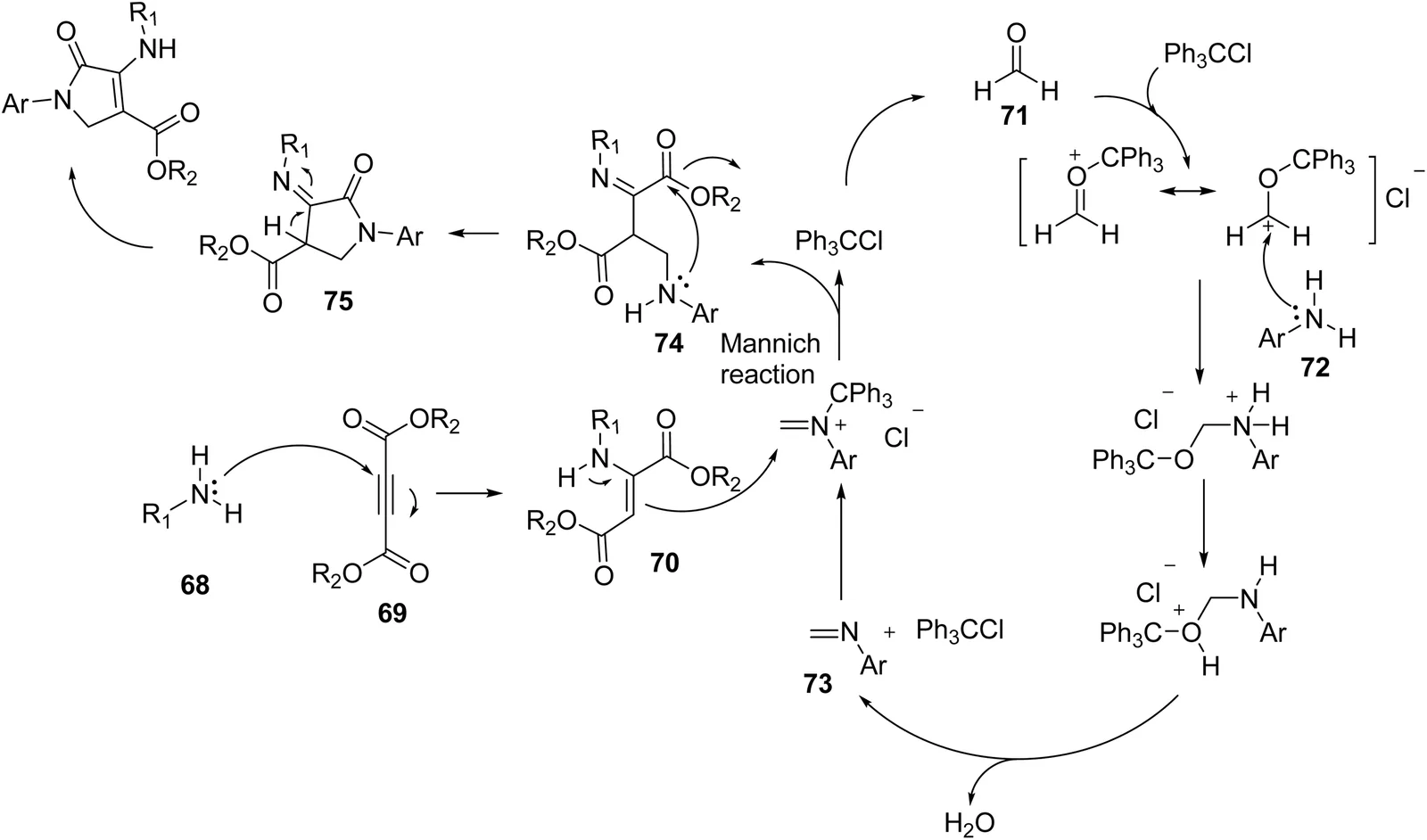
Mechanistic pathway for the construction of dihydro-2-oxopyrroles.

In the presented mechanism, firstly, by the reaction of amine (68) with dialkyl acetylenedicarboxylate (69) intermediate (70) was prepared. Besides of this reaction, by the reaction between formaldehyde (71) and amine (72) which is activated by TrCl as a catalyst, imine (73) was produced. In the next step, by the Mannich type reaction of intermediate (70) with imine (73) intermediate (74) was furnished, which *via* the cyclization reaction transformed to intermediate (75). Lastly, dihydro-2-oxopyrrole produced as a final product after tautomerization in the structure of intermediate (75) (Scheme 39).^25^

### The pseudo-five component construction of highly substituted piperidines

**Scheme 40:**
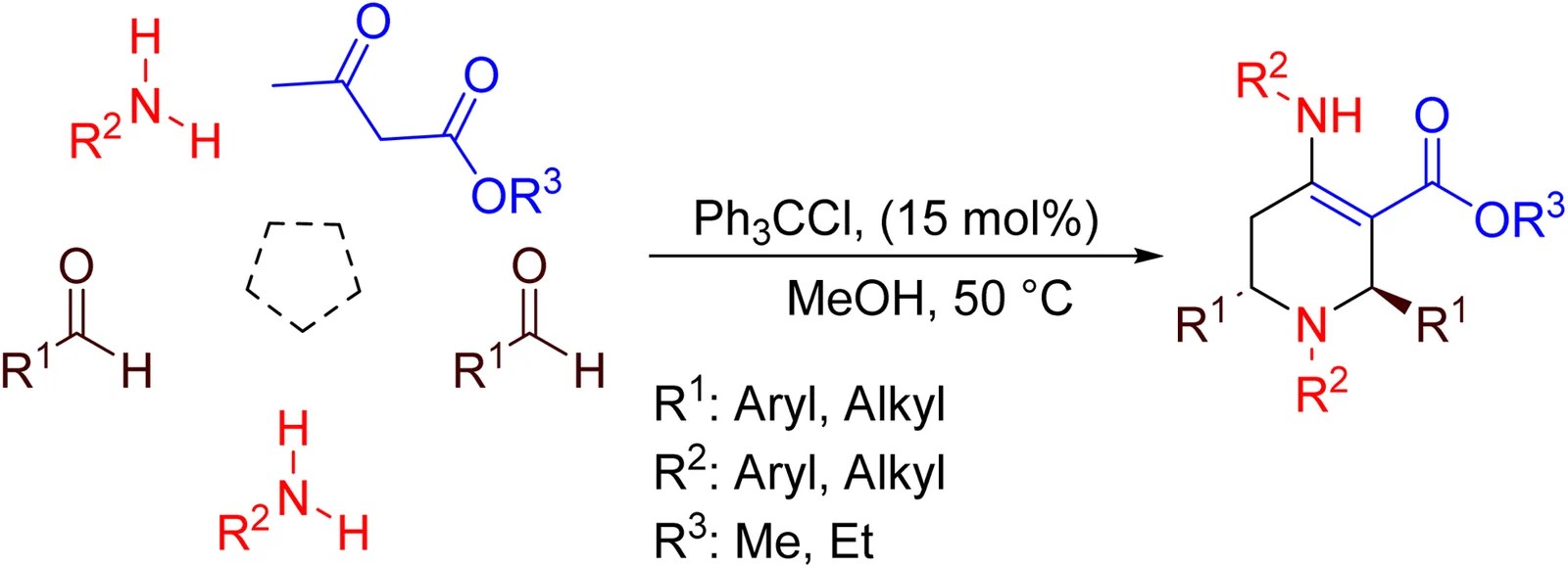
The synthesis of highly substituted piperidines.

The pseudo-five component of aromatic aldehydes, amines and beta-ketoesters for the synthesis of five substituted piperidines was reported as a diastereoselective synthesis using TrCl in methanol as a solvent under mild reaction at 50 °C (Scheme 40).^26^ In this method, using of an inexpensive homogeneous catalyst, products were produced with relatively short reaction times and good to high yields. Easy separation of products, diastereoselectivity, and available starting materials are the features of this method.

**Scheme 41:**
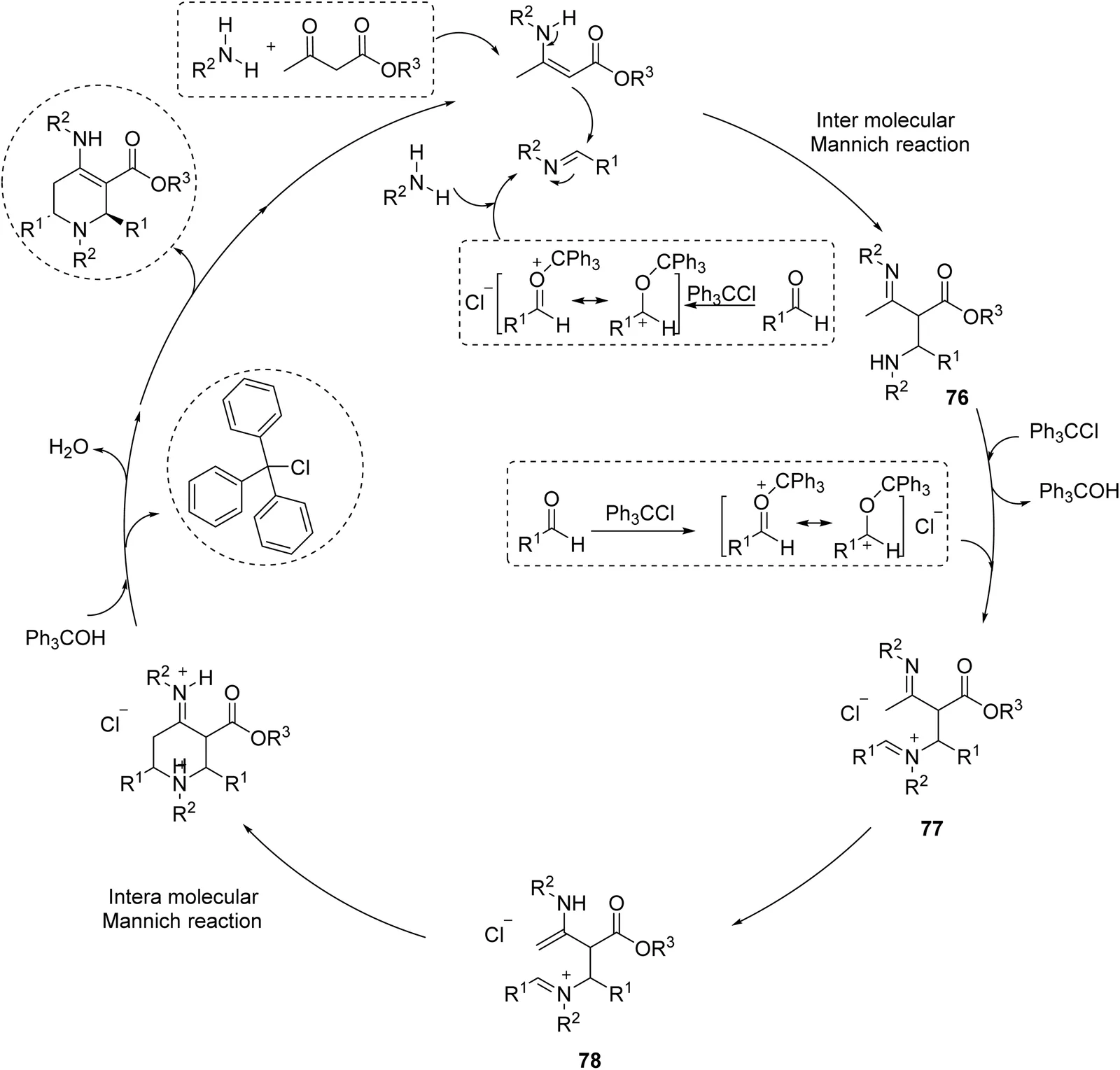
The suggested mechanism for the synthesis of highly substituted piperidines.

In the given proposed mechanism, one molecule of amine reacted with aldehyde which activated by triphenyl methyl carbocation generated from TrCl to give imine compound. In other hand, by the reaction of one molecule of amine with beta-ketoester the related enamine was obtained. The reaction between prepared imine and enamine in previous step intermediate (76) produced as Mannich-type reaction, which reacted with another aldehyde to furnish intermediate (77) which converted to (78) after tautomerization. Then, by the intra molecular Mannich-type reaction in (78) the ring was closed and the final product was obtained after tautomerization (Scheme 41).^26^

### The three-component fabrication of 4,7-dihydro[1,2,4]triazolo[1,5-*a*]pyrimidine-6-carboxamides

**Scheme 42:**
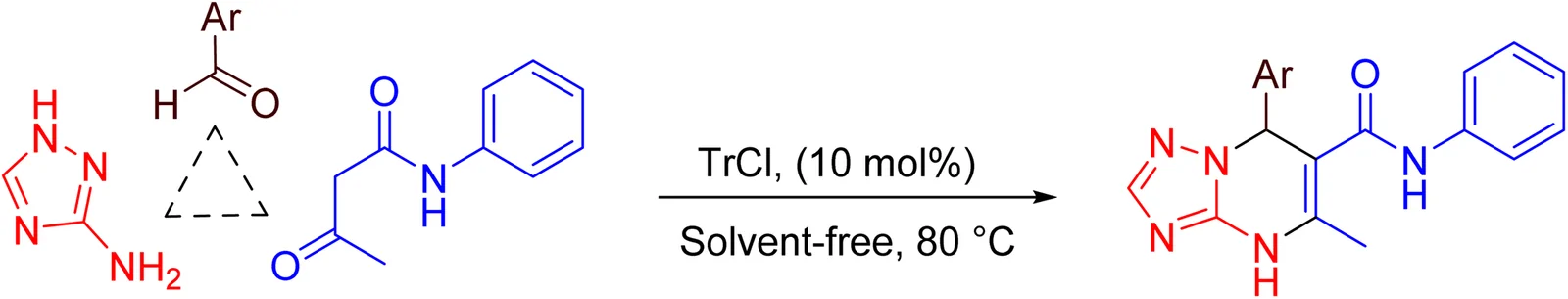
The construction of 4,7-dihydro[1,2,4]triazolo[1,5-*a*]pyrimidine-6-carboxamides.

In another study, the preparation of 4,7-dihydro[1,2,4]triazolo[1,5-*a*]pyrimidine-6-carboxamide compounds was reported by the three-component condensation reaction of various aryl aldehyde, with 3-amino-1,2,4-triazole and acetoacetanilide using TrCl as a catalyst at 80 °C in the absence of solvent with acceptable results (Scheme 42).^27^

The characteristics of this method include short reaction times, good and excellent yields, and easy operation. All products were obtained in pure form through simple purification without the need for a chromatography column, which are important factors in reducing environmental pollution.

### The three-component synthesis of 3-amino-2-oxofuranes

**Scheme 43:**
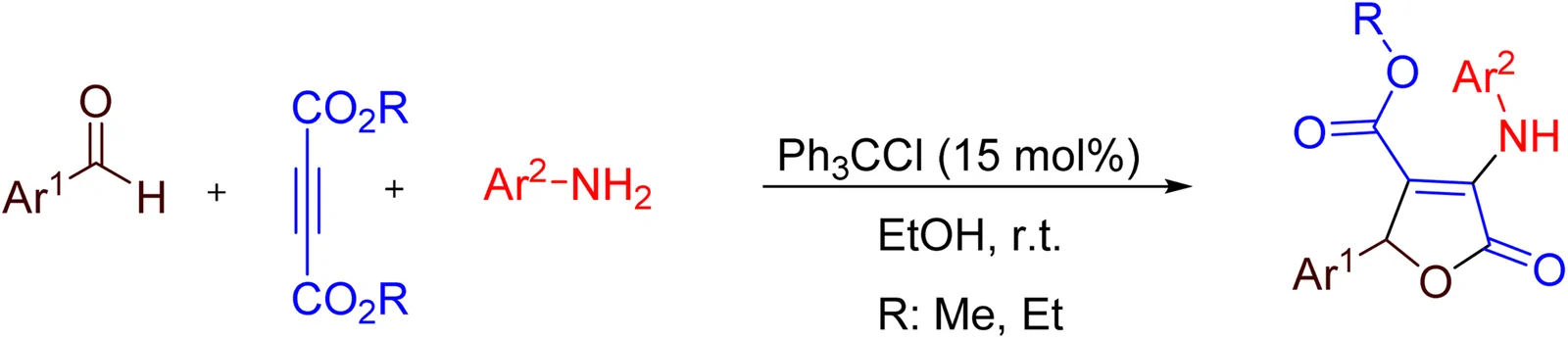
The fabrication of 3-amino-2-oxofuranes.

In another investigation, the three-component synthesis of 2,5-dihydro-2-oxo-5-aryl-3-(arylamino) furan-4-carboxylate using TrCl as a catalyst was studied. In this work, 3-amino-2- oxofurane products was prepared by the reaction of aromatic amines, diethyl and/or dimethyl acetylenedicarboxylates and aryl aldehydes using TrCl in ethanol as a solvent at room temperature (Scheme 43).^28^ The purification of the products in this method is very simple and obtained products as precipitate was filtered and washed with ethanol to produce the pure product.

**Scheme 44:**
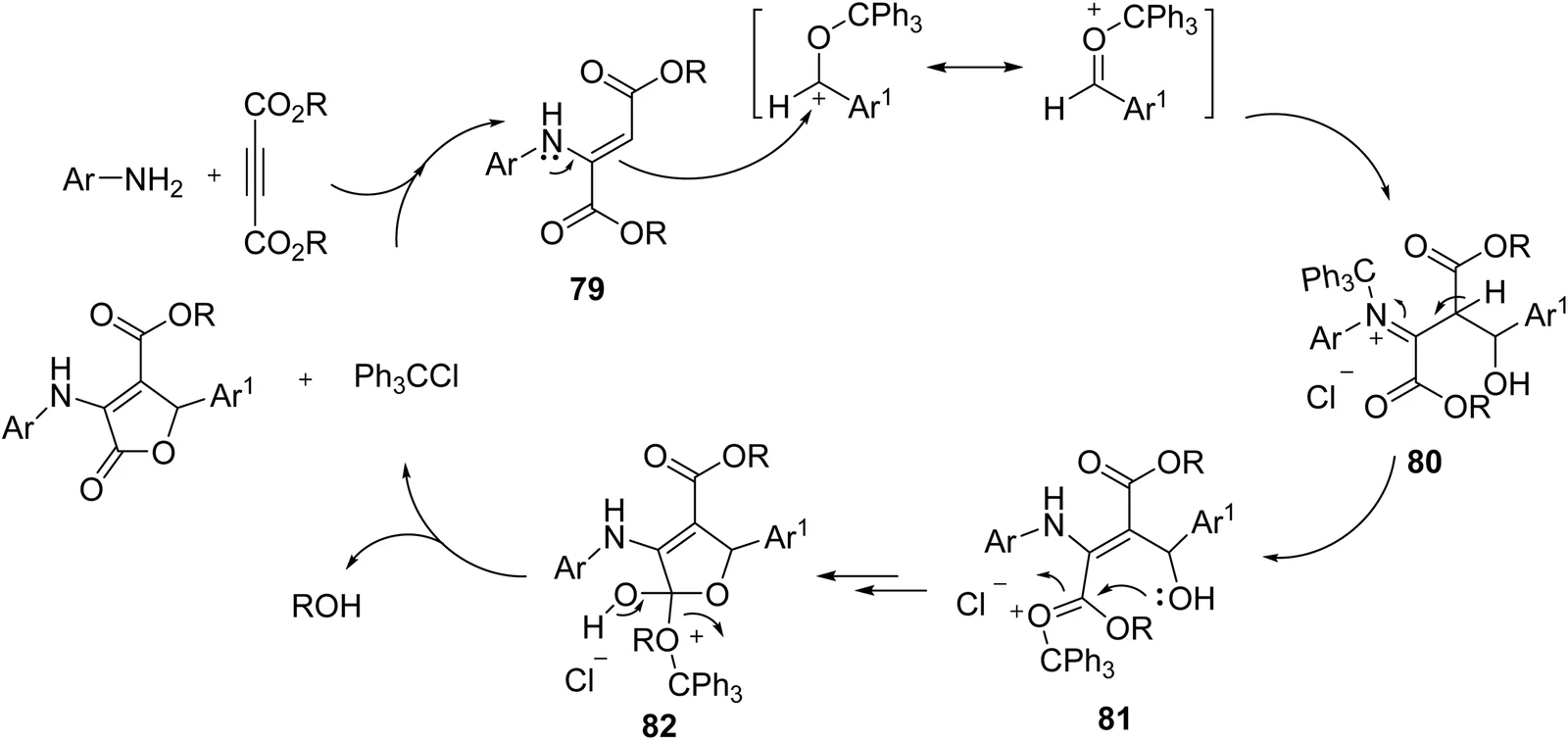
The mechanism for the fabrication of 3-amino-2-oxofuranes.

In the proposed reported mechanism, firstly, amine was reacted to acetylenedicarboxylate to give A. Intermediate (79) was reacted to activated aldehyde which activated with tri phenyl methyl carbocation generated from TrCl to obtain (80) which tautomerizes to (81). By ring closing in intermediate (81), intermediate (82) was prepared which converted to final product by elimination of one molecule of alcohol (Scheme 44).^28^

## Some other organic reactions catalyzed by TrCl

### Diels–Alder reaction catalyzed by TrCl

**Scheme 45:**
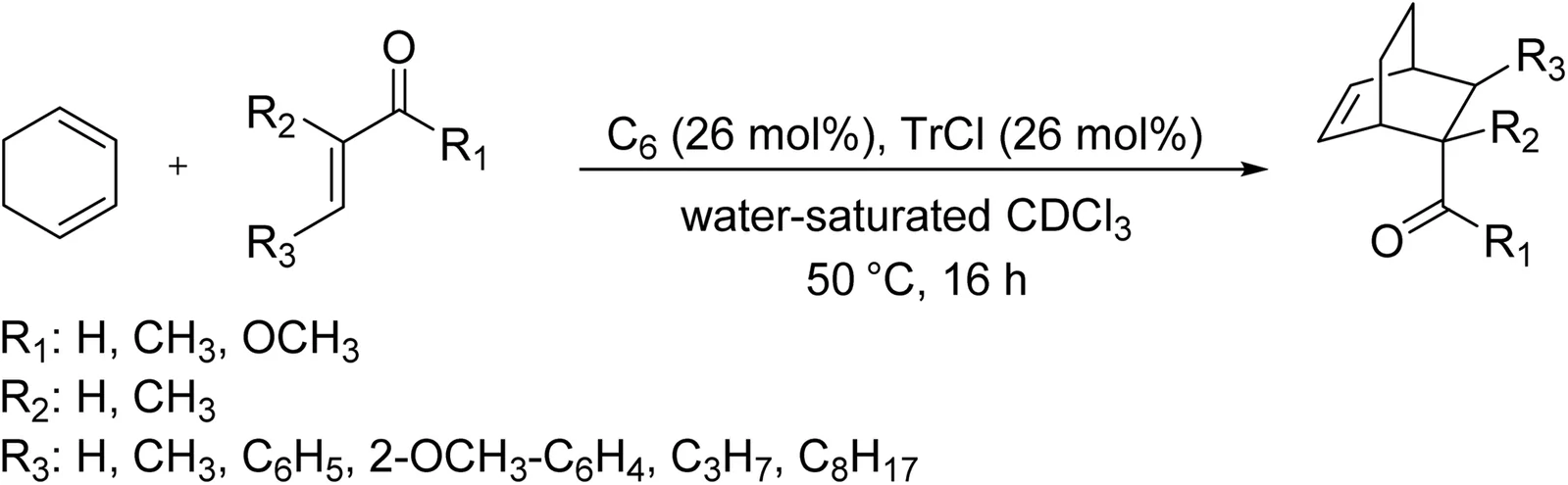
Diels–Alder reaction of cyclohexa-1,3-diene with various dienophiles.

**Scheme 46:**
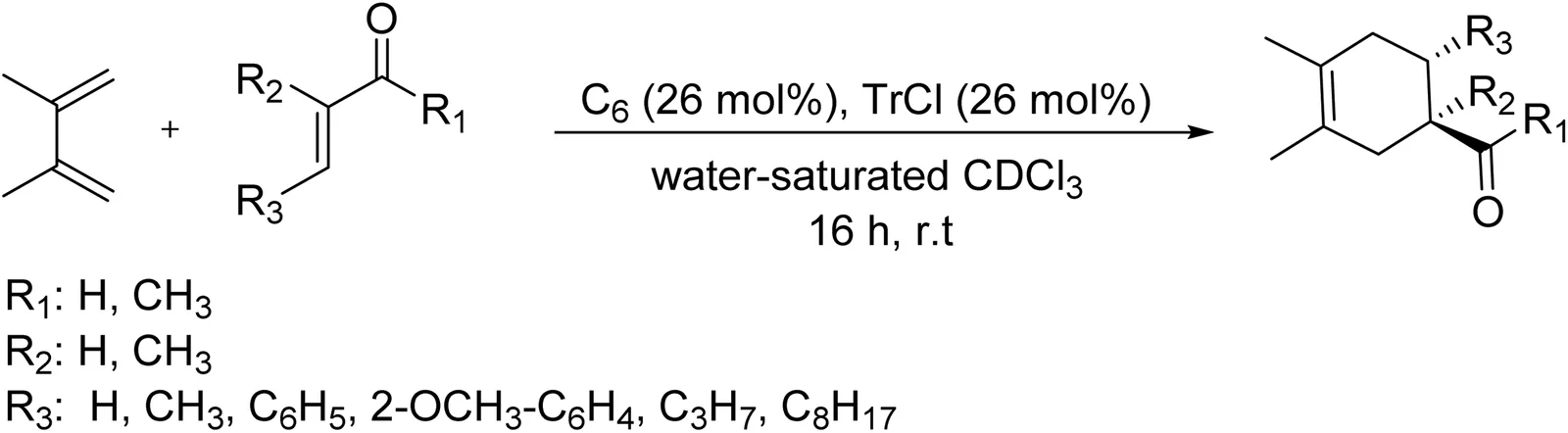
Diels–Alder reaction of 2,3-dimethylbutadiene with various dienophiles.

**Scheme 47:**
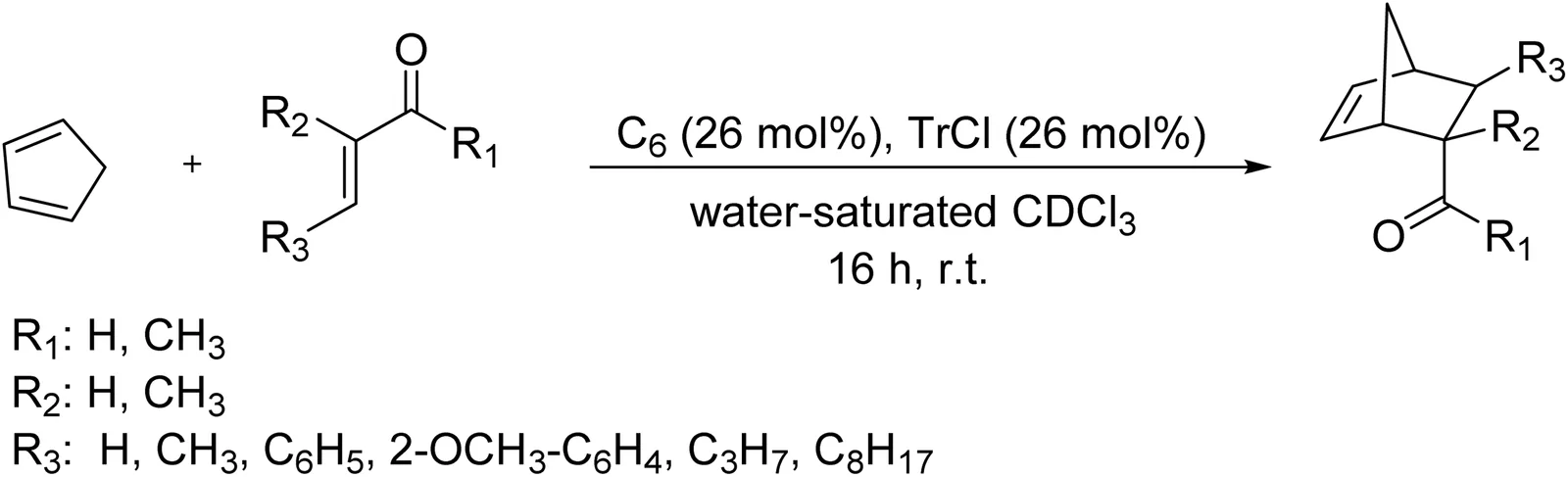
Diels–Alder reaction of cyclopentadiene with various dienophiles.

Recently, to activate TrCl, it was introduced into the nanoscale space of the hexameric resorcinol capsule and hydrogen bonded between the hydroxy group and the trityl chloride ion in this space. The trityl carbocation in this cavity catalyzed the Diels–Alder reaction with endo-route by activating the dienophile. In this method, the nanoscale space and the embedded carbocation catalyst were used as a complementary method for carbocation catalysis. In this catalytic host–guest system, the π-electron-rich space in the resorcinol structure interacts well with the trityl carbocation, and this carbocation is further stabilized by the cation–π interactions (Schemes 45–47).^29^

The substrate scope of the Diels–Alder reaction in the presence of C_6_ was investigated by the reaction of cyclohexa-1,3-diene with a variety of different dienophiles and under the optimal reaction conditions, produces corresponding products in moderate to high yields. From the obtained results, it was found that the yield of the Diels–Alder reaction between cyclohexa-1,3-diene with different dienophiles in the presence of C_6_ depends on the length of the alkyl chain R_3_ at the *β*-position. Thus, with increasing chain length R_3_ from CH_3_, to *n*-C8H_17_ decreased the conversion of the reaction significantly from 92 to 46% (Scheme 45).^29^

Diels–Alder reaction of 2,3-dimethylbuta-1,3-diene with cinnamic aldehyde derivatives produced the corresponding adducts in high yield and selectivity at room temperature. A significant decrease in reactivity with sterically hindered aldehydes at the R_3_ position led to polymerization and reduced yield of the Diels–Alder adducts. Increasing temperature and reaction time did not increase the reaction yield. The corresponding reaction with the *o*-OMe-C_6_H_4_ substituted cinnamic aldehyde, at the R_3_ position led to better conversion. Methyl vinyl ketone showed comparable reactivity with acrolein and provided the desired cycloadduct in quantitative terms (Scheme 46).^29^

The use of cyclopentadiene as a diene in Diels–Alder reaction increased the reactivity and had a significant effect on the rate and efficiency of the reaction. Increasing the size of the R_3_ group in aldehydes changed the selectivity, but increasing the reaction time to 16 h produced high-conversion adducts for aldehydes. Finally, methyl vinyl ketone as a ketone was also served as a very good dienophile in this reaction and produced the corresponding cyclic product in high yield (Scheme 47).^29^

### Carbonyl-ene cyclization and [2 + 2] cycloaddition catalyzed by catalytic system containing of trityl halides and thioureas

Trityl halides such as trityl chloride and trityl bromide in the presence of thiourea was reported as a Lewis acid carbocationic catalytic system for the intramolecular carbonyl-ene cyclization and intramolecular [2 + 2] cycloaddition reaction. In this mild, green and metal-free method by *in situ* generated carbocation, the carbonyl group in the reactant was activated and accelerated the reaction.^30^

**Scheme 48:**
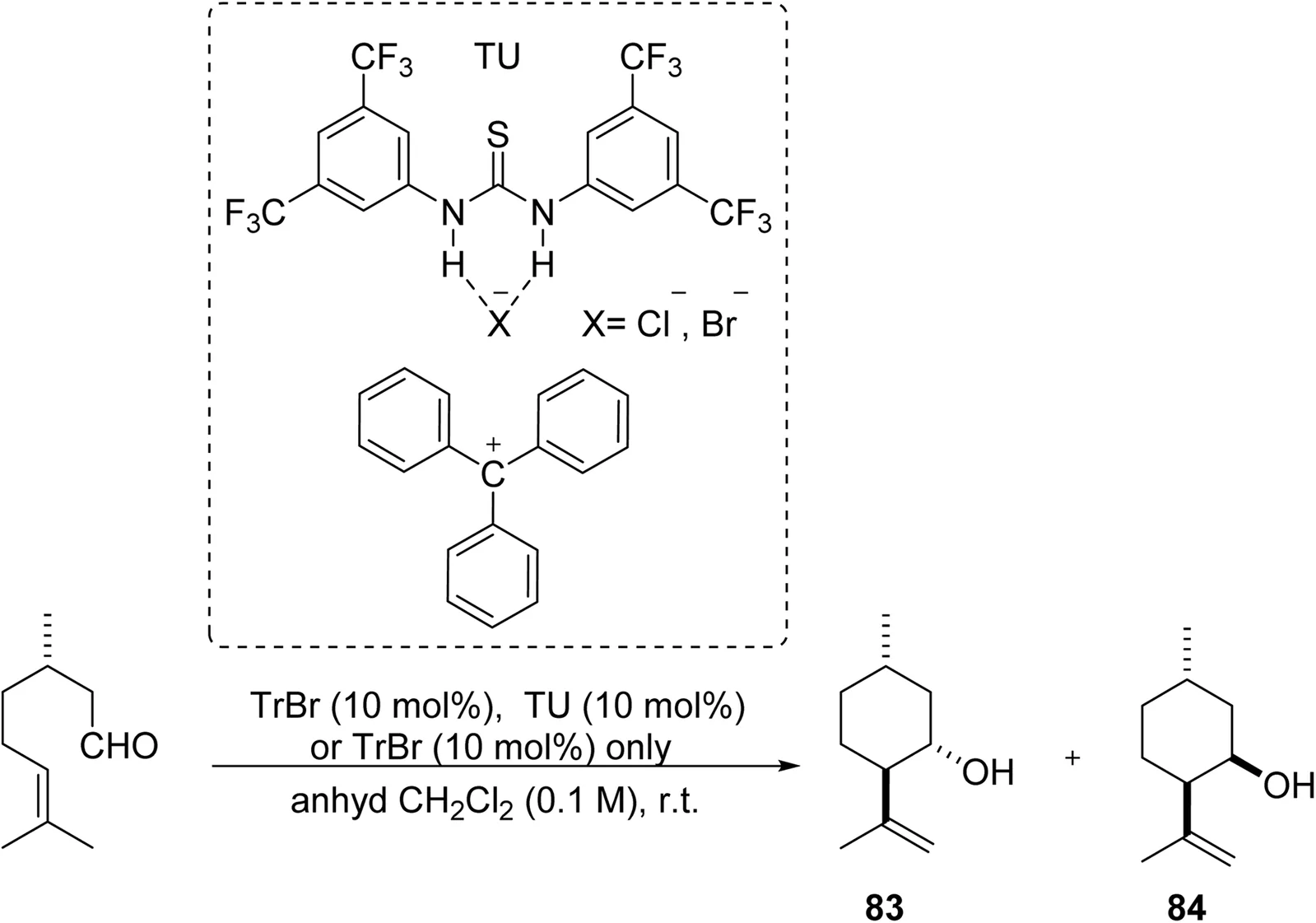
Carbonyl-ene cyclization reaction.

The cyclization of carbonyl-ene (−)-citronellal was studied in the presence of a cooperative catalytic system consisting of trityl bromide (TrBr) or its analogues and thiourea (TU) in anhydrous dichloromethane as a model reaction. By using 10 mol% of each of TrBr and TU, a mixture of (83) and (84) was isolated in 96% yield after four hours at room temperature (2/3 = 61 : 39) (Scheme 48). This reaction was also carried out only in the presence of 10 mol% TrBr which a mixture of 2 and 3 was isolated in 91% yield after five hours at room temperature (2/3 = 59 : 41).^30^ The same reaction was relatively slow in toluene, which could be attributed to the poor solubility of the reagents or catalysts in this solvent.^30^

**Scheme 49:**
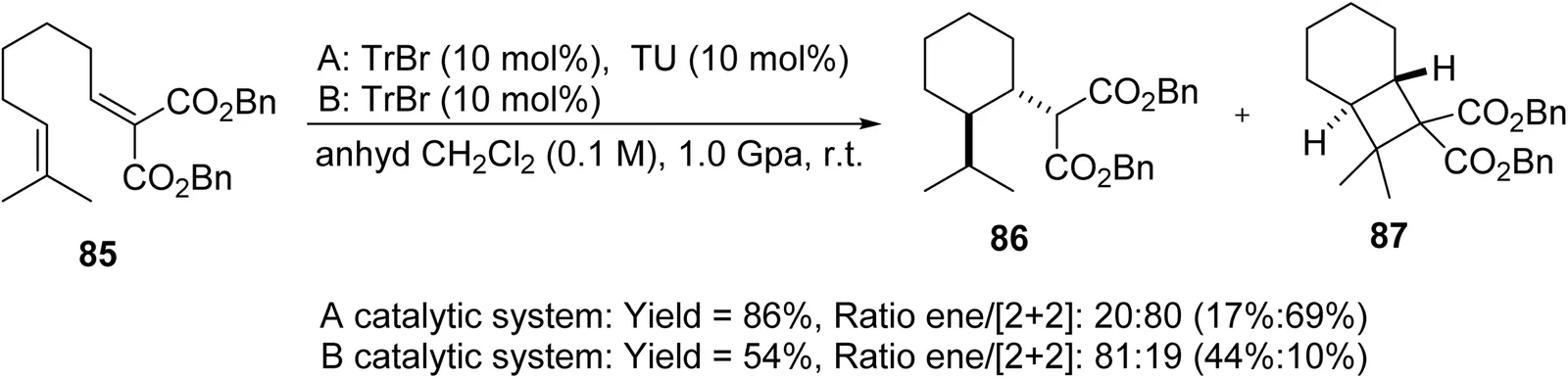
Intramolecular [2 + 2] cycloaddition reaction.

In the next step, the reaction of activated dienes was studied. Since the reaction was very slow under our standard conditions, the reaction was carried out under pressure. In the reaction of malono diester (85) in dichloromethane using TrBr and TU as a catalytic system (system A) at 1.0 GPa and room temperature for 72 h, the ene type product (86) and the [2 + 2] cycloaddition product (87) were produced in 17% and 69% yields, respectively. The same reaction catalyzed with trityl bromide alone (system B) was relatively slow and the ene type reaction was carried out better, with (86) being formed as the main product (Scheme 49).

### Diels–Alder reactions catalyzed by chiral tritylium salts

**Scheme 50:**
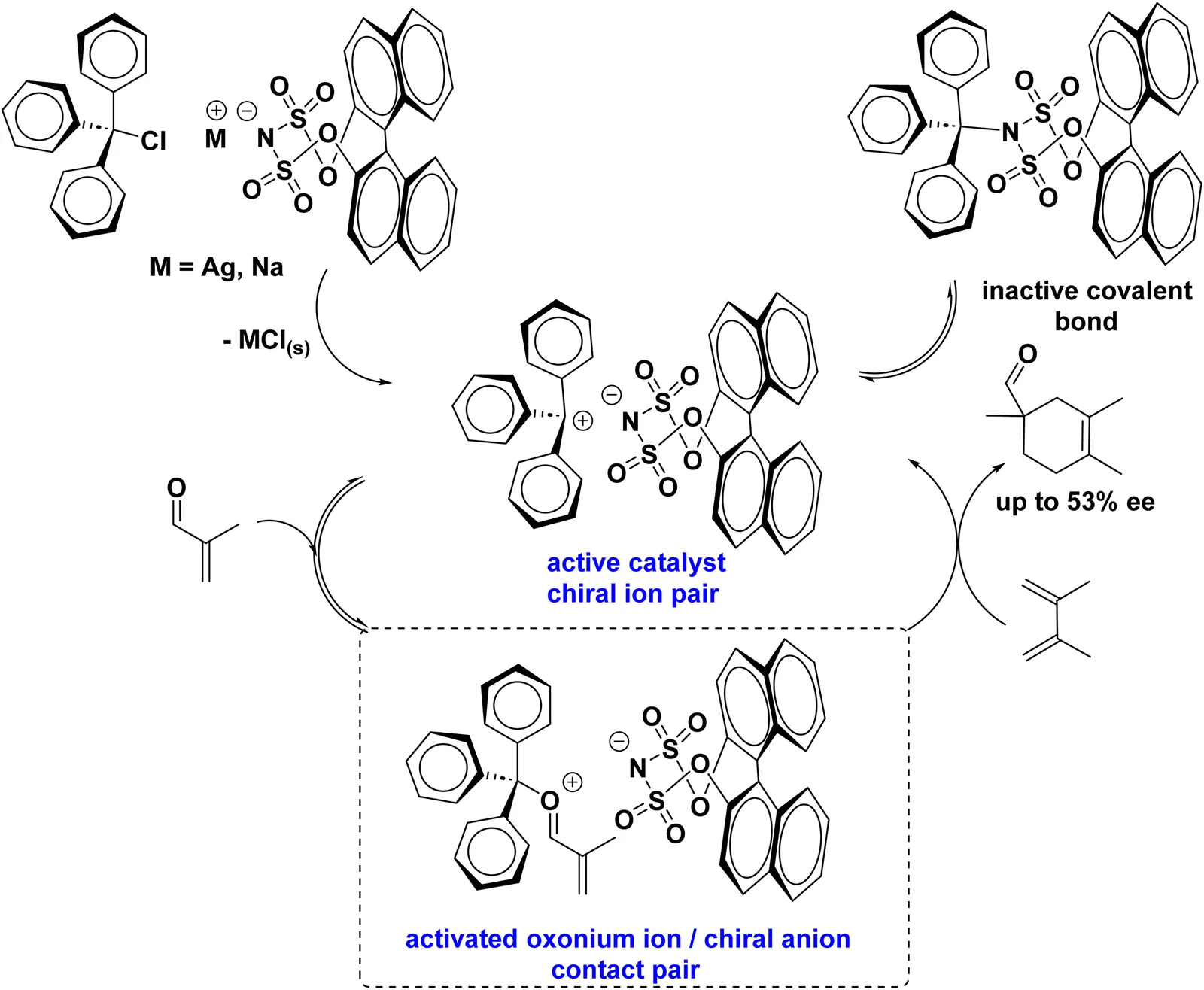
Diels–Alder reaction catalyzed by chiral tritylium salts.

Chiral tritylium salts were *in situ* produced from a mixture of triphenylmethyl chloride derivatives and chiral phosphoramide, phosphonate, bis(sulfuryl)amide or bis(sulfonyl)amide. It is found that a mixture of bis(sulfuryl)amide and tritylium ion salt catalyzed the Diels–Alder reaction with enantiomeric excess up to 53%. Exceptional Lewis acidity and mild catalytic ability of tritylium carbocation besides of chiral anion leads to highly efficient protocol in organic asymmetric synthesis (Scheme 50).^31^

**Scheme 51:**
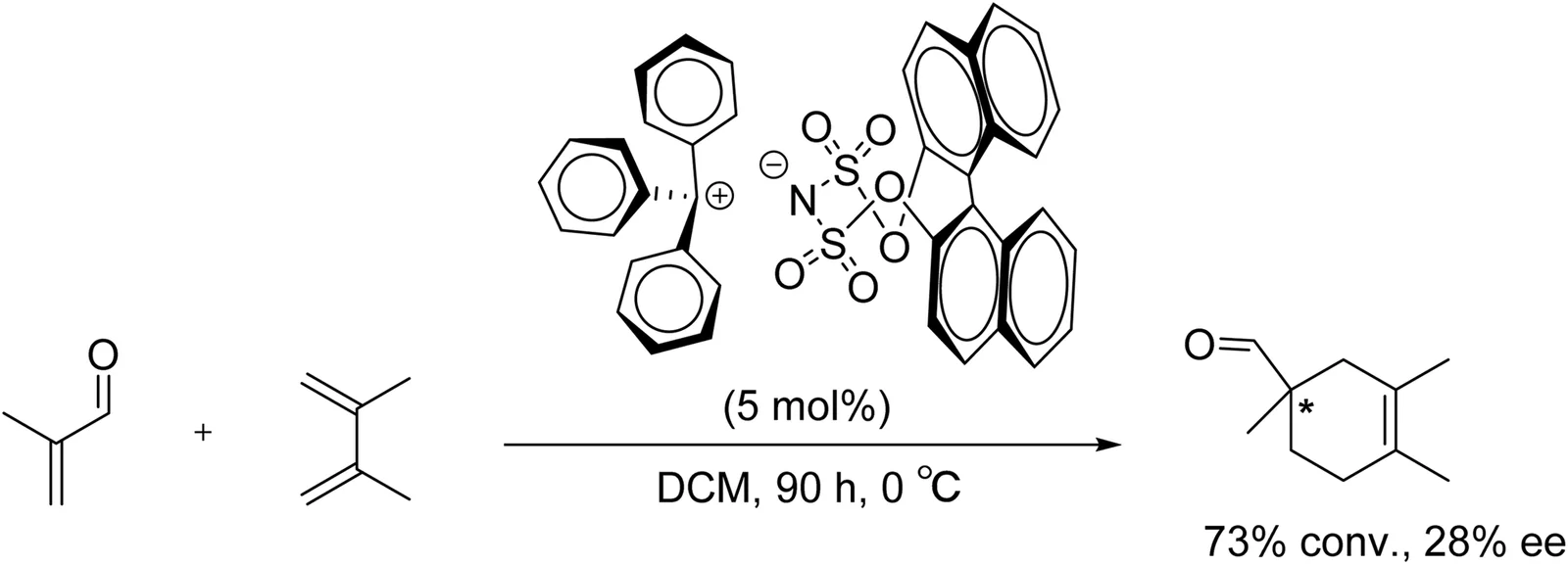
The Diels–Alder reaction of 2,3-dimethylbutadiene with methacrylaldehyde.

The Diels–Alder reaction of several dianions and dienophiles in the presence of chiral-anion-directed carbocation catalysis was investigated with limited success and significant enantioselectivity. According that, by the Diels–Alder reaction of 2,3-dimethylbuta-1,3-diene with methacrylaldehyde the corresponding Diels–Alder adduct was prepared in 73% of yield and 28% of enantiomeric excess as the best result after the optimization (Scheme 51).^31^

Halogenated solvents are more suitable in this reaction and for this reason 1,2-dichloroethane (DCE) as a solvent show the best effect on the reaction rate. Using a more nonpolar solvent such as toluene, which should favor close-contact ion pairs, resulted in a significant reduction in rate and only 15% of enantiomeric excess (ee%) under this condition.^31^

The Diels–Alder reaction of 2,3-dimethylbutadiene with methacrylaldehyde catalyzed by bis(sulfuryl)amide and TrCl in 1,2-dichloroethane as a solvent obtained the related Diels–Alder adduct in 75% of isolated yield and 29% of enantiomeric excess (ee%) after 96 h reaction time.^31^ By reducing the temperature to −20 °C, the reaction yield decreases and the enantiomeric excess increase. The conversion of the reaction investigated by ^1^H NMR spectroscopy of the crude reaction mixture and the enantiomeric excess (ee%) was obtained by chiral gas chromatography (chiral GC).^31^

In this study, chiral trityl cation/anion contact ion pairs have been used as active catalysts to induce chirality in Diels–Alder reactions with up to 53% ee according to the concept of chiral-anion-directed asymmetric carbocation catalysis. The important properties of carbocation Lewis acids and their recent catalytic application for some transformations, chiral-anion-directed asymmetric triarylmethyl carbocation catalysis can be considered as a potential strategy in organic synthesis.^31^

### The aldol reaction and Michael reaction catalyzed by a mixture of TrCl and tin(ii) chloride

**Scheme 52:**
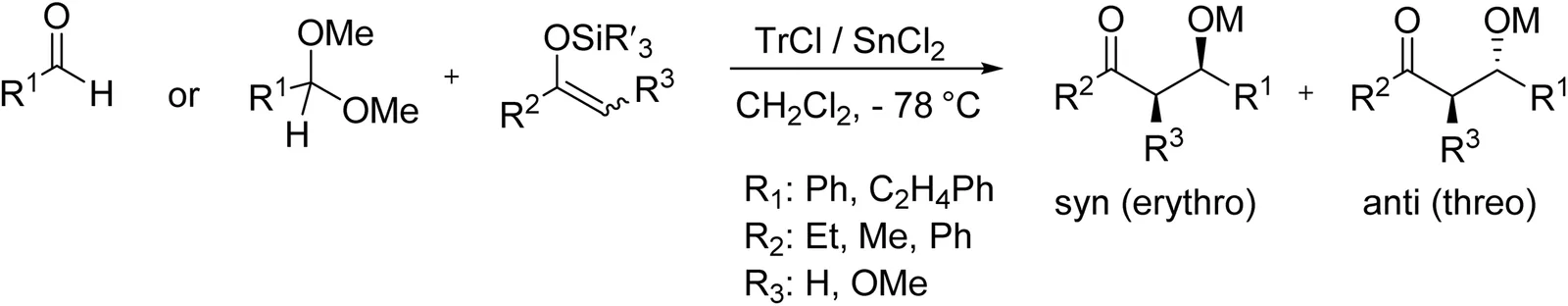
The aldol reaction catalyzed by a mixture of TrCl and tin(ii) chloride.

**Scheme 53:**
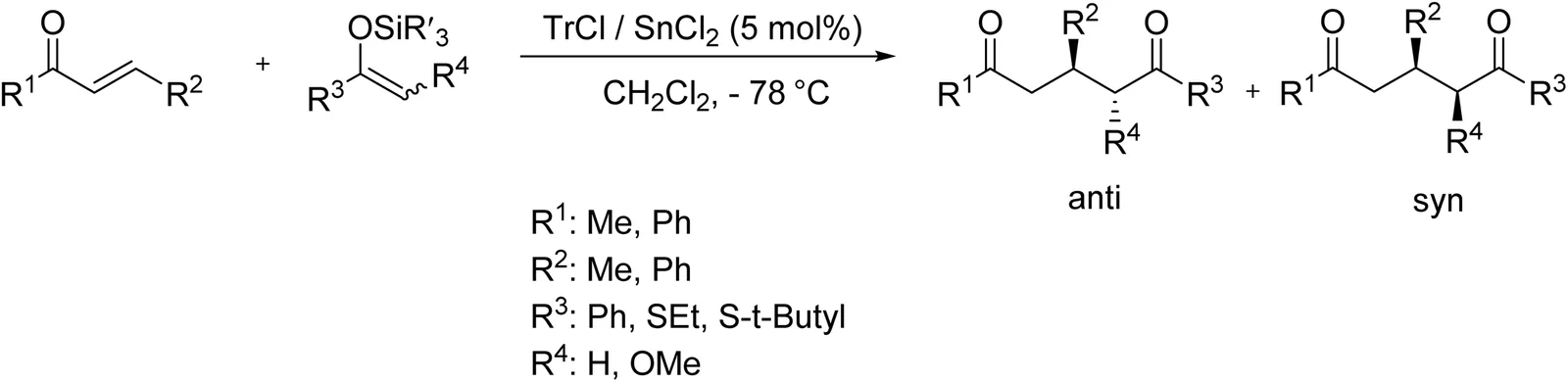
The Michael reaction catalyzed by a mixture of TrCl and tin(ii) chloride.

A catalytic system composed of TrCl and tin(ii) chloride in dichloromethane has been reported to promote both aldol and Michael-type reactions of silyl enol ethers. In this system, silyl enol ethers undergo aldol reactions with aldehydes or acetals, as well as Michael additions to α,β-unsaturated ketones, at −78 °C (Schemes 52 & 53).^32^

In this work, a catalytic system has introduced by the addition of TrCl and tin(ii) chloride in dichloromethane as a yellowish mixture produces an active triphenylmethyl carbocation species to proceed the aldol and Michael reaction. This catalytic system was prepared using a neutral molecule and weak Lewis acid using 5–10 mol% of each of these reagents. The combination of TrCl and tin(ii) chloride generated triphenylmethyl carbocation species which can activate electrophile easily. TrCl and tin(ii) chloride cannot carry out these reactions lonely. In the mentioned reactions, the valuable C–C bond formation can be completed under natural condition with a simple procedure.^32^

### The fabrication of 4-aryl-2*H*-chromenes in TrCl/TFA system

**Scheme 54:**
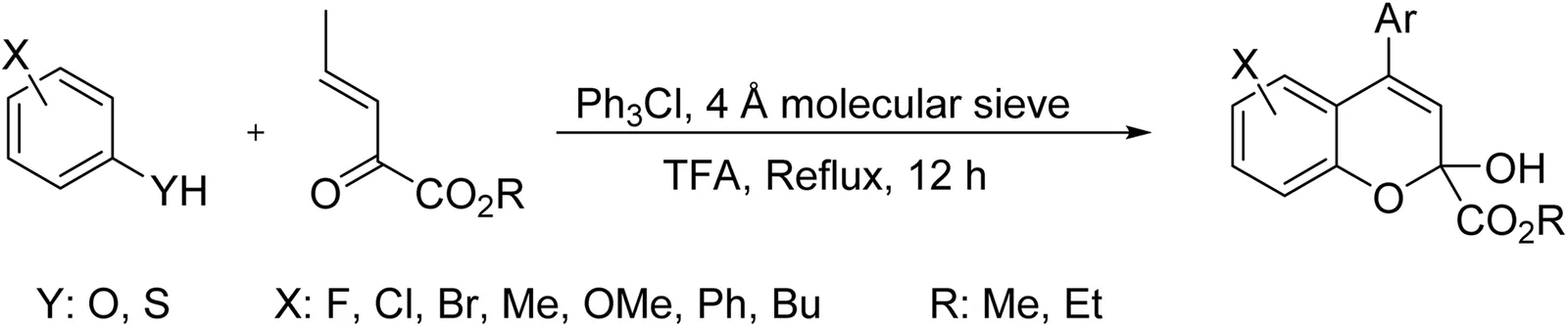
The synthesis of 4-aryl-2*H*-chromenes in TrCl/TFA system.

A cascade reaction for the synthesis of 4-aryl-2*H*-chromenes has been reported *via* the treatment of *β*,*γ*-unsaturated *α*-ketoesters with phenols or thiophenols in the presence of TrCl and 4 Å molecular sieves in refluxing trifluoroacetic acid afforded the corresponding products in high yields (Scheme 54).^33^

**Scheme 55:**
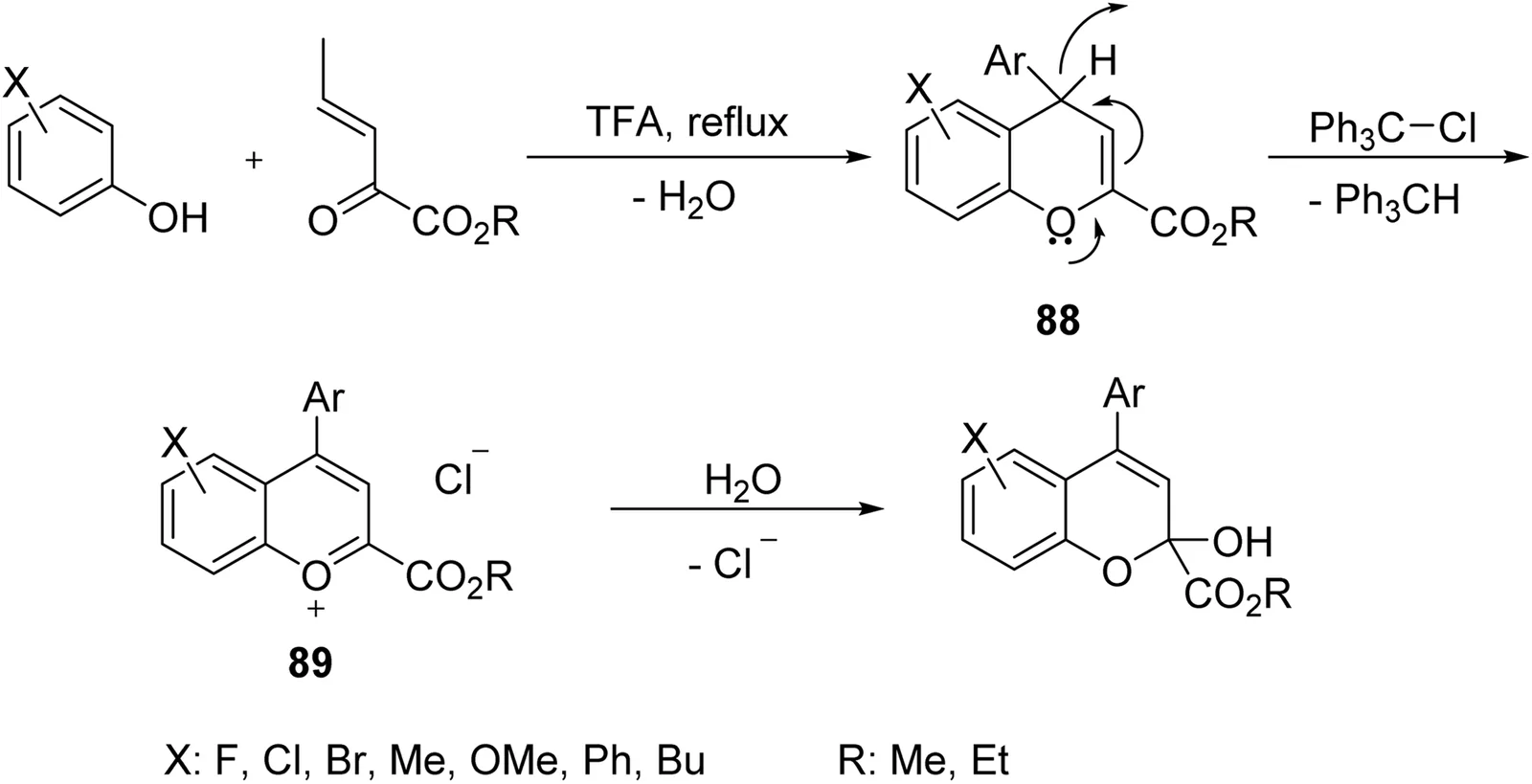
The mechanism for the production of 4-aryl-2*H*-chromenes in TrCl/TFA system.

In the first step in this synthesis, Friedel–Crafts alkylation and cyclodehydration processes was carried out. In the next part, by the reaction of the prepared intermediate (88) with TrCl in deuteron trifluoroacetic acid under reflux condition, hydride transfer was carried out as an anomeric effect and intermediate (89) was prepared. In this step, triphenyl methyl carbocation which *in situ* generated from TrCl abstracted hydride anion from intermediate (88). Finally, by the reaction of intermediate (89) with one molecule of water the expected 4-aryl-2*H*-chromene was obtained (Scheme 55).^33^

### Carbonyl-ene cyclization reaction catalyzed by TrCl

**Scheme 56:**
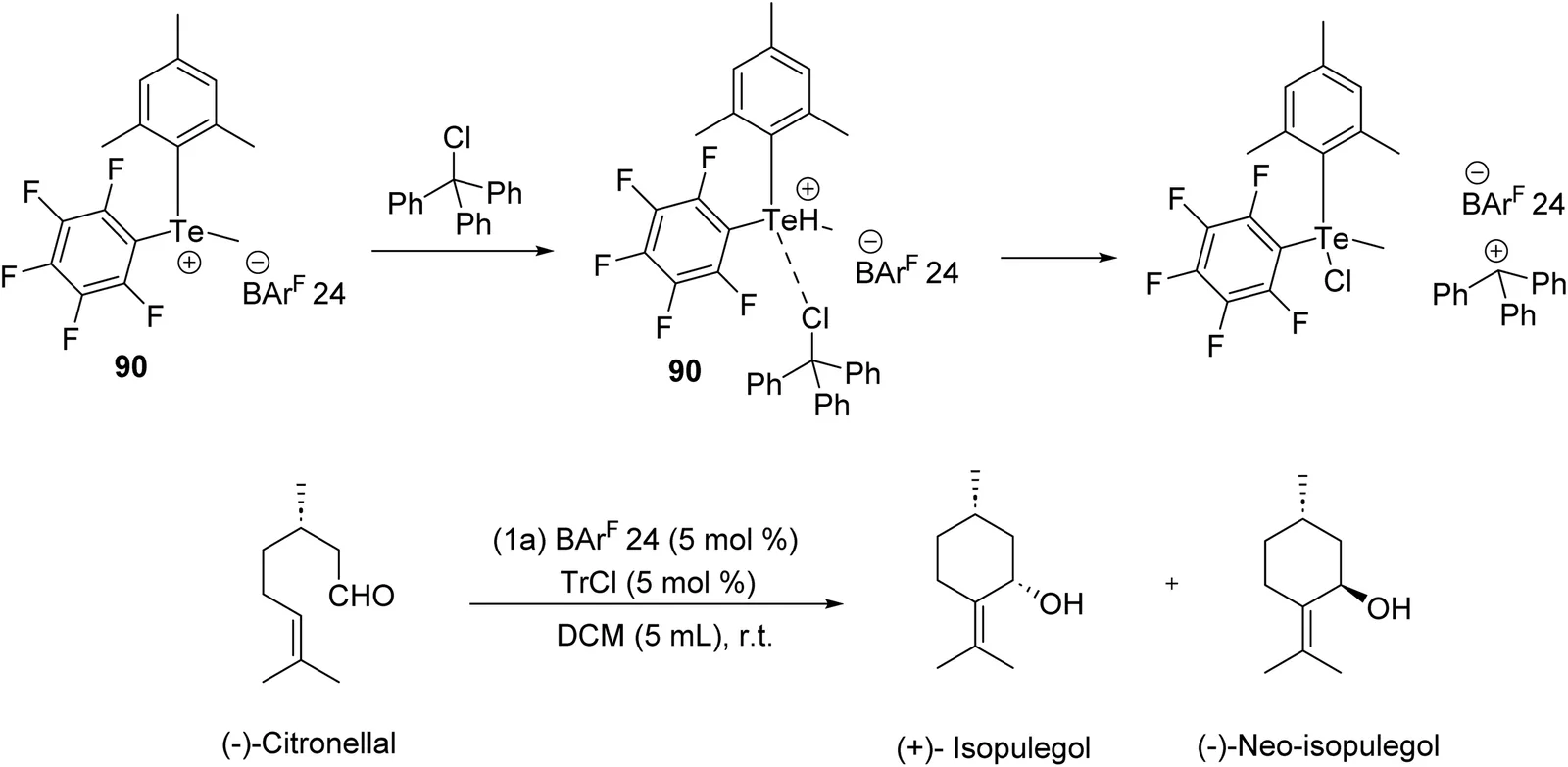
Carbonyl-ene cyclization reaction catalyzed by TrCl.

Catalytic application of trityl chloride next to telluronium salts with non-coordinative counter anions such as BAr^F^ 24 is reported in order to chloride abstraction from TrCl for *in situ* generation of trityl cation to use it as a challenging Lewis acid catalyst in the carbonylene cyclization of citronellal to give (+)-isopulegol and (−)-neo-isopulegol (Scheme 56) under an argon atmosphere.^34^ This work is another report of catalytic application of trityl chloride for C–C bond forming.

Telluronium salt (90) with different counter anions was used in carbonyl-ene cyclization reaction of (−)-citronellal using catalytic molecular equivalents of trityl chloride at room temperature in dry dichloromethane under an argon atmosphere. In the presence of 5 mol% of the cocatalytic system TrCl/BAr^F^ 24, the corresponding products was produced in 76% yield and 77 : 23 diastereoselectivity from the diastereoisomers (+)-isopulegol and (−)-neo-isopulegol after 24 h.

## Conclusion

Based on the results obtained, trityl carbocation, which is *in situ* produced in the reaction media, can be successfully used as a mild Lewis acid and homogeneous catalyst at low temperature and acid-sensitive environment in many multi-component organic reactions and also carbon–carbon bond formation reactions. In some reactions, some metal salts such as tin and tellurium salts have been used in order to catalytically activate trityl chloride and facilitate the separation of chloride anion from this compound. Also, by designing host–guest systems, the life time of trityl carbocation in the role of guest has been increased and better condition for catalysis of the reaction by this cation have been provided.

## Conflicts of interest

There are no conflicts to declare.

## Data Availability

No primary research results, software or code have been included and no new data were generated or analysed as part of this review.
